# A systems approach reveals distinct metabolic strategies among the NCI-60 cancer cell lines

**DOI:** 10.1371/journal.pcbi.1005698

**Published:** 2017-08-14

**Authors:** Maike K. Aurich, Ronan M. T. Fleming, Ines Thiele

**Affiliations:** Luxembourg Center for Systems Biomedicine, University of Luxembourg, Esch-Sur-Alzette, Luxembourg; University of California Riverside, UNITED STATES

## Abstract

The metabolic phenotype of cancer cells is reflected by the metabolites they consume and by the byproducts they release. Here, we use quantitative, extracellular metabolomic data of the NCI-60 panel and a novel computational method to generate 120 condition-specific cancer cell line metabolic models. These condition-specific cancer models used distinct metabolic strategies to generate energy and cofactors. The analysis of the models’ capability to deal with environmental perturbations revealed three oxotypes, differing in the range of allowable oxygen uptake rates. Interestingly, models based on metabolomic profiles of melanoma cells were distinguished from other models through their low oxygen uptake rates, which were associated with a glycolytic phenotype. A subset of the melanoma cell models required reductive carboxylation. The analysis of protein and RNA expression levels from the Human Protein Atlas showed that IDH2, which was an essential gene in the melanoma models, but not IDH1 protein, was detected in normal skin cell types and melanoma. Moreover, the von Hippel-Lindau tumor suppressor (VHL) protein, whose loss is associated with non-hypoxic HIF-stabilization, reductive carboxylation, and promotion of glycolysis, was uniformly absent in melanoma. Thus, the experimental data supported the predicted role of IDH2 and the absence of VHL protein supported the glycolytic and low oxygen phenotype predicted for melanoma. Taken together, our approach of integrating extracellular metabolomic data with metabolic modeling and the combination of different network interrogation methods allowed insights into the metabolism of cells.

## Author summary

Altered metabolism is characteristic for many human diseases including cancer. Disease progression and treatment efficacy vary between patients. Hence, we need personalized approaches to define metabolic disease phenotypes. This definition will enable us to unravel the underlying disease mechanisms and to treat individuals more efficiently. Computational modeling increasingly supports the analysis of disease mechanisms and complex data sets. The interpretation of extracellular metabolomic data sets is particularly promising since this data type is proximal to the actual metabolic phenotype altered in human diseases. Moreover, it might enable us to directly interpret disease states from serum samples in the future. Herein, we took a first step towards this ambitious goal. We generated a large set of cancer metabolic models from extracellular metabolomic data and computationally stratified the models based on their metabolic characteristics into different phenotype groups. Melanoma emerged as an interesting example of how our approach can provide insights into the intracellular metabolism from extracellular measurements. Taken together, this work paves the way to generate condition-specific models from extracellular metabolomic data and demonstrates the many ways by which distinct phenotypes can be stratified and phenotype-specific intervention targets can be predicted.

## Introduction

Aerobic glycolysis indicates the incomplete oxidation of glucose to lactate under normoxic conditions [1] and has been a focus of cancer research in recent decades [2]. However, cancer cells are increasingly thought to employ heterogeneous metabolic strategies beyond aerobic glycolysis [3–6]. Many cancer cells generate substantial amounts of energy through mitochondrial oxidative phosphorylation [2, 7, 8]. Moreover, cancer cells use additional fuels, such as glutamine and fatty acids, to support proliferation [3, 9]. These carbon sources can be used in different ways, e.g., different parts of the tricarboxylic acid (TCA) cycle can be employed for glutaminolysis [5, 8, 10, 11]. Reductive carboxylation involves only two TCA cycle reactions that run in reverse direction without producing energy, whereas glutaminolysis in the forward direction does yield energy [5, 8, 11]. In addition to various metabolic strategies, cancer cells display robustness towards environmental changes, such as, nutrient supply or oxygenation [12–14]. Even though these differences in metabolic phenotypes are known to exist, the variance in the metabolism of cancer cell lines has not been exhaustively analyzed using extracellular metabolomic data.

Liquid chromatography-tandem mass spectrometry (LC-MS) was used to determine the metabolites that were consumed and released by the cancer cell lines included in the NCI-60 panel of the National Cancer Institute’s (NCI’s) Developmental Therapeutics Program (DTP; http://dtp.nci.nih.gov) [15]. By combining the obtained metabolomic profiles with doubling times and transcriptomic data, rapid proliferation was associated with cellular glycine requirements [15]. However, most of the intracellular pathways that gave rise to distinct metabolomic profiles remained undetermined.

Metabolism can be investigated using constraint-based modeling [16, 17], which involves the application of physico-chemical principles and often assumes the system to be in a steady-state [16]. Limitations on metabolite uptake and secretion rates can be added to the model to increase the precision of the predictions by eliminating network states that exceed these constraints [18]. A reconstruction of the human metabolism is readily available [19, 20], and numerous analytical methods are used to investigate the metabolic differences that arise due to the imposed constraints [21, 22]. Metabolomic data derived from body fluids and cell culture supernatant have previously been integrated with metabolic reconstructions [7, 23, 24].

One existing challenge in the integration of extracellular metabolomic data is incomplete data. Analytical techniques identify only a subset of the metabolome due to the chemical diversity among small molecules and because the analysis is often a priori limited to a defined set of targeted metabolites [25]. Hence, the information on which substrates are taken up by the cells is incomplete. Similarly, the management of data derived from cells grown in serum is difficult because the quantitative and qualitative composition of the serum is unknown. However, the quality of computational predictions depends on the extent to which a model’s solution space can be reduced by integrating available data. Ideally, only biologically relevant network states would remain to be investigated [18]. Novel approaches are necessary to overcome these difficulties and enable the rapid classification of metabolic phenotypes based on metabolomic profiles. Such approaches could have a broad impact on many biological fields including biomedicine.

We developed a novel method termed minExCard to complete the uptake and secretion profile, by predicting a minimal set of metabolite exchanges in addition to the ones measured, to complete the metabolome. We applied the method to the comprehensive targeted extracellular metabolomic data set from Jain et al., which was generated from the NCI-60 cell lines grown in medium enriched with serum [15].

Using minExCard we generated 120 condition-specific models from extracellular metabolomic data. Our models utilized different biochemical routes to supply the cells with energy and were distinctively affected by network perturbations. We distinguished different oxotypes based on the range of allowable oxygen uptake rates. We identified a distinctive tissue pattern for melanoma cell lines that was supported by protein and RNA expression levels from melanoma cell lines and primary melanoma. This work demonstrates how analysis of extracellular metabolomic data in the metabolic model context, and the combination of multiple analysis strategies, can lead to unprecedented insight into cell metabolism.

## Results

### Generation of heterogeneous condition-specific cancer cell line models

**Fig 1:**
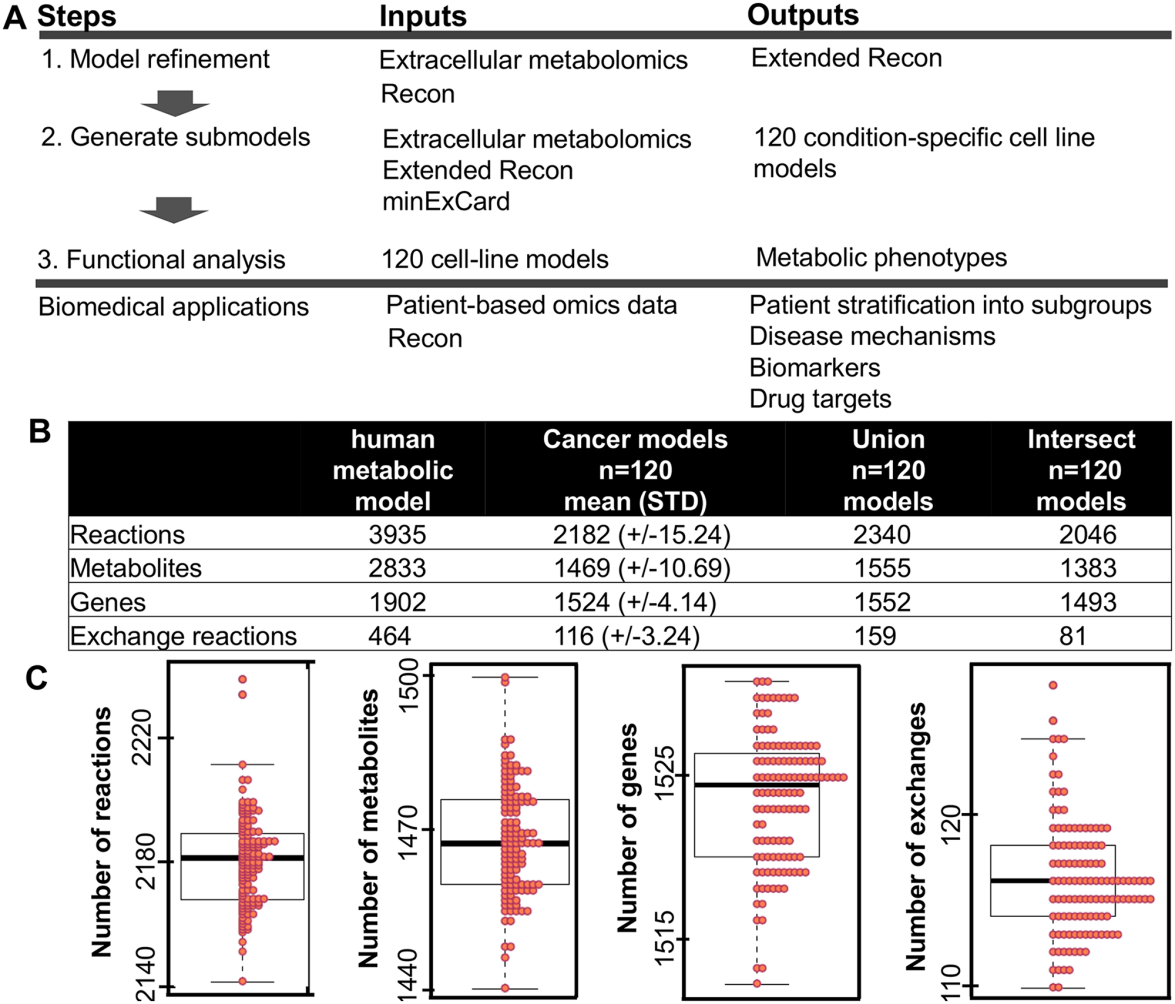
Metabolic models provide a context for the analysis of metabolomic data. **A** 1. The refinement step denotes the addition of transport and exchange reactions to enable the uptake and secretion of the metabolites detected in the metabolomic profiles of the NCI-60 cell lines [15]. 2. The condition-specific cell line models were generated using minExCard. In total, 120 models (NCI-60 multiplied by 2) were generated from published metabolomic data and the extended metabolic model. 3. The models were analyzed using a set of computational methods. Based on the computational results, the models were divided into different metabolic phenotypes, and drug targets were predicted for each individual model. The approach is applicable to a variety of biomedical applications. An analysis of tumor or patient-specific omics data could be used to stratify disease phenotypes and to predict personalized disease intervention strategies. **B** Differences in the number of reactions, metabolites, and genes across a large set of models. **C** Distribution of the number of reactions, metabolites, genes, and exchange reactions among the 120 cell line models.

Published metabolomic profiles comprising the uptake and secretion of metabolites from and into the culture medium were integrated with the metabolic model (Fig 1A) [15]. The metabolomic data consisted of two samples per cell line. Because there was considerable variation between samples (Fig A in S1 Text), we generated one condition-specific cell line model for each sample rather than averaging the data for each cell line. The metabolome is dynamic and constitutes a snap-shot of the phenotype elicited by the cultivated cells over the duration of the experiment and under a specific set of environmental conditions. We refer to the models as condition-specific since they are tailored only according to the metabolomic profiles. Generic cell-line specific models would need to be generated from data sets of different experimental conditions and the existing literature for the same cell line, to ensure that it can carry out all the functions observed for these cells under any set of environmental conditions. To generate a condition-specific model, the global model was constrained using the metabolite uptake and secretion rates measured for the respective samples. Next, a minimal set of, on average 17 ± 3, exchange reactions needed to sustain a minimal growth phenotype (V_biomass,min_ = 0,008 U) together with the imposed uptake and secretion rates were identified based on the model structure by minimizing the number of exchange reactions (using minExCard). An analysis of the expression of genes associated with the metabolites additionally required in the MCF-7 models (which required the highest number of added exchange reactions), revealed that extracellular transport and metabolism of these added metabolites could indeed appear in MCF-7 cells (see S1 Text, S1A Table, [26]). However, the gene expression data was only used to validate the added exchanges, but not for the generation of the condition-specific models since the transcriptomic data originated from a different experiment than the metabolomic data. All other metabolite exchanges and internal reactions that were no longer used by the model were removed to produce an individual condition-specific cell line model for each sample (Fig 1A).

The 120 models differed with respect to the numbers of reactions, metabolites, and genes (Fig 1B and 1C, Fig B in S1 Text). Many of the models could substantially exceed the maximally possible growth rates expected for any human cell (S1B Table). The capability of the models to grow at realistic rates was analyzed by applying constraints on the biomass objective function based on reported growth rates (+/-20%) for the individual cell lines, and flux balance analysis revealed whether the model remained feasible with these constraint. Only 14 models were infeasible when constrained using the experimental growth rates (see S1 Text, S1C Table) because the feasible range of flux rates through the biomass reactions exceeded or did not reach up to the experimental growth rates, even when assuming a 20% error range. ACHN-2 and UACC-257 were limited to experimental growth rates just by the metabolite uptake and secretion profile and the minimal growth constraint (S1 Text, S1B and S1D Table). Considering a lower error of 5% or constraining both upper and lower bound to the growth rate, the ACHN-2 model became infeasible (S1D Table). The predicted growth rates for the HCT-116 models using sampling V_median,biomass_ = 0.038 U, corresponding to a doubling time of 18.2 hrs, deviated at most 7% from the growth rate reported by Jain et al. and others (S1D Table, [15, 27]). Taken together, the diversity of the models and their ability to predict realistic growth rates suggested that they were a good starting point to investigate metabolic heterogeneity between the cell lines.

### Distinct metabolic phenotypes

**Fig 2:**
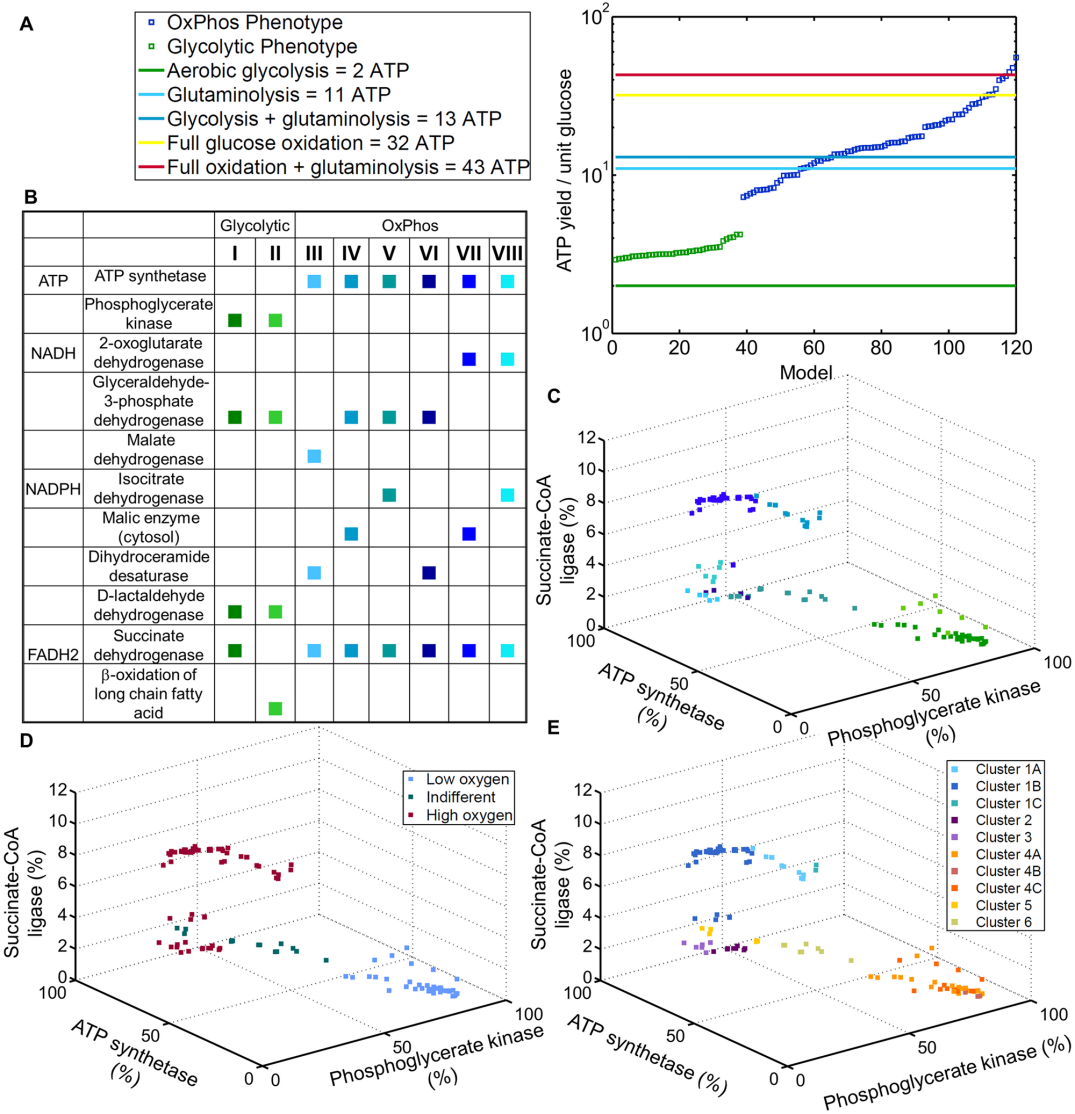
Distinction of the models. **A** Rank-ordered ATP yields achieved by the models describe a gradual increase rather than clusters around theoretical ATP yields. The spread of ATP yields highlights the metabolic heterogeneity between the 120 models. The cell lines use a mixture of pathways and metabolic fuels for ATP production, which explains that the predicted ATP yield can exceed the theoretical measures. Two major strategies for ATP production can be distinguished based on the ATP yields. The distinction lies in the higher contribution of either phosphoglycerate kinase (green squares) or ATP synthase (red squares) to the total ATP production. **B** A fine-grained division of the OxPhos models is achieved considering the production strategies of ATP, NADPH, NADH, and FADH2. The table lists the reactions contributing most to ATP, NADH, NADPH, and FADH2 production for each phenotype (I-VIII). **C** A three dimensional plot of the eight phenotypes with respect to the utilization of glycolysis, the TCA cycle, and oxidative phosphorylation. **D** Three different oxotypes are distinguished. The distinction between the OxPhos models (blue) is different from the phenotypic classification performed based on the energy and cofactor production strategies depicted above (see also S1 Text). **E** Six model clusters are distinguished according to each models’ ability to deal with environmental changes. Variations in glucose, glutamine, lactate, and oxygen lead to a distinct stratification of OxPhos models. Fig F in S1 Text shows different perspectives.

Metabolic strategies yield different amounts of ATP, e.g., full oxidation of glucose to CO_2_ can yield 32 ATP and aerobic glycolysis can yield two ATP [28, 29]. Herein, we used the ATP yield as an estimator for distinct pathway utilization. For this analysis, we divided the sum of flux through all reactions in the model that produced ATP by the individual glucose uptake. There was a large range of ATP yields across the models (Fig 2A, ATP yield: min = 2.92, max = 55.27, S1B Table) that exceeded the theoretical measure for aerobic glycolysis. An exact fit with the theoretical ATP yields was not expected because the models could use all substrates as defined by the uptake profile and ATP-producing reactions present in the condition-specific model and not only glucose (Fig A in S1 Text). As a sanity check, we tested for maximum ATP hydrolysis flux from only O_2_ and glucose as carbon source. ATP hydrolysis flux from glucose did not exceed the theoretical measures [28, 29] in any of the 120 cancer models (S1E Table). Upper bounds on exchange reactions were opened for the sanity check. Rank-ordered ATP yields nearly continuously increased and were occasionally interrupted between groups of models (Fig 2A, Fig C in S1 Text). One interruption was associated with the switch of the major ATP-producing reaction. Models with an ATP yield < 4.21 (’glycolytic’ models, n = 38, Fig 2A) produced the highest fraction of ATP through phosphoglycerate kinase (PGK). In contrast, models with an ATP yield > 7.26 produced ATP primarily via ATP synthase (’OxPhos’ models, n = 82, Fig 2A). Thus, the ATP yield and ATP production strategy divided the models into glycolytic and OxPhos phenotypes. The distinction of the models was significantly associated with the ratios of glucose uptake to lactate secretion (ttest, p< 0.01), and glucose uptake to glutamine uptake (ttest, p< 0.0002). Taken together, the distinction of the glycolytic and the OxPhos models emerged from the ratios of fluxes of metabolites, which are associated with the observed Warburg phenotype and, which were imposed on the models as individual flux constraints.

Consideration of differences in the utilization of the TCA cycle, i.e., ATP production of succinate-CoA ligase, enabled the further identification of two OxPhos subtypes (Fig D in S1 Text). This division was not obvious according to ATP yield (Fig E in S1 Text). In addition to ATP, cells need cofactors to support proliferation. Distinct strategies used in the models produced different cofactors and enabled the division of glycolytic models into two subtypes (Fig 2B and 2C, S1F–S1J Table). The two OxPhos subtypes were further subdivided into a total of six subtypes (Fig 2B and 2C, S1J Table). The glycolytic subtypes differed only in the major FADH2-producing reaction (Fig 2B, I and II). Two OxPhos subtypes were associated with high TCA cycle contribution to ATP production, which was associated with a high utilization of cytosolic malic enzyme as a leading NADPH source (Fig 2B, IV and VII). The four remaining OxPhos subtypes predominantly used either isocitrate dehydrogenase (IDH, Fig 2B, V and VIII) or dihydroceramide desaturase (Fig 2B, III and VI) for NADPH production. Glyceraldehyde-3-phosphate dehydrogenase was the primary NADH producer in OxPhos models with relatively more glycolysis-based ATP production, whereas 2-oxoglutarate dehydrogenase was favored in models with a higher contribution of ATP synthase (Fig 2C). Thus, the predicted strategies for cofactor production enabled further refinement for the classification of glycolytic and OxPhos models.

### Robustness towards genetic and environmental perturbation

**Fig 3:**
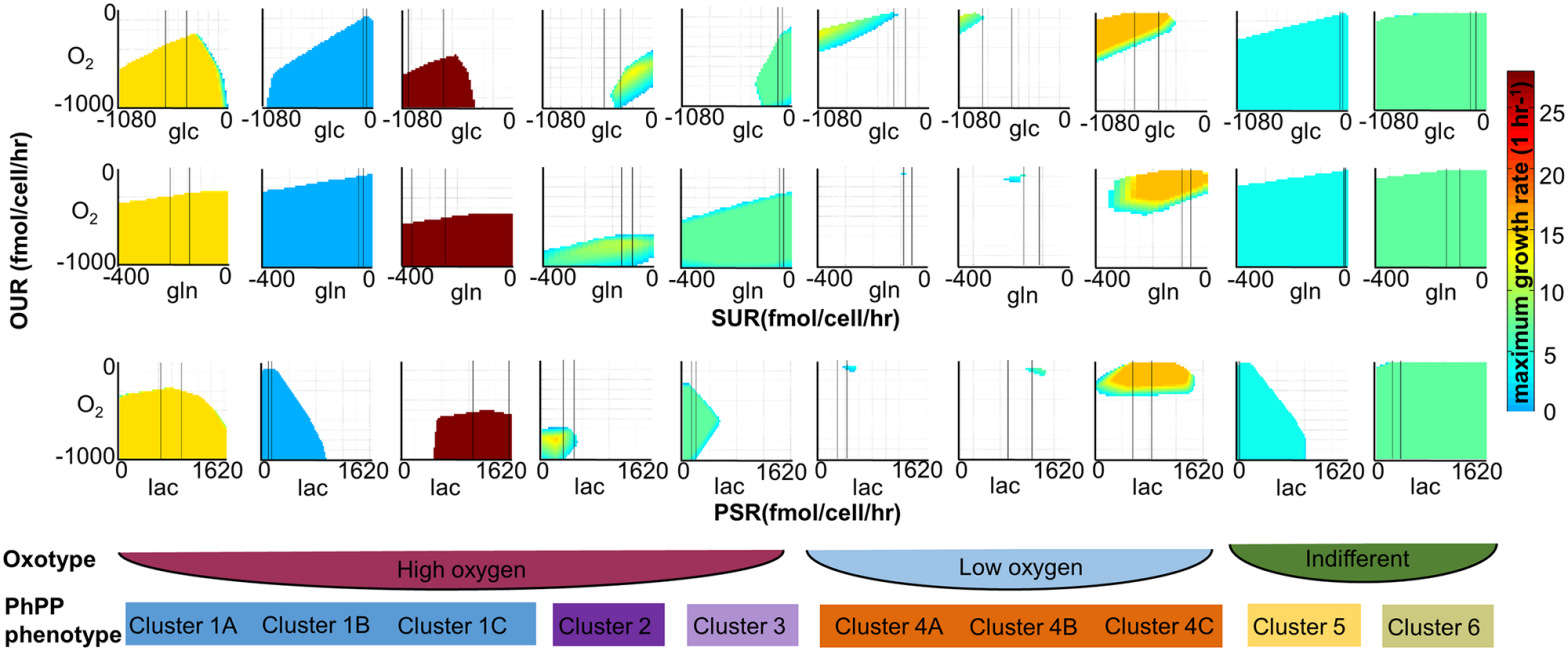
Six model clusters are distinguished according to the models’ robustness towards environmental changes. Heatmaps display results for one model from each cluster (and subcluster). Lines in the heatmaps indicate the constraints imposed on the exemplified model. Lac = lactose, glc = glucose and gln = glutamine.

Thus far, we stratified the models based on the imposed constraints and the distinct use of central metabolic pathways. In the following, we predict the behavior of each model towards environmental and genetic perturbations. Fluctuations of nutrients and oxygen supply during transformation shape the individual metabolic network and may influence the robustness of cancer cells towards environmental changes [30]. Variations of glucose uptake, glutamine uptake, oxygen uptake, and lactate secretion (phenotypic phase plane analysis (PhPP)) led to two major observations [31]. First, the size and form of the solution spaces varied across models (Fig 3). Using the form and size of the solution spaces as visual clues (Fig G in S1 Text), we divided the models into six distinct clusters (Figs 2E and 3, S1K Table, S1 Text). Second, the solution space, which contains all possible network states and which was defined by variations in oxygen uptake, divided the models into three groups (Fig 2D) (i) Glycolytic models could only grow at low oxygen uptake rates (Figs 2D and 3 cluster 4). The group of OxPhos models comprised (ii) models growing only at high oxygen uptake rates (Figs 2D and 3 cluster 1–3) and (iii) models that were indifferent with respect to oxygen uptake rates (Figs 2D and 3 cluster 5–6). The latter two groups provided a separation of the OxPhos models that was distinct from the previous analysis. Thus, the models could be further divided according to their robustness towards oxygen uptake.

In silico gene knock-outs can predict novel drug targets [32]. Single gene deletion of 1215 unique human genes (all isozymes of one gene were constrained to zero at once) was performed for each of the 120 models. The number of essential genes varied across models (min = 132, max = 272, S1B Table) and was not associated with any phenotype. A total of 55 genes were essential to all models and could constitute metabolic targets for all previously defined phenotypes (S1L Table). These numbers of essential genes predicted by our models were higher compared to those predicted for generic cell-or tissue specific models. This was caused by the vast reduction of exchange reactions and fixed uptake and secretion fluxes, which prevented that upon a gene knock-out, the models could switch to using different metabolic fuels or pathways connected to changes in gene expression. The flux ranges and the direction of flux of the exchange reactions were fixed, causing any reaction that was linked to the exchanges to become essential for the model. Whether a gene was essential under changing environmental conditions and whether cells in vivo could evade the effect by changing the metabolic pathways used to generate energy, cannot be answered by our models. However, models build from transcriptomic data could be used instead. Such models have previously revealed the switch to pathways requiring higher oxygen uptake when glycolytic enzymes were inhibited [33]. However, the condition-specific models, which are ‘frozen’ to the metabolic properties elicited at the time, highlight inhibition of which genes necessitate changes in metabolic flux and changes in gene expression.

Cancer cells use the TCA cycle in different ways [5, 8]. Reductive carboxylation involves the TCA cycle reactions isocitrate dehydrogenase and aconitase, and occurs in the mitochondria or the cytosol. The gene *IDH1* encodes the cytosolic isocitrate dehydrogenase and the gene *IDH2* encodes the mitochondrial isocitrate dehydrogenase. Interestingly, in silico *IDH2* knock-out terminated growth in four models (SK-MEL-28, SK-MEL-28-2, MALME-3-2, and BT-549) and reduced growth in 12 additional models. A flux variability analysis (FVA) revealed that the four models had to employ reductive carboxylation (S1M Table [34], whereas this pathway remained optional for the other models even when constrained to experimental growth rates (S1D Table). In agreement with an observed increase in reductive carboxylation under hypoxic conditions [5], a reduction of the oxygen uptake rate (lb = ub = −100 fmol/cell/hr) rendered 14 additional models dependent on reductive carboxylation (S1M Table). Fifteen models, including the four reductive carboxylation models, belonged to PhPP cluster 4, which was characterized by a heavily constricted solution space at low oxygen uptake rates compared with, e.g., the cluster 4C models (Fig 3). The remainder belonged to cluster 1B. Our models were therefore not only able to predict reductive carboxylation but also able to further reproduce the co-occurrence of low oxygenation and reductive carboxylation in cancer cell lines. Phosphoglycerate dehydrogenase (*PHGDH*) was another essential gene shared among the four models with obligate reductive carboxylation. Interestingly, SK-MEL-28 and MALME-3M had previously been associated with amplifications of PGDH due to 1p12 gain [4, 35]. The correct prediction of the dependency of SK-MEL-28 and MALME-3M on PHGDH provides additional support for the presented approach and for the predicted dependency of SK-MEL-28 on reductive carboxylation.

**Fig 4:**
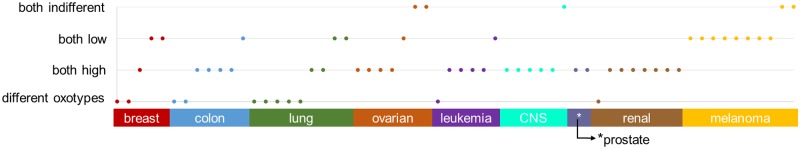
Oxotype of model pairs. Replicate models of the same cell line predominantly share the same oxotype. Only 11 model pairs have distinct oxotypes (different oxotypes). A tissue pattern becomes apparent for the melanoma cell lines. Melanoma cell lines predominantly have a low oxotype.

Because the oxotype played an essential role in determining the phenotype and because tissues are known to be differentially oxygenated [36], we questioned whether tissue origin impacted the oxotype of the cancer. In total, 49 cell line model pairs had the same oxotype (Fig 4). Breast, colon, and non-small cell lung cancer models were spread across oxotypes. Leukemia, prostate, renal, and CNS cell line models predominantly depended on high oxygen uptake rates. In contrast, melanoma cell lines were clearly separated from the other cell lines by predominantly relying on low oxygen uptake rates (Fig 4). Thus, the oxotypes enabled us to distinguish melanoma cell lines from other cancer cell lines.

### Validation of the emerging tissue pattern

Most melanoma models were predicted to be glycolytic and having a low oxotype (Fig 4, S1J and S1K Table). A reverse flux through the TCA cycle was essential for a small subset of melanoma models without additional constraints limiting the oxygen uptake. To validate that melanomas indeed use the mitochondrial isocitrate dehydrogenase, we analyzed protein abundance and RNA expression data from the Human Protein Atlas [37]. IDH1 protein abundance was low or not detectable in normal skin cell types (hypergeometric p (x = 5) = 0.047, Table 1, ^1^), skin cancer, and melanoma. In comparison, IDH2 protein levels were medium in normal skin cell types (hypergeometric p (x = 5) = 0.006, Table 1, ^2^) and detected in more than 50% of the skin cancers and melanomas (Table 1). Thus, the data supported a prevalence of IDH2 for normal skin cell types, skin cancers, and melanoma at the protein level.

**Table 1:** Protein abundance data from the human protein atlas supports the predicted tissue pattern. The footnotes indicate which cell types or cell lines were considered in the hypergeometric tests. The hypergeometric probabilities (1,2,3) are provided in the main text.

Cell line	VHL	IDH1	IDH2	HIF1*α*
Normal skin fibroblasts	Uncertain	Not detected^1^	Medium^2^	Low^3^
Normal skin keratinocytes	Uncertain	Not detected^1^	Medium^2^	Low^3^
Normal skin Langerhans	Uncertain	Not detected^1^	Medium^2^	Low^3^
Normal skin melanocytes	Uncertain	Not detected^1^	Medium^2^	Low^3^
Normal skin epidermal cells	Uncertain	Not detected^1^	Medium^2^	Low^3^
Skin cancer (patients)	12x Not detected	1x Low, 10x Not detected	2 x High, 5x Medium, 5x Not detected	2x Medium, 6x Low, 4x Not detected
Melanoma (patients)	12x Not detected	1x Low, 11x Not detected	1x High, 4x Medium, 2x Low, 5x Not detected	4x Medium, 4x Low, 4x not detected
Renal cancer (patients)	7x Medium, 4x Low, 1x Not detected	1x High, 1x Low, 10x Not detected	4x Medium, 1x Low, 7x Not detected	1x Medium, 3x Low, 7x Not detected

Reductive carboxylation has been associated with the loss of the von Hippel-Lindau tumor suppressor (VHL) in renal cancer cell lines [38]. HIF1*α* protein is no longer degraded, which is associated with the expression of glucose transporters and glycolytic enzymes [39, 40]. Since the process of HIF stabilization is connected to hypoxia, this process has also been referred to as pseudo-hypoxia [5]. To validate the predicted glycolytic phenotype and the low ‘oxotype’, we analyzed HIF1*α* and VHL protein, and RNA levels. HIF1*α* protein abundance was low in normal skin tissue (hypergeometric p(x = 5) = 0.019, Table 1, ^3^) and low or medium in the majority of skin cancers and melanomas (Table 1). HIF1*α* RNA expression was overall high in human melanoma and epidermoid carcinoma cell lines (Table 2).

**Table 2:** RNA expression data from the human protein atlas revealed low levels of VHL and high levels of HIF1*α* in skin cell lines that are not part of the NCI-60 panel. The predicted phenotypes are listed for all NCI-60 cell lines in the data set. None of the skin cancer cell lines in the data set was a NCI-60 cell line. CNS = Central nervous system.

Cells	Cancer	Predicted phenotype	VHL	HIF1*α*
A-431	Skin (human epidermoid carcinoma)		Low^1^	High
SK-MEL-30	Skin (human melanoma)		Low^1^	High
HaCaT	Skin (immortalized, non-tumorigenic human keratinocyte)		Low^1^	Medium
WM-115	Skin (human melanoma)		Low^1^	High
A549	Lung	Glycolytic	Low	High
K-562	Leukemia	Glycolytic	Medium	Low
HL-60	Leukemia	OxPhos	Medium	Low
MOLT-4	Leukemia	OxPhos	Medium	Medium
PC-3	Prostate	OxPhos	Medium	High
RPMI-8226	Leukemia	OxPhos	Medium	Low
MCF-7	Breast	OxPhos	Low	Medium
U-251 MG	CNS	OxPhos	Low	High
Normal	Skin		Medium	Medium
Normal	Kidney		Medium	High

The VHL protein detection was unreliable in all normal skin cell types (Table 1). Interestingly, VHL protein was not detected in skin cancers or in melanomas (Table 1). The absence of VHL was even more distinctive in skin cancers as compared to renal cancers where VHL levels were medium or high in 7 out of 12 patient samples (Table 1). Moreover, RNA expression was low in two melanoma cell lines, an epidermoid carcinoma, an immortalized normal keratinocyte cell lines (hypergeometric p(x = 4) = 0.044, Table 2, ^1^), and A549 cells, which were predicted to be low oxotype (Table 2). Hence, the lack of VHL emerged as a prominent feature of normal skin and melanoma.

#### Representation of the genotype-phenotype relationship

Reductive carboxylation has been observed in renal cancers with VHL mutations under normal oxygen conditions [38]. The NCI-60 cell line 786-O carried a VHL mutation [41]. If correctly predicting the genotype-phenotype relationship, our 786-O models should predict reductive flux through the TCA cycle. We predicted that the two 786-O models could carry reverse flux through the IDH2 and forward flux through the IDH1. Hence, the models allowed the metabolic phenotype that would be expected for 786-O cells (S1N Table)). However, the constraints were not determining enough to enforce reductive carboxylation. Reductive carboxylation is associated with hypoxia in cancer cells and the loss of VHL is associated with the hypoxic-like response and the metabolic pathways are rewired accordingly [5]. Hence, we next explored if the expected phenotype could be achieved by limiting the oxygen uptake flux. Cell line specific oxygen uptake rates were not provided by [15] but restricted to the same upper limit in all models (see methods section). As a consequence, the 786-O models could consume high oxygen uptake fluxes (786-O V_O2,median_ = −992.49 U, and 786-O-2 V_O2,median_ = −992.59 U). We limited the oxygen uptake flux in the 786-O models to the minimum (786-O V_O2,min_ = −153.03 U, and 786-O-2 V_O2,min_ = −151.16 U, S1O Table). As a consequence, the models predicted a net reverse flux through IDH2, i.e., the 786-O models could no longer carry flux through IDH1 and IDH2 flux had to be backwards (786-O V_aconitase,min,max_ = −19.56 U, and 786-O-2 V_aconitase,min,max_ = −17.27 U, S1O Table). Hence, after restricting the oxygen uptake, the 786-O models were obliged to net reductive carboxylation. Taken together, the metabolomic constraints were not restrictive enough to limit the 786-O models to the expected phenotype, i.e., reverse IDH2 flux. This result underlines the value of measurements of oxygen consumption for the prediction of cancer cell phenotypes.

#### The common denominator of the different cancer phenotypes

Despite predicting what dissociates the cancer models, the condition-specific models can also be used to identify common traits. The in silico gene knock-out analysis yielded 55 genes that were essential to all 120 models. Many of the model genes are associated with more than one reaction and disabling flux through only some of these reactions renders the model unable to grow. Disabling individual reactions associated with the 55 essential genes at a time let to the identification of 16 reactions that were essential for all models (S1P Table).

The models were built by imposing constraints that forced them to consume or secrete metabolites. Additionally, we enforced a minimal growth constraint during the model building. Hence, those genes and reactions that the models needed to comply with because of the imposed constraints are expected to be essential. From the set of 16 essential reactions, four reactions were essential also in the unconstrained model. Additional nine reactions directly involved metabolites that were part of the biomass composition or the uptake and secretion profiles that had been integrated with the models. Three essential reactions comprised a path leading to the production of succinyl-CoA by propionyl-CoA carboxylase for the TCA cycle. Interestingly, itaconic acid inhibits this enzyme in bacteria [42], and has been suggested as cancer growth modifier, because of its impact on substrate level phosphorylation and to mitochondrial energy generation [43]. Itaconic acid production, which is induced by IRG1 expression, is produced by tumor cells originating from macrophages and related cell types [44, 45]. On the contrary, toxicity of a polymer containing itaconic acid, fumaric acid, and 1, 4-butanediol specifically designed as drug delivery vehicle with inherent anticancer activity has been observed for the human breast cancer cell line MCF-7 [46]. Taken together, the prediction of the common essential reaction set highlighted propionyl-CoA carboxylase as potential target to interfere with the different metabolic phenotypes.

## Discussion

Biochemical and molecular biological methods increasingly generate large omics data sets, which require adequate methods to facilitate their interpretation. Herein, we integrated published metabolomic data into the network context to obtain condition-specific metabolic models for the NCI-60 cell lines. The metabolic models were generated to be consistent with the measured data and known human biochemistry. We used this compendium of metabolic models to explore the metabolic strategies followed by various cancer cell types. Our main results are as follows: (1) minExCard enabled the integration of quantitative extracellular metabolomic data while using the context of the metabolic model to complete metabolic exchange profiles; (2) distinct biochemical routes were utilized by the different cancer models to supply the cells with energy; (3) most notably, the models were divided into oxotypes, which distinguished the allowable oxygen uptake rates of the models and distinguished melanoma from other cancers; and (4) the predicted tissue pattern was supported by protein and RNA levels of melanoma cell lines and primary melanoma tissue. Taken together, our study furthers the interpretation of extracellular metabolomic profiles in the context of metabolic models and provides biological insight into the metabolism of NCI-60 cancer cell lines that could not have been drawn from the data alone.

Primary cells and cell lines are often cultivated in medium enriched with serum, whose composition is usually unknown, thus removing the possibility to limit metabolite uptake fluxes using known medium composition and metabolite concentrations, as it is possible with defined medium. Additionally, targeted approaches usually quantify a limited, pre-defined set of metabolites. In this study, we addressed these challenges of integrating the extracellular metabolomes of cancer cell lines. Our novel method minExCard predicted a completed metabolome for each sample and enabled us to exclude many metabolite exchanges along with the associated pathways, resulting in individually reduced condition-specific metabolic cell line models (Fig 1). We opted for the minimal set of additional metabolites to ensure that the model was sufficiently constrained, yet satisfied a minimal growth phenotype, given the known metabolite exchange profile and the topology of the metabolic model. Gene expression data confirmed the expression of extracellular enzymes and transport proteins for the added exchange metabolites in the MCF-7 cells (see S1 Text, S1A Table). The completion of the metabolome distinguishes our approach from the frequent application of uptake and secretion rates as constraints in metabolic models, e.g., [23]. A biomass objective function can be assumed for proliferating cancer cells [47]. The quality of the condition-specific metabolic cell line models was assured through the ability of the vast majority of the models to grow at experimental growth rates. Individual models were limited to the experimentally reported growth rates just based on the applied constraints or grew consistent with experimental growth rates when comparing against the median predicted growth rates obtained from the sampling analysis (S1N Table).

A high variation in the published metabolomic data [15] with respect to uptake and secretion profiles for the same cell lines was noted at the beginning of this study, motivating us to create individual models for each replicate. These models yielded also different phenotypes despite belonging to the same cell line (see S1 Text). Variability has been previously observed within clonal cell populations, where noise in gene and protein expression is connected to structural and behavioral differences [48, 49]. Emerging tissue specific patterns could be observed and they were most distinguishable for melanoma cell lines (Fig 4). In particular, we identified phenotypes ranging from highly glycolytic to those almost completely relying on oxidative phosphorylation (Fig 2). Moreover, the energetic classification provided a better distinction of the glycolytic models (Fig 2B and 2C, energetic classification), and the environmental response stratification was better at distinguishing the OxPhos models (Figs 2E and 3, S1 Text). This diversity of metabolic strategies is in agreement with the increasing number of studies that highlight diverse metabolic pathways to play a role in cancer cell proliferation, and that could be targeted during cancer treatment [3, 15]. Moreover, tissue origin and the sequence of environmental factors and oncogenes that occur during the transformation, might influence in which ways the metabolism is altered and gives rise to the different phenotypes [8]; this was reflected in our cancer cell line models (Fig 2). Specifically, hypoxia is believed to drive transformation [8], and tumor cells are exposed to temporal fluctuations in oxygenation [14]. Cells exposed to variable oxygenation would consequently be expected to display metabolic flexibility to withstand fluctuations, whereas cells originating from tumor regions with a constant nutrient supply would not necessarily require such flexibility. Our models were classified into three distinct oxotypes (Figs 3 and 4) that may have arisen from distinct oxygenation conditions during tumor development and progression.

The low oxotype and the glycolytic phenotype was a distinctive feature of the modeled melanoma cell lines. Almost one third of the models depending on a reverse flux through the reductive carboxylation under hypoxic conditions were melanoma models and all melanoma models could carry a non-zero, reverse flux through these reactions (S1M Table). This distinctive feature combined with the ability to deal with hypoxic stress is interesting since skin tissue is at least partially hypoxic [39, 50]. The hypoxic environment of the skin might support the transformation of skin cells [40] and might further promote the glycolytic phenotype in skin tumors. Jain et al. suspected unique metabolic behavior in melanoma cells based on the unique release of adenosine and inosine [15]. However, data clustering did not yield a distinction of the melanoma cell lines from other cancer cell lines [15]. Adenosine secretion, as observed by Jain et al., has been reported under hypoxic conditions [51], supporting the predicted low oxotype, or psydohypoxia [5], as a common feature of the NCI-60 melanoma cell lines.

Using published protein and RNA expression data, the predicted tissue pattern could be validated (Tables 1 and 2). For instance, reductive carboxylation has previously been described as common feature of melanoma cell lines [52, 53]. Differences on the importance of IDH1 as compared to IDH2 have been reported for various cancer cell types [5, 52, 54]. Based on our simulations, a reverse flux through IDH2 was required in some of the melanoma models. In support of this in silico result, IDH2 but not IDH1 expression could be measured on the protein level in normal skin cells and melanoma (Table 1). Additionally, IDH2 downregulation decreased growth, angiogenesis, and increased apoptosis in tumors formed by melanoma cells injected into mice as compared to IDH2 wild-type melanoma cells [55, 56]. However, in this study, the effect of IDH2 downregulation was attributed to diminished mitochondrial NADPH production [55].

In further support of the distinctive tissue patterns of the melanoma cell lines, VHL was not detectable at protein levels in skin cancer or melanoma, and only low RNA expression levels were detected in melanoma cell lines that were not part of the NCI-60 panel (Tables 1 and 2). Lack of VHL, which usually marks HIF-1*α* for proteosomal destruction [57], could promote a pseudo-hypoxic or low ‘oxotype’ and a glycolytic phenotype in melanoma through HIF1*α* stabilization and increased expression of, e.g., the Glut-1 transporter and glycolytic enzymes [39, 40]. VHL is not frequently mutated in skin cancer as compared to, e.g., renal cancers. Nevertheless, the loss of VHL was shown to increase HIF levels and expression of genes with HRE elements, e.g., VEGF, GLUT-1, ALD-A, and PFK-L, which are associated with a glycolytic phenotype, when melanoma cells were injected into mice [58]. Additionally, loss of VHL was connected to increased skin vascularization, which is known to contribute to growth and angiogenesis, potentially through iNOS and EPO signaling, in many cancers including melanoma [58, 59]. Hence, even though VHL is not frequently mutated in melanoma, low expression and lack of protein could promote the glycolytic phenotype we predicted for melanoma.

Taken together, our condition-specific models predicted metabolic commonality among the condition-specific melanoma models. Moreover, the available data supported that the predicted tissue pattern may also be valid for other melanoma cell lines, primary melanoma and other skin cancers.

Metabolic models are often generated from transcriptomic or proteomic data [47, 60], or considering metabolomic data as subordinate source of information. Yet, metabolite concentration changes reveal the consequences of post-transcriptional and post-translational regulation, hence metabolomic data are the closest information source to the metabolic phenotype. In this study, we built condition-specific models that were consistent with the measured uptake and secretion profiles covering a large number of metabolites (n between 95 & 105). As a consequence, these models exhibited a limited flexibility in the uptake and secretion of the measured metabolites, while most unmeasured metabolites were considered to be not exchanged by the cells with the medium. The limited flexibility prevents us to answer the question if cells could in vivo evade the effect of the gene knock out by redirecting the metabolic fluxes in a way that would impact the uptake and secretion pattern. In comparison, an assessment of essential genes in changing environmental conditions can be performed using cell-type specific models which are generated from transcriptomic or proteomic data sets. In an earlier study, Yizhak et al have generated cell-type specific models for the NCI-60 cell lines using transcriptomic data [33]. These models could secrete lactate at the experimentally reported secretion rate, we based our models on. Yizhak et al have also reported an increasing oxygen uptake as a consequence of inhibiting glycolytic enzymes in two lung cancer cell lines [33]. In contrast, our models of the same cell lines predicted a decreasing oxygen uptake when glucose uptake was decreased (Fig I in S1 Text) [33]. This difference in prediction could be explained by the constant lactate secretion flux that we enforced throughout the in silico experiment. Unfortunately, glucose/glutamine uptake was not measured in the experiments of Yizhak et al. [33], which would have enabled a better comparison of the results from these two studies.

If metabolomic and transcriptomic data were individually sufficient one may expect similar models for the cell lines and comparable phenotypic predictions. However, this is not the case and highlights the complementarity and importance of using multiple omics data sets to derive condition- and cell-type specific models to investigate the condition-specific and general cell-type specific metabolic properties. The methods we used to integrate the metabolomic data are readily available and can be used to further investigate the cellular phenotypes [61, 62].

### Conclusion

We opted to build our models solely from metabolomic data, without consideration of the genotypic and other omics data, to evaluate whether such models could provide novel biological insights. This approach was particularly interesting for connecting the melanoma cell lines with the reverse flux through the IDH2 and psydohypoxia [5].

However, when only constrained based on the metabolomic data, our 786-O models predicted net reductive carboxylation was optional, which stood in contrast to net reverse flux observed for these cells. Limiting the oxygen uptake in the models to the minimum emphasized the reverse flux in the TCA cycle. Overall, this highlights that oxygen consumption in an experiment determines the observable metabolic phenotype, in addition to the growth medium composition (or environmental condition). Addition of, e.g., transcriptomic data could further define the phenotype [62]. There is no shortage in transcriptomic data for the NCI-60 cell lines. However, since the models build herein are condition-specific, the data used should originate from the same experiment to resemble the metabolic phenotype displayed in the experiment.

The presented computational modeling approach is applicable to many cellular systems and represents a valuable starting point to investigate metabolic strategies of individual cell lines as well as to envision clinical applications. Further development of this approach could help realize personalized clinical applications utilizing metabolomic data. Immortalized cell lines, such as the NCI-60 cell lines, have a limited clinical relevance since they are monoclonal and accumulate mutations due to the high passage numbers [63]. One way to increase the clinical relevance of our work would be to extend the presented work to omics data generated from patient-derived primary tumor cells. Methods exist to cultivate primary tumor cells, or selected sub-populations of the same, e.g., tumor-initiating stem cells; and to retain phenotype and genotypes using tissue-specific supplements and environmental conditions [63]. Extracellular metabolomic data or multiple omics data derived from such personalized cell cultures could then be used in conjunction with the presented approach to gain a better understanding of an individual’s cancer, and to predict appropriate treatment strategies.

## Materials and methods

The procedures for preparation, data integration, and phenotypic analysis have been summarized in great detail elsewhere [61]. The MetaboTools contains the matlab code used for this work [61].

### The global model

The global model constitutes a subset of Recon 2 [20]. This subset is the same as that used in a previous study [62]. Units (U) are given in fmol/cell/hr. The MetaboTools function *setMediumConstraints* was used to apply the following constraints to the global model [61]. Essentially, infinite constraints were set to lb = −2,000 U and ub = 2,000 U. All exchange reactions in the model were initially set to lb = −2,000 U and ub = 2,000 U. Subsequently, constraints were set for exchange reactions of ions (lb = −100 U), vitamins (lb = −1 U), essential amino acids (lb = −10 U) and compounds such as water or protons (lb = −100 U). Oxygen uptake was constrained to lb = −1,000 U and ub = 0 U. This range was defined based on reported oxygen uptake rates of a cancer cell line (2.85 ⋅ 10^-6^ml O_2_/10^5^cells/min = 646.013 U [64]). Additionally, the lower bounds of the superoxide anion and hydrogen peroxide exchanges (i.e., uptake flux) were set to zero to prevent the generation of models that did not require oxygen uptake.

Reaction fluxes are usually in units of mmol/gDW/hr. Here, however, the metabolite uptake and secretion profiles were mapped in the unit fmol/cell/hr [15]. We assumed a unitary cell weight of 10^-12^ g, which was in the range of the dry weight (3.645 ⋅ 10^-12^ g) that we calculated for lymphocytes in an earlier study [62]. In that study, the dry weight was inferred from the dry mass (range 35–60 ng [65]) and cellular volume (4000 *μ*m^**3**^ [66]) of the human osteosarcoma cell line U2OS, which we related to the cell volume of lymphocytes (243 *μ*m^3^) [67]. By calculating 4000/243 = 16.46, 60 pg/16.46 = 3.645 pg (3.645 ⋅ 10^-12^ g) [62]. According to 1mmol/gdw = 10^12^fmol/10^12^ cells, no biomass scaling was necessary. The lower bound (lb) of the biomass objective function was fixed to a minimal value of 0.008 U to match the lb defined for the slowest growing cell line in the data set (HOP-92, 88 hrs) [27], ensuring that the model building resulted in functional models with non-zero growth. S1Q Table lists the reactions and constraints of the global model.

### The metabolomic data

We used published metabolomic data [15]. There were two quantitative extracellular metabolomic profiles for each of the NCI-60 cell lines. These profiles defined the uptake and secretion rates of 115 metabolites [15]. From the entire set of detected metabolites, we used only the calibrated (quantitative) uptake and secretion fluxes. Fluxes were provided in the unit fmol/cell/hr (U) and were incorporated as such into the model. Throughout the manuscript, fluxes are reported in the unit fmol/cell/hr (U).

### Identification of missing reactions

Metabolite identifiers in the data were mapped to the metabolite abbreviations in the global model. The metabolite aminoisobutyrate was not part of the global model and was excluded. We identified the existing metabolite exchange reactions based on the metabolite abbreviations. If there was no exchange reaction in the model but if the metabolite itself was part of the model, a new exchange reaction was added to the model. In addition to the exchange reactions, transport reactions need to be present in the model to account for transport of metabolites between the extracellular space and the cytosol of the model. Transport reactions need to be added for all metabolites for which we added exchange reactions. These transport reactions were identified from the literature. If no transporter for the metabolite could be identified, we added a diffusion reaction. The additions that we made to the model based on the metabolomic data comprised 44 transport and 37 exchange reactions (S1R Table). The global model used to generate the cancer models comprised 3,935 reactions and 2,833 metabolites.

The Integration of the metabolomic data was performed as detailed in a protocol that provides extensive support (including workflows, code, and tutorials) for the data integration, model generation, and model analysis, carried out in this study [61].

### Constraint-based modeling

Consider the optimization problem

$$\begin{array}{c} \begin{array}{cc} \min & \theta (v)\\ \text{s.t.} & S\cdot v=0,\\ & lb\leq v\leq ub, \end{array} \end{array}$$ (1)

where $v\in R^{n}$ is a vector of reaction rates, *θ*(*v*) is a scalar valued objective function and $S\in R^{m\times n}$ is the stoichiometric matrix consisting of *m* metabolites and *n* reaction rates as defined by the metabolic reconstruction. The lower and upper bounds, *lb* and $ub\in R^{n}$ respectively, constrain the sign and magnitude of the reaction rate, with the convention that a net forward rate is positive. In flux balance analysis (FBA [68]), the objective is to minimize *θ*(*v*): = *c*^*T*^ ⋅ *v*, a linear sum of reaction rates, where c∈R is a parameter vector that specifies the linear contribution of each reaction rate to the objective function. When minimizing a single reaction rate, every entry of *c* is zero, except one. Typically, there is an infinite number of optimal reaction rate vectors that produce an optimal value of the objective function. To obtain a unique flux vector, we first solve Problem (1) with *θ*(*v*): = *c*^*T*^ ⋅ *v*, then fix the rate of the previously optimized reaction and again solve Problem (1) except with $\theta \left(v\right):=\frac{1}{2}v^{T}\cdot v$. This procedure returns a unique reaction rate vector that minimizes the square of the Euclidean norm of the reaction rates, subject to optimality with respect to the original objective function [21]. In flux variability analysis (FVA), one uses linear optimization to compute the minimal and maximal rate of each reaction, subject to *θ*(*v*): = *c*^*T*^ ⋅ *v* being minimal as computed in Problem (1) [34].

### Addition of quantitative constraints

The presence of an exchange and transport reactions does not ensure that a metabolite can be consumed or secreted by the model because anabolic and/or catabolic pathways may not be present or unknown [20]. We used the MetaboTools function *prepIntegrationQuant* to generate individual uptake and secretion profiles for each sample in the data set: To identify the subset of metabolites that the model could consume and secrete, we performed FBA while enforcing small uptake (ub = −0.0001 U) or secretion (lb = 0.0001 U) for all mapped metabolite exchanges. All metabolites that could not be consumed (14) or secreted (14) by the model were discarded (S1S Table). Among them was homoserine 4-hydroxybenzoate, which could be neither consumed nor secreted by the model. Therefore, data for 112 metabolites could be mapped. Note that these 112 metabolites included those that could only be consumed, only be secreted, or by both consumed and secreted (S1S Table). The identification of metabolites that are not part of a metabolic reconstruction is common, and pathways for these metabolites need to be added in future releases of the human metabolic model [20], which served as a starting point (see also above). If the uptake of a metabolite was possible in the global model but secretion was not, only metabolite secretion was discarded from the metabolic profiles, while uptake remained present, and vice versa.

After the sets of ‘qualitatively’ feasible metabolite exchanges were identified, we mapped the sets of metabolite uptake and secretions of a sample to the global model using the MetaboTools function *setQuantConstraints* [61]: We mapped a minimum of 95 and a maximum of 105 exchanges to the models (S1T Table). These exchanges were split into uptake and secretion. The number of metabolite uptakes mapped to the model ranged between 34 and 58, and the number of secretions enforced in the model varied between 42 and 67. We imposed each detected, quantitative flux *x* as a constraint to the bounds of the respective metabolite exchange reaction while considering a 20% allowance around *x* (lb = 0.8*x* U and ub = 1.2*x* U). The constraint pairs for one sample were mapped to the global model one by one. After constraints were placed on one exchange reaction, FBA was performed to check if the model was still able to grow. Although the global model was able to perform all qualitative metabolite exchanges that were mapped, certain quantities or combinations of constraints could still render the model infeasible. In case of infeasibility, the original bounds of the model were restored, and we proceeded to the next set of constraints. Quantitative constraints rendered 27 preliminary cell line models infeasible (Fig 1A). Of these 27 models 25x2, 1x1, and 1x4 exchange constraints were restored during the data integration (S1B Table).

### Model building

Although 464 of the reactions in the global model can exchange metabolites across the boundary of a cell, the exchange of only 115 metabolites was actually quantified in the metabolomic profiles that we employed. The incompleteness of the metabolic profiles results from limits to the scope of individual metabolomic platforms, e.g., oxygen uptake rates that were not reported. This issue was compounded due to the use of fresh medium that was undefined with respect to small molecules, e.g., fresh medium containing serum. In the preliminary model, removing all but the metabolite exchanges (corresponding to measured, exchanged metabolites) always led to a model that did not admit a feasible steady-state flux. We hypothesized that the metabolic profiles were likely to be an incomplete representation of the total number of metabolites exchanged with the medium. Therefore, we developed a novel method, deemed minExCard, that takes a preliminary metabolic model as the input and predicts a steady-state flux vector with the minimum cardinality for the reactions corresponding to the missing exchanges. That is, it predicts a minimal number of missing exchange reactions that are required to be active to permit a feasible steady state flux (S1O Table).

#### minExCard

There is currently no polynomial time algorithm used to find an exact solution to the problem of minimizing the cardinality of a vector (subject to linear constraints). However, subject to certain conditions, the solution to this problem is, with high probability, equivalent to the minimal one-norm solution [69]. Without a loss of generality, we convert all net reactions into a pair of unidirectional reactions and assume that the rate of each unidirectional reaction is non-negative. Let *v*_*e*_ denote a vector of all missing unidirectional exchange reaction rates in the preliminary model, and let |*v*_*e*_| denote the cardinality of the missing exchange reaction vector for a feasible steady-state flux. The linear optimization problem

$$\begin{array}{c} \begin{array}{cc} \min & 1^{T}\cdot v_{e}\\ \text{s.t.} & [\begin{array}{cc} S & S_{e} \end{array}]\cdot [\begin{array}{c} v\\ v_{e} \end{array}]=0\\ & lb\leq v\leq ub\\ & 0\leq v_{e} \end{array} \end{array}$$ (2)

will return the optimal flux vectors *v*^⋆^ and $v_{e}^{⋆}$, such that $\left|v_{e}^{⋆}\right|$ approximates the smallest possible cardinality of any *v*_*e*_ that satisfies the constraints. For the preliminary model, the number of linearly independent equality constraints, i.e., rank([*S* *S*_*e*_]) = 2746. Of the 120 preliminary models, the largest number of active inequality constraints on non-exchange reactions was 39. This leaves a maximum of 2707 = 2746 − 39 independent constraints, which is an order of magnitude greater than 28, which was previously the largest $\left|v_{e}^{⋆}\right|$ obtained for any of the preliminary models. A high ratio of the number of independent constraints to the cardinality of the linear optimization solution is used in similar settings to guarantee, with high probability, that a one-norm approximation recovers the actual minimal cardinality solution [69]. To ensure that the elimination of any one reaction where *v*_*e*_ ≠ 0 would result in Problem (2) becoming infeasible, we conducted FVA on the exchange reactions and repeated minExCard if needed. This procedure for minimizing the cardinality of missing exchanges was applied to all 120 models. Thereafter, the sets of unused exchange reactions, specific to each model were omitted. Finally, all other net, non-exchange reactions that did not admit a non-zero flux were omitted via the application of a previously reported function (identifyBlockedRxns, epsilon = 1e^**-4**^) within the FASTCORE software package for the reconstruction of context-specific metabolic networks [70]. The number of exchanges added to the model due to minExCard varied between 13 and 28 (S1B and S1U Table). The minExCard is embedded in the MetaboTools function *setQuantConstraints* or can be called directly by the function *generateCompactExchMode* [61].

### Analysis

#### Growth rates

A minimal biomass growth requirement (minGrowth = 0.008) was specified as the input for the model generation using *setQuantConstraints* and resided in the models throughout the analysis. Cell line specific growth rates [27], which agreed with [15], were added as constraints only to analyze the ability of the models to realize experimental growth rates. An alternative set of NCI-60 growth rates (http://dtp.nci.nih.gov/docs/misc/common_files/cell_list.html) did not yield different results.

#### Flux split ratios and ATP yield

Flux splits can be used to investigate metabolism in a metabolite-centric view [71]. Flux split analysis identifies the contribution of each model reaction producing ATP to the total amount of ATP produced [71]. Herein, we calculated flux splits to obtain information on the distinct production strategies of the cancer cell line models for ATP and cofactors (NADH, NADPH, and FADH2). The MetaboTool function *predictFluxSplits* was used [61]: The flux splits were calculated based on the flux vectors identified through optimizing ATP production for each model (parsimonious FBA). The advantage of using parsimonious FBA over “normal” FBA is that it provides a unique solution, whereas “normal” FBA returns one of many alternate optimal solutions, which may differ depending on the chosen solver and linear programming method. In parsimonious FBA the objective is maximized while minimizing the the euclidean norm of internal reaction fluxes (and hence the flux through loop reactions (S1V Table) and considering the applied uptake and secretion flux constraints. All reaction fluxes producing the metabolite *i* were identified: *P*_*i,j*_ = *S*_*i,j*_ × *v*_*j*_ for all reactions *j* as *P*_*i,j*_ > 0. From the sum of production fluxes *Φ*_*i*_ = ∑*P*_*i,j*_, the percent contributions were calculated: *P*_i_* = *P*_*i,j*_/*Φ*_*i*_ as specified [71]. However, prior to summarizing the total production flux *Φ*_*i*_, certain reactions, e.g., transport reactions, were removed. Subsequently, the reaction with the maximal *P*_i_* was identified as the major producer of ATP, NADH, NADPH, and FADH2. Based on the combination of major producer reactions, the 120 models were classified into eight different phenotypes (S1J Table). The ATP yield was defined by dividing the *Φ*_*ATP*_ by the glucose uptake of each respective model. It should be noted that although we formulated the ATP yield according to glucose uptake, the uptake of other carbon sources, e.g., glutamine, remained possible, as no additional constraints were applied. The MetaboTools function *make3Dplot* can be used to generate similar illustrations as presented herein [61].

#### Phenotypic phase plane analysis

The robustness of the 120 models towards environmental perturbations was investigated using phenotypic phase plane analysis (PhPP) [31]. The MetaboTools functions *performPPP* and *illustrate_ppp* were used to perform this step: Fluxes through two exchange reactions representing metabolite uptake or secretion were fixed at different intervals while setting biomass production as the objective function in FBA. For each step, the optimal value was computed and plotted in 3D. Oxygen uptake was varied in combination with either glucose uptake, glutamine uptake, or lactate secretion. All other reaction constraints remained unchanged. The tested range was defined based on the variability of the constraints set throughout the set of 120 models: oxygen uptake rate was initially 0 and decreased in steps of 20 units until an uptake rate of −1,000 U was reached. The glucose uptake rate was initially 0 and decreased in steps of 20 units to −1,080 U (the lowest and highest glucose uptake rates among the models were −38 x 0.8 = −30 U and −860 x 1.2 = −1,032 U). The glutamine uptake rate was initially 0 U and decreased in steps of 20 units to −400 U (the lowest and highest glutamine uptake among the models were −13.87 U x 0.8 = −11.096 U and −304.27 U x 1.2 = −365.124 U among the models). The lactate secretion rate was initially 1,620 U and decreased in steps of 20 units to 0 U (the lowest and highest lactate secretion rates were 32.35 U x 0.8 = 25.880 U and 1,345.14 U x 1.2 = 1,614.2 U). The distinction of phenotypes was based on the shape of the solution spaces predicted by the models (Figs G and I in S1 Text).

#### Gene deletion

We performed single gene deletion, using the function *singleGeneDeletion* and the *uniqueGene* option of the COBRA toolbox for each of the 120 models [21]. The MetaboTool function *analyzeSingleGeneDeletion* was used to predict and summarize the results of the gene deletion analysis [61], which relies on the COBRA toolbox function function *singleGeneDeletion* and the *uniqueGene* option. Growth termination was defined as growth rate < 5%. Reduced growth was defined as growth rate > 5% and < 95% of the maximal achievable growth rate of the model.

#### Analysis of protein abundance and RNA expression data

We analyzed cancer cell line, tumor, and normal tissue for protein and RNA expression levels of IDH1, IDH2, HIF1*α*, and VHL using data from the Human Protein Atlas version 15 Ensembl version 78.38 [37]. The hypergeometric probability for the observed RNA or protein levels was defined using R (phyper, http://www.R-project.org/). The data from the human protein Atlas provided protein levels for 20 human tumor tissues including “skin cancer” and “renal cancer” [37]. From the 48 normal tissue protein expression data we analyzed the tissues “skin 1” and “skin 2”. RNA-seq data of tumors included 44 cell lines and 32 tissues. RNA expression was analyzed for all skin cancer cell lines (“A-431”, “SK-MEL-30”, “HaCaT”, “WM-115”) and all NCI-60 cell lines (“A549”, “K-562”, “HL-60”, “MOLT-4”, “PC-3”, “RPMI-8226”, “MCF-7”, and “U-251 MG”). We added the tissue-specific RNA expression data for “skin” and “kidney” to the table (Table 2).

#### Identify mutations

cBioPortal was queried for mutations in the NCI-60 cell lines (http://www.cbioportal.org), [41].

#### Sampling analysis

The sampling analysis was performed using the MetaboTools functions *performSampling* [61]. Parameters were chosen as follows nFiles = 100; pointsPerFile = 5000; stepsPerPoint = 2500; maxTime = 3600000; fileBaseNo = 1; warmupn = 10000.

All simulations were performed in MATLAB (MathWorks, Inc.) using the Tomlab (Tomlab, Inc.) linear programming solver.

## Supporting information

S1 Table A) Statistics overview. B) Experimental growth rates. C) Sampling results. D) Sanity check of ATP production from Glucose only. E) Flux Split for ATP. F) Flux Split for NADH. G) Flux Split for NADPH. H) Flux Split for FADH2. I) Respective affiliation to phenotypes based on energy and cofator production strategies. J) Visual stratification of the solution space form and size. K) Genes essential in all models. L) Lowering the oxygen uptake rate (lb = −100 U) increased the number of models that had to use RC. M) FVA results of minimal oxygen uptake. N) Constraints of the global model and cancer cell lines models. O) Reactions added to the global model. P) Excluded metabolites. Q) Number of mapped uptakes and secretions per model. R) Added exchanges by metabolites. S) Reactions discarded from flux split analysis (and ATP yield).
(XLS)

S1 Text Fig A. Clustering of data. Fig B. Coverage of 38 subsystems varied among the 120 models. Fig C. Considering both ATP producing glycolysis reactions. Fig D. Distinction of glycolytic and OxPhos models. Fig E. ATP yield is not informative for the division of OxPhos models. Fig F. Classification of the phenotypes of the phenotypic phase planes. Fig G. Additional 2D plots for Fig 2 of the main manuscript. Fig H. ATP yield does not correlate with the maximal growth rate of the models. Fig I. Phase planes of the 120 models.
(PDF)
