# Supplementary material for: A systems approach reveals distinct metabolic strategies among the NCI-60 cancer cell lines
Source: PLoS Comput Biol. 2017 Aug 14;13(8):e1005698. doi: 10.1371/journal.pcbi.1005698 (PMC5570491; doi:10.1371/journal.pcbi.1005698)
Supplement: S1 Text — Fig A. Clustering of data. Fig B. Coverage of 38 subsystems varied among the 120 models. Fig C. Considering both ATP producing glycolysis reactions. Fig D. Distinction of glycolytic and OxPhos models. Fig E. ATP yield is not informative for the division of OxPhos models. Fig F. Classification of the phenotypes of the phenotypic phase planes. Fig G. Additional 2D plots for Fig 2 of the main manuscript. Fig H. ATP yield does not correlate with the maximal growth rate of the models. Fig I. Phase planes of the 120 models. (PDF) [file pcbi.1005698.s002.pdf]

A systems approach reveals distinct metabolic strategies among the NCI-60 cancer cell lines

Maike K. Aurich[1], Ronan M. T. Fleming[1], Ines Thiele[1*]

[1] Luxembourg Center for Systems Biomedicine, University of Luxembourg, Esch-Sur-Alzette, Luxembourg

∗ E-mail: Corresponding Ines Thiele, ines.thiele@uni.lu

Supplementary Material

- Additional exchanges
- Gene expression connected to additional exchanges
- Most models can grow at experimental growth rates
- Replicate models were more similar regarding ATP production than growth
- Glycolysis, TCA cycle and ETC together were the primary sources of ATP
- Distinction of the clusters derived from a phase plane analysis
- Further discussion of the four models with reductive TCA cycle flux
- Comparison of the predicted phenotypes

Supplementary Figures

- Figure A Clustering of data.
- Figure B Coverage of 38 subsystems varied among the 120 models.
- Figure C Considering both ATP producing glycolysis reactions.
- Figure D Distinction of glycolytic and OxPhos models.
- Figure E ATP yield is not informative for the division of OxPhos models.
- Figure F Classification of the phenotypes of the phenotypic phase planes.
- Figure G Additional 2D plots for Figure 2 of the main manuscript.
- Figure H ATP yield does not correlate with the maximal growth rate of the models.
- Figure I Phase planes of the 120 models.

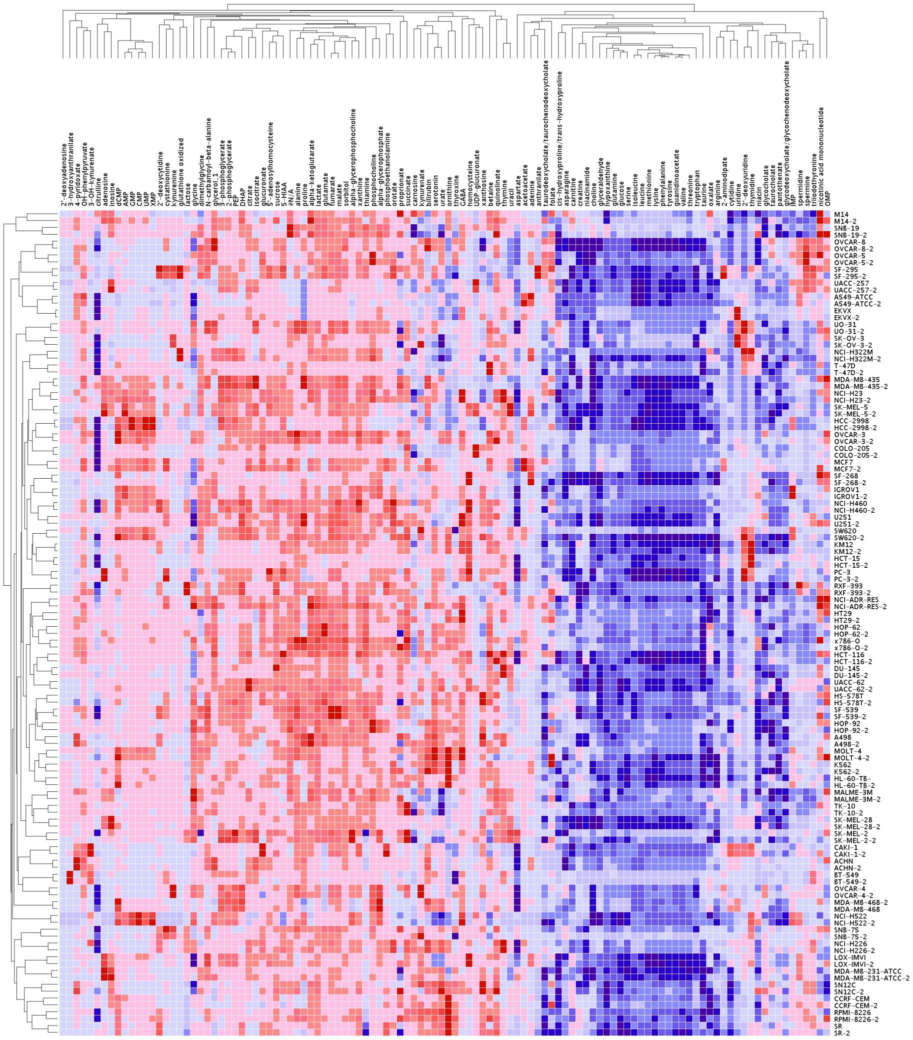

Figure A. Flux data mapped to the reconstruction shows variation between replicates despite the samples clustering together.

In total, 112 metabolites were mapped to our model. For those metabolites, we performed Spearman's rank clustering after the normalization of each column for both the metabolites and cell lines (Fig. A in S1 Text). Although the samples of the model cluster together, there are differences between the samples, e.g., for the two samples of M14, there are a number of metabolites that differ in whether they were consumed (blue) or released (red). Based on these discrepancies, we decided to build a model for each sample rather than combine the replicates and build a model for each cell line.

Glycolysis has two steps that produce ATP; this was neglected when identifying the reaction with the highest ATP contribution. Thus, we assessed the impact of summation on the contribution of the two reactions on the ranked ATP yield plot and classified them into glycolytic and OxPhos phenotypes. As a result, one OxPhos model (NCI-H522) converted into a glycolytic models when considering flux in both ATP-producing reactions in glycolysis (PGK and pyruvate kinase) against ATP production in the electron transport chain (ETC) (Fig. C in S1 Text).

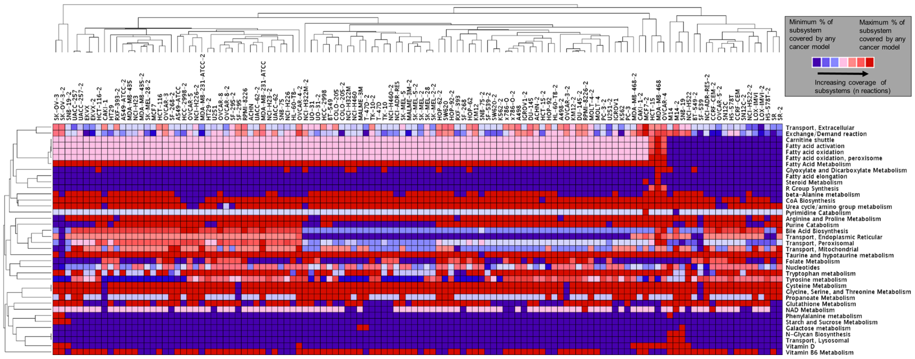

Figure B. Coverage of 38 subsystems varied among the 120 models.

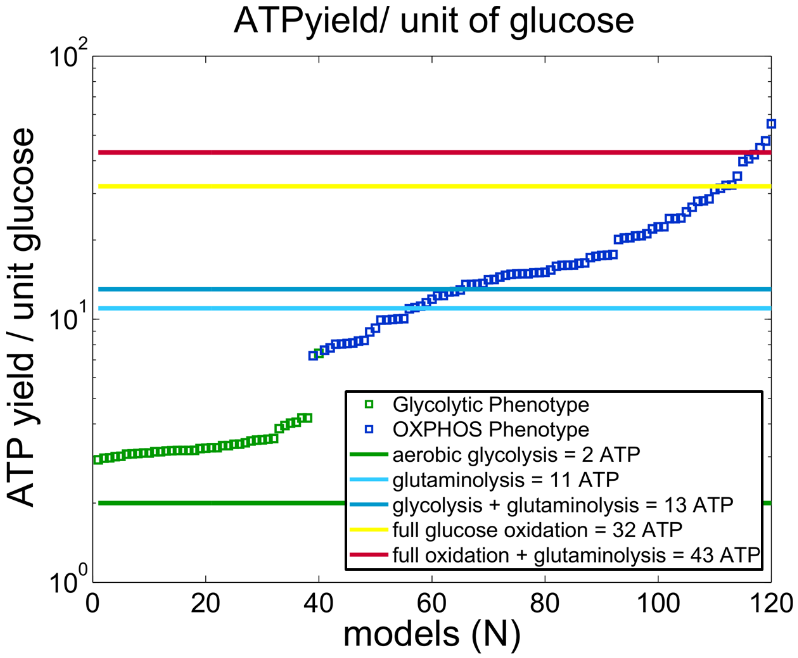

Figure C. Metabolic strategies considering both ATP-producing glycolysis reactions. One model switch to a higher collective contribution of glycolysis compared with the ATP synthetase contribution to total ATP-producing flux.

Additional exchanges

The number of exchanges added to maintain functional models varied between 13 and 28 (Table B in S1 Tables). Each unique set of additional metabolite exchanges (n = 54) was each added in at least one model, and two metabolites were added to all 120 models (Table U in S1 Tables), i.e., O_2, which was only uptaken, and bilirubin-glucuronoside (bilglcur), which had to be either secreted or consumed in all 120 models. The pattern of uptake (n = 76) and secretion (n = 44) of bilglcur was opposite from the exchange profiles of bilirubin, which was subject to uptake in 44 samples and secreted in 76 samples (Table U in S1 Tables). The next most frequently added exchange was 2-hydroxybutanoic acid, which had to be secreted by 116 models. Interestingly, 2-hydroxybutanoic acid has been suggested to be a

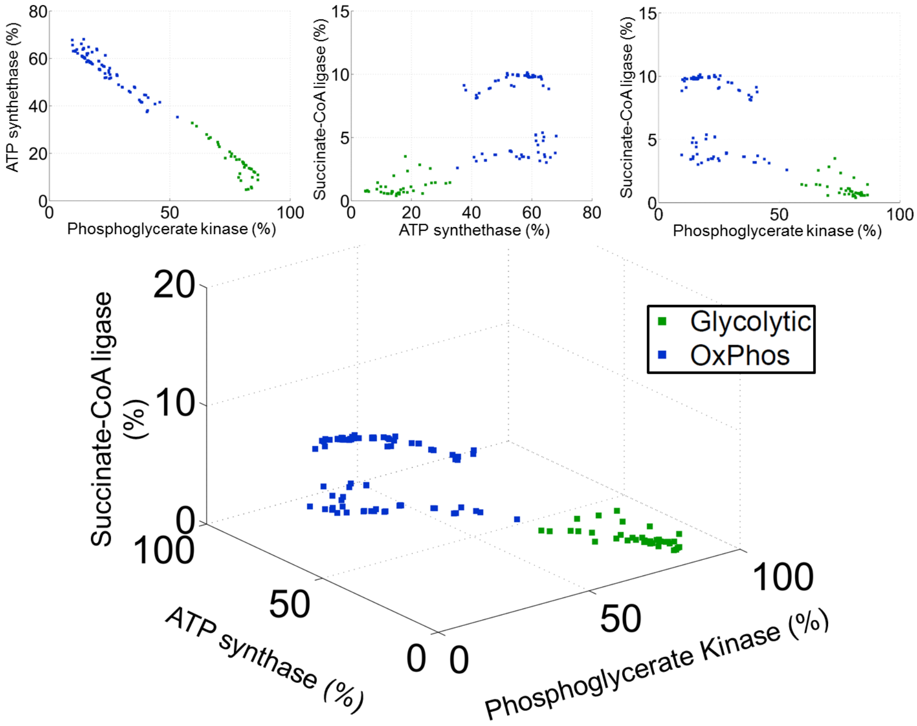

Figure D. Distinction of glycolytic and OxPhos models.

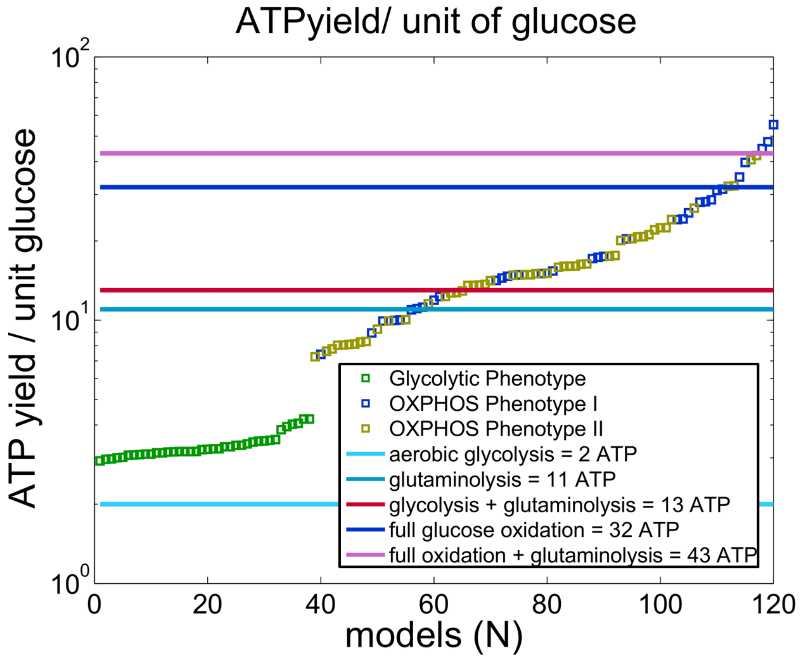

Figure E. ATP yield is not informative for the division of OxPhos models (blue and green).

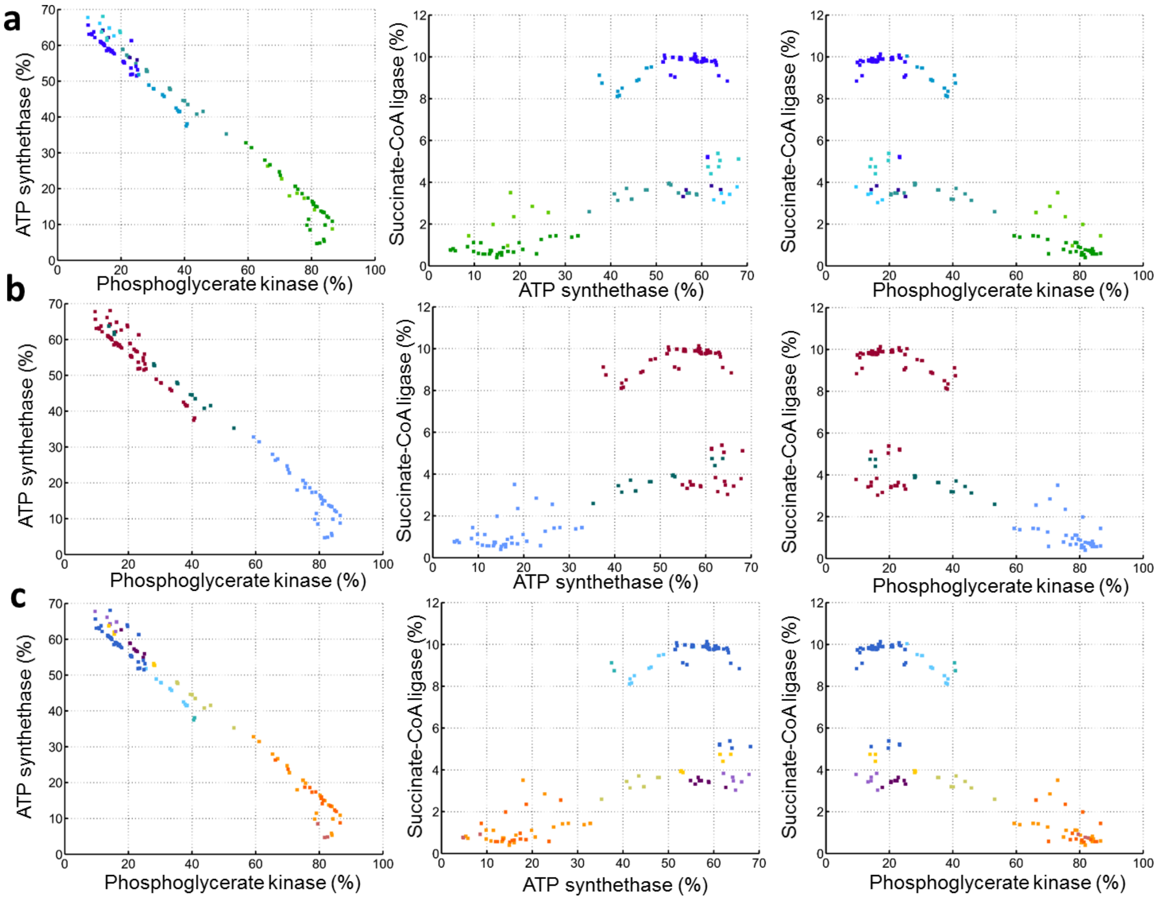

Figure F. Additional 2D plots for Figure 2 of the main manuscript. **a.** A fine-grained division of the OxPhos models was achieved considering production strategies of NADPH, NADH and FADH2 production. These clusters were plotted according to their the contributions of phosphoglycerate kinase, ATP synthase, and succinate-CoA ligase. **b.** Phenotypic phase plane analysis revealed distinct phenotypes with regard to oxygen requirements among the cell line models. These phenotypes were plotted according to their the contributions of phosphoglycerate kinase, ATP synthase, and succinate-CoA ligase. Distinction between the OxPhos models (red and green) was different compared to the phenotypic classification performed based on energy and cofactor production strategies. **c.** Six model clusters were distinguished according to the models robustness towards environmental changes. These clusters were plotted according to their the contributions of phosphoglycerate kinase, ATP synthase, and succinate-CoA ligase.

biomarker for the detection of colorectal cancer via a multiple logistic regression model [1]. The additions further contain a number of fatty acid uptakes that had to be added, e.g., phytanic acid (n = 101, Table U in S1 Tables). Dietary branched-chain lipids, such as phytanic acid, have been linked to various cancers as well as neurological diseases [2, 3]. The intake of phytanic acid or phytanic acid-containing foods has been connected to an increased risk for follicular lymphoma, small lymphocytic lymphoma/chronic lymphocytic leukemia, and non-Hodgkins lymphoma [4]. The plasma phytanic acid concentration was significantly associated with dairy fat intake; however, no direct causal relationship could be established with prostate cancer [3, 5]. Prostate as well as other cancers overexpress alphamethylacyl-CoA racemase

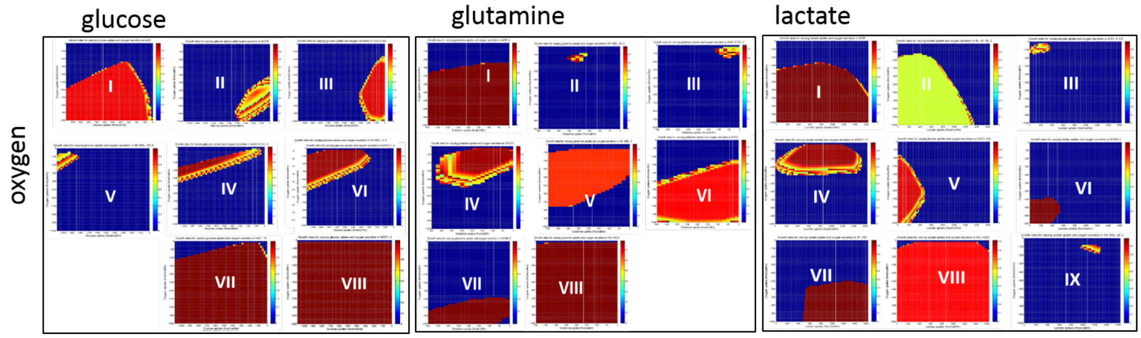

Figure G. Phase plane analysis reveals distinct solution spaces for the variation of nutrients and oxygen between the NCI-60 models. This distinction was performed through visual inspection; however, the transitions between the depicted examples were rather fluid.

(AMACR), an enzyme that regulates the entrance of branched-chain fatty acids into peroxisomal alpha- and beta-oxidation [2, 6]. Furthermore, alternative splicing produces a distinct transcript of AMACR with distinct biochemical properties, and single-nucleotide polymorphisms (SNPs) have been observed to be elevated in prostate cancer compared with normal tissue [6–8].

Gene expression connected to additional exchanges

We have previously combined transcriptomic and metabolomic data, but in comparison to the current case, the data was generated from the same experiment [9]. The combination of multiple omics data sets could provide additional certainty about the predicted exchanges and phenotypes, if these data were generated from the same cells and experiment. The code we used in the previous study is available [10].

To demonstrate that the added exchanges predicted by the model could be supported, we analyzed gene expression data. We downloaded Gene Expression data (http://discover.nci.nih.gov/cellminer/, [11]) for 59 cell lines (missing MDA-N), and processed the data using R, and the packages gcRMA (Wu J and Rafael Irizarry (2016). R package version 2.44.0.) and PANP (Warren P (2016). panp: Presence-Absence Calls from Negative Strand Matching Probesets. R package version 1.42.0.) to distinguish the expression of genes associated with reactions that involve the added, exchanged metabolites (in the extracellular environment). We limited the search for expressed genes to the extracellular reactions and transport reactions because we were mainly interested in the transport reactions that would mediate uptake and secretion of the added metabolites.

The analysis required multiple summary steps: (1.) We summarized the exchanges added to the two models into one list for each cell line. For each metabolite on the list we identified the reactions and from those the set of associated genes. We excluded the metabolite h(e), since it is involved in so many reactions that it would not be likely to not find a reaction associated with an expressed gene. (2.) The absent and present calls were obtained for each probe set. We summarized all probe sets into one call per gene. We assigned a present call if one of the probe sets of a gene was expressed ($p < 0.05$). The data was provided in triplicates and for 59 cell lines. If a gene was called present in any replicate, we called the gene present.

We chose MCF-7 for this exemplified analysis because it had the largest number of added metabolite exchange reactions (30). The largest group of metabolites added were only associated with reactions that were not associated with a gene (15) and hence, could not have been excluded based on gene expression data. Another 14 metabolites were associated with reactions that were associated with at least one gene that was called present. We only report one present gene (Table A in S1 Tables). Finally, there was one metabolite associated with reactions for which we found genes unexpressed. However, to demonstrate that

this might be a condition-specific observation, we queried the human protein atlas and could confirm the presence of SLC16A10 RNA (FPKM:3) in MCF-7 cells (http://www.proteinatlas.org/ENSG00000112394-SLC16A10/cell). Taken together, this analysis highlights that even though gene expression data could provide insight into the validity of metabolite exchanges added to the model, absence of gene expression in one data set does not mean it could not be expressed in a different condition.

Most models can grow at experimental growth rates

Cancer cell lines are known to be heterogeneous [12–15]. One distinctive feature lies in the variability of the doubling times of individual cell lines [16,17]. Large variations in minimal and maximal achievable growth rates were observed across models (Table B in S1 Tables). These differences were the consequence of the imposed quantitative constraints that the models had to address. However, the heterogeneity with respect to growth rates indicated that the quantitative metabolomic differences had been successfully translated into distinct solution spaces of the generated models [18]. Two models did not achieve the *in vivo* growth rates, whereas the remaining 12 models exceeded experimentally reported growth rates under the enforced quantitative constraints. For example, the SK-MEL-28-2 achieved a minimal growth rate (0.034 fmol/cell/hr, 20.5 hr) that exceeded the experimental measurement (bound ub = 0.023 fmol/cell/hr, 35.1 hr) (Table A in S1 Tables). The *in silico* growth in the ACHN-2 model agreed particularly well with the experimental growth (*in silico*: max = 0.0206 fmol/cell/hr, min = 0.008 fmol/cell/hr versus experimental: lb = 0.018 fmol/cell/hr, ub = 0.027 fmol/cell/hr). Additionally, the UACC-257 model was limited to the experimental growth rate +/-20% (growth rate max = 0.0155, min = 0.008; lb = 0.013 ub = 0.016). The ACHN-2 and UACC-257 models were good examples of how the model specifically predicted experimental growth rates as a consequence of the applied metabolomic constraints.

Replicate models were more similar in ATP production than growth

The growth rates of models of the same cell line could be very different (Table B in S1 Tables). We found no correlation (anti-correlation) between the ATP yield achieved by the models and the maximal in silico growth, as would be expected as the two are competing objectives (Fig. H in S1 Text). We compared the similarity of the replicate models with regard to the maximum growth rate predicted for each model. Ordering the models accordingly, we sorted eight pairs of cell line models in direct consecutive order. Similarly, using the ATP yield per unit of glucose, 16 models appeared in a consecutive order. ATP yield was therefore the stronger binding factor for models generated from duplicate samples.

Glycolysis, TCA cycle and ETC together were the primary sources of ATP

Cells can produce ATP in many ways. A number of reactions were identified to contribute to the total amount of ATP produced (Table F in S1 Tables). Among those reactions, the reactions in glycolysis, the TCA cycle and ATP synthethase were the primary ATP producers in each of the models. Glycolysis contributed between 9.5% and 86.6% of total ATP production per model. Similarly, the contribution of ATP synthethase varied between 4.7% and 68.1%. The combined contribution of glycolysis and ATP synthase ranged from 72.9% and 97.5%. Including the contribution of succinate-CoA ligase (0.4%-10.1% of the total ATP production), the three pathways contributed 81.1% to 98.1% of the total amount of ATP per model. Although the combined contribution of glycolysis, TCA cycle and ATP synthase was very high in all of the models, the fraction contributed by either glycolysis or ATP synthase could be very different.

Distinction of the clusters derived from phase plane analysis

Most but not all cancer cells depend on exogenous sources of glutamine for nucleotide and hexosamine biosynthesis [19], while other cancer cells depend on a constant glucose supply [12]. Accordingly, we

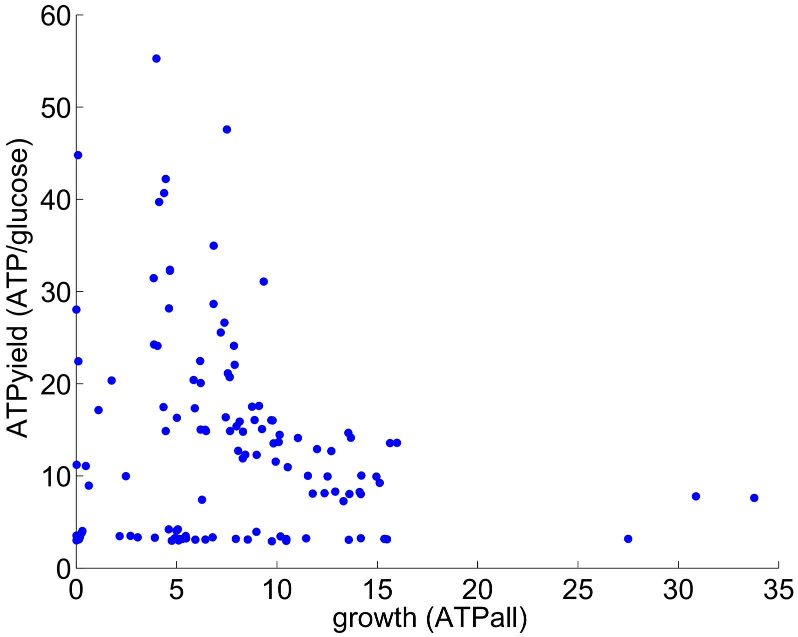

Figure H. ATP yield does not correlate with the maximal model growth rate.

classified the models into distinct clusters based on their dependencies regarding the uptake of glucose, glutamine, and oxygen, as well as lactate secretion (Fig. J in S1 Text, Table K in S1 Tables). Cluster 1 (n = 56) was characterized by the requirement for high oxygen uptake and no dependency upon glutamine uptake or lactate secretion; however, there were subtypes for the models of this cluster. Cluster 1B could only grow when lactate secretion was limited, which coincided with the low utilization of succinate-CoA ligase by these models. Cluster IC was characterized by high glucose and high lactate secretion (SF-295 models). Accordingly, these two models were shifted the furthest towards the glycolytic phenotype (Fig.2e, Fig. Fc in S1 Text). Cluster 2 (n = 7) was characterized by high oxygen uptake, low glucose uptake and lactate secretion, as well as indifference to glutamine uptake. Cluster 3 (n = 6) showed similar characteristics but with a larger overall solution space compared with cluster 2. Cluster 4 models could use oxygen only to a limited extent. Three subclusters were distinguished: cluster 4A was characterized by increasing oxygen requirements as a consequence of increasing glucose uptake. This increase was also indicated in cluster 4B; however, the models belonging to this subcluster had a very restricted solution space and operated only at very high glucose uptake and lactate secretion rates. In comparison, cluster 4A was limited to low lactate secretion rates. Both subclusters were only able to grow with relatively low glutamine uptake. Cluster 4 consisted of all glycolytic models; however, within the subtypes of cluster 4, (the glycolytic models), subtype I (as defined via flux split analysis) spread across clusters 4A and 4C (Fig.2e, Fig. Fc in S1 Text) such that (based on this analysis) these two glycolytic subtypes could not be distinguished. The division into subtypes 4A-C did not describe distinct clusters in the 3D visualization or, in other words, with regard to PGK, ATP synthetase or succinate-CoA ligase utilization by those models (Fig.2e, Fig. Fc in S1 Text). We observed an accumulation of melanoma models in cluster 4. Not all of the melanoma models were part of cluster 4. Two melanoma models were, individually, associated with clusters 5 and 6 (Table K in S1 Tables).

The models of cluster 5 (n = 6) and 6 (n = 8) were independent from oxygen uptake (provided uptake > 0 fmol/cell/h). Furthermore, the models of these clusters widely demonstrated unlimited glutamine or glucose uptake. Cluster 5 was distinguished from cluster 6 by limited lactate secretion ability.

Further discussion of the four models with reductive TCA cycle flux

The four models with reductive TCA cycle flux were all glycolytic models, and all but MALME-3M-2 belonged to glycolytic subtypes (Table J in S1 Tables). Additionally, the four models belonged to cluster 4 in the phase plane analysis (K in S1 Tables). The cluster 4 models could use oxygen only to a limited extent. Cluster 4A was characterized by increased oxygen requirements as a consequence of increased glucose uptake. This increase was also indicated in cluster 4B; however, the models belonging to this subcluster had a very restricted solution space and operated only at very high glucose uptake and lactate secretion rates. In comparison, cluster 4A was limited to low lactate secretion rates. Both subclusters were only able to grow with relatively low glutamine uptake.

Comparison of the predicted phenotypes

We demonstrated the stratification of the cancer cell line models based on three different aspects of the cancer cell line models. Each individual analysis distinguished the models differently, which is also reflected by the different numbers of phenotypes the models were assigned to. The majority of cell line model pairs were associated into the same phenotype by all three different classifications. Classification 1 was based on the prediction of pathways for energy and cofactor production used primarily by the individual model (Fig. 2B-C, Table K in S1 Tables). It assigned 43/60 cell line model pairs into the same of eight phenotypes. Based on the response of the individual models to the modulation of the exchange of glucose, glutamine, oxygen, and lactate, with the environment 44/60 cell line model pairs were assigned to the same (of 6) phenotypes (Fig. 2E, Fig. 3, Table K in S1 Tables, S1 Text). Finally, 49/60 cell line model pairs were associated with the same of the three oxotypes (classification 3, Fig. 4). The cell line pairs associated to the same phenotypes in the first two classifications were subsets of cell line model pairs assigned to the same oxotype. Thus, classification strategies, despite addressing different metabolic features of the models, predicted the cell line model pairs into the same phenotypes.

Figure I: Phase plane analysis for individual models.

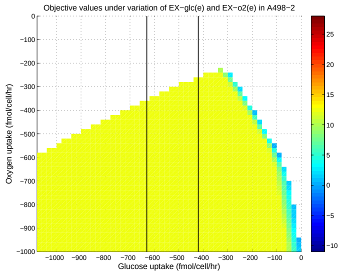
Objective values under variation of EX−glc(e) and EX−o2(e) in A498−2
Oxygen uptake (fmol/cell/hr)
Glucose uptake (fmol/cell/hr)

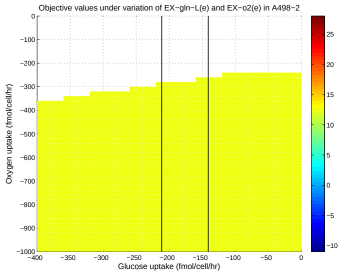
Objective values under variation of EX−gln−L(e) and EX−o2(e) in A498−2
Oxygen uptake (fmol/cell/hr)
Glucose uptake (fmol/cell/hr)

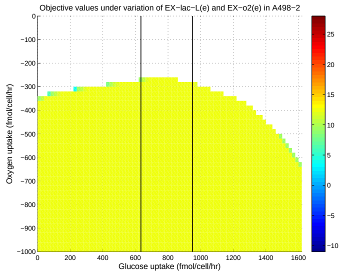
Objective values under variation of EX−lac−L(e) and EX−o2(e) in A498−2
Oxygen uptake (fmol/cell/hr)
Glucose uptake (fmol/cell/hr)

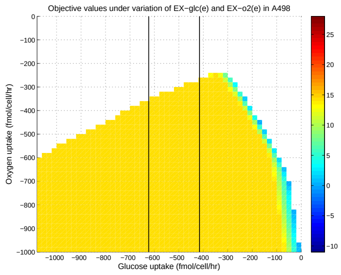
Objective values under variation of EX−glc(e) and EX−o2(e) in A498
Oxygen uptake (fmol/cell/hr)
Glucose uptake (fmol/cell/hr)

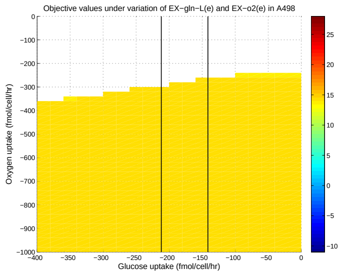
Objective values under variation of EX−gln−L(e) and EX−o2(e) in A498
Oxygen uptake (fmol/cell/hr)
Glucose uptake (fmol/cell/hr)

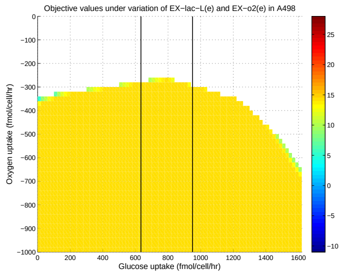
Objective values under variation of EX−lac−L(e) and EX−o2(e) in A498
Oxygen uptake (fmol/cell/hr)
Glucose uptake (fmol/cell/hr)

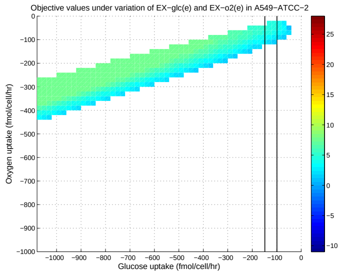
Objective values under variation of EX−glc(e) and EX−o2(e) in A549−ATCC−2
Oxygen uptake (fmol/cell/hr)
Glucose uptake (fmol/cell/hr)

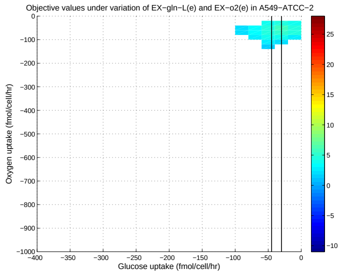
Objective values under variation of EX−gln−L(e) and EX−o2(e) in A549−ATCC−2
Oxygen uptake (fmol/cell/hr)
Glucose uptake (fmol/cell/hr)

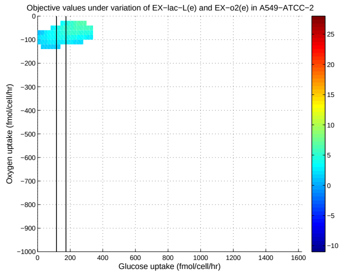
Objective values under variation of EX−lac−L(e) and EX−o2(e) in A549−ATCC−2
Oxygen uptake (fmol/cell/hr)
Glucose uptake (fmol/cell/hr)

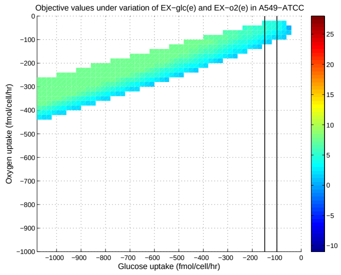
Objective values under variation of EX−glc(e) and EX−o2(e) in A549−ATCC
Oxygen uptake (fmol/cell/hr)
Glucose uptake (fmol/cell/hr)

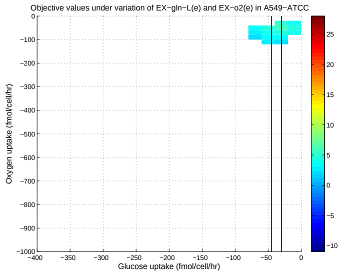
Objective values under variation of EX−gln−L(e) and EX−o2(e) in A549−ATCC
Oxygen uptake (fmol/cell/hr)
Glucose uptake (fmol/cell/hr)

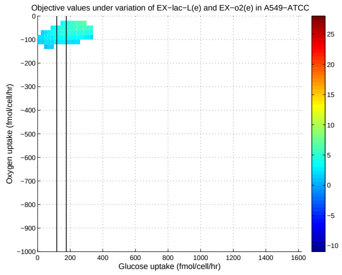
Objective values under variation of EX−lac−L(e) and EX−o2(e) in A549−ATCC
Oxygen uptake (fmol/cell/hr)
Glucose uptake (fmol/cell/hr)

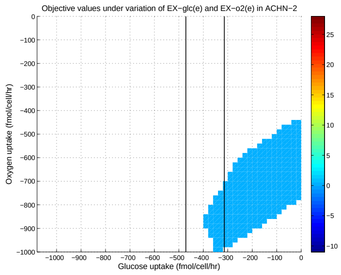
Objective values under variation of EX−glc(e) and EX−o2(e) in ACHN−2
Oxygen uptake (fmol/cell/hr)
Glucose uptake (fmol/cell/hr)

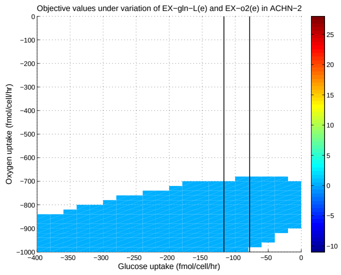
Objective values under variation of EX−gln−L(e) and EX−o2(e) in ACHN−2
Oxygen uptake (fmol/cell/hr)
Glucose uptake (fmol/cell/hr)

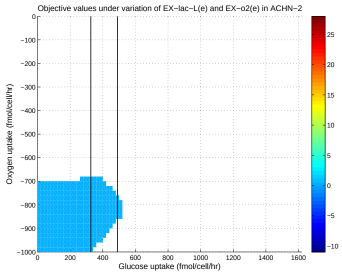
Objective values under variation of EX−lac−L(e) and EX−o2(e) in ACHN−2
Oxygen uptake (fmol/cell/hr)
Glucose uptake (fmol/cell/hr)

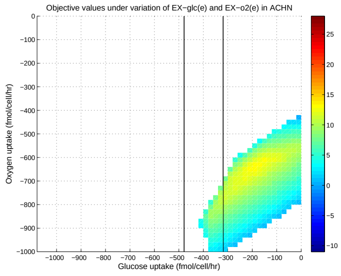
Objective values under variation of EX−glc(e) and EX−o2(e) in ACHN
Oxygen uptake (fmol/cell/hr)
Glucose uptake (fmol/cell/hr)

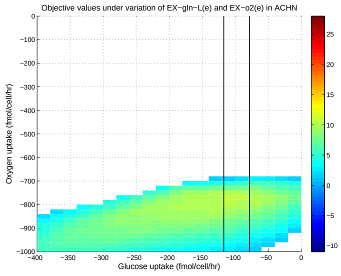
Objective values under variation of EX−gln−L(e) and EX−o2(e) in ACHN
Oxygen uptake (fmol/cell/hr)
Glucose uptake (fmol/cell/hr)

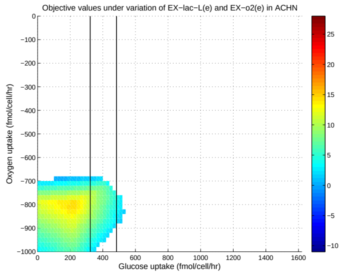
Objective values under variation of EX−lac−L(e) and EX−o2(e) in ACHN
Oxygen uptake (fmol/cell/hr)
Glucose uptake (fmol/cell/hr)

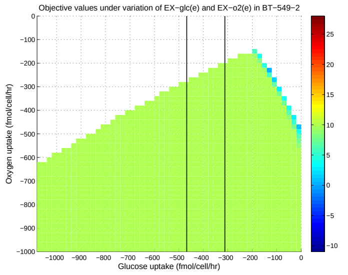
Objective values under variation of EX−glc(e) and EX−o2(e) in BT−549−2
Oxygen uptake (fmol/cell/hr)
Glucose uptake (fmol/cell/hr)

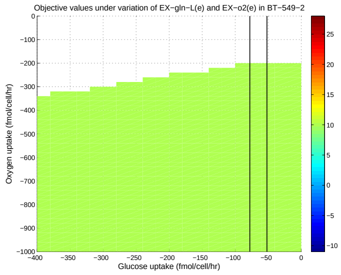
Objective values under variation of EX−gln−L(e) and EX−o2(e) in BT−549−2
Oxygen uptake (fmol/cell/hr)
Glucose uptake (fmol/cell/hr)

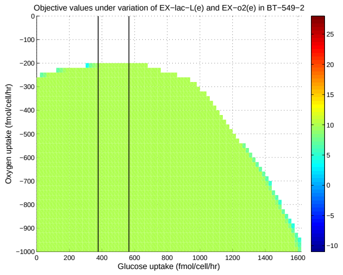
Objective values under variation of EX−lac−L(e) and EX−o2(e) in BT−549−2
Oxygen uptake (fmol/cell/hr)
Glucose uptake (fmol/cell/hr)

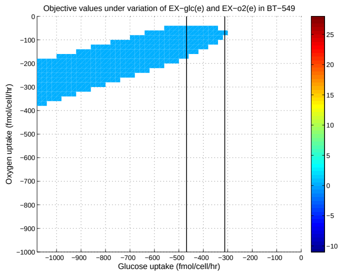
Objective values under variation of EX−glc(e) and EX−o2(e) in BT−549
Oxygen uptake (fmol/cell/hr)
Glucose uptake (fmol/cell/hr)

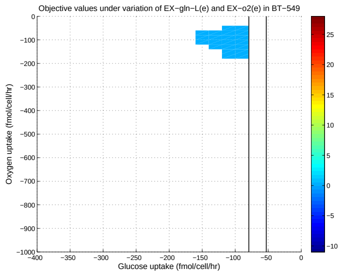
Objective values under variation of EX−gln−L(e) and EX−o2(e) in BT−549
Oxygen uptake (fmol/cell/hr)
Glucose uptake (fmol/cell/hr)

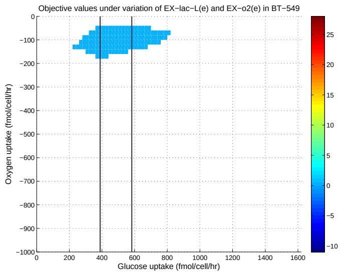
Objective values under variation of EX−lac−L(e) and EX−o2(e) in BT−549
Oxygen uptake (fmol/cell/hr)
Glucose uptake (fmol/cell/hr)

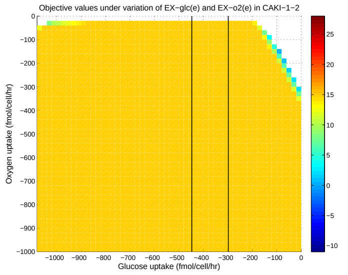
Objective values under variation of EX−glc(e) and EX−o2(e) in CAKI−1−2
Oxygen uptake (fmol/cell/hr)
Glucose uptake (fmol/cell/hr)

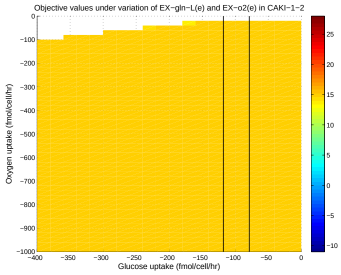
Objective values under variation of EX−gln−L(e) and EX−o2(e) in CAKI−1−2
Oxygen uptake (fmol/cell/hr)
Glucose uptake (fmol/cell/hr)

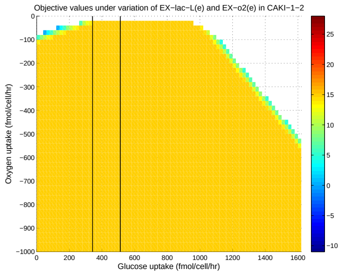
Objective values under variation of EX−lac−L(e) and EX−o2(e) in CAKI−1−2
Oxygen uptake (fmol/cell/hr)
Glucose uptake (fmol/cell/hr)

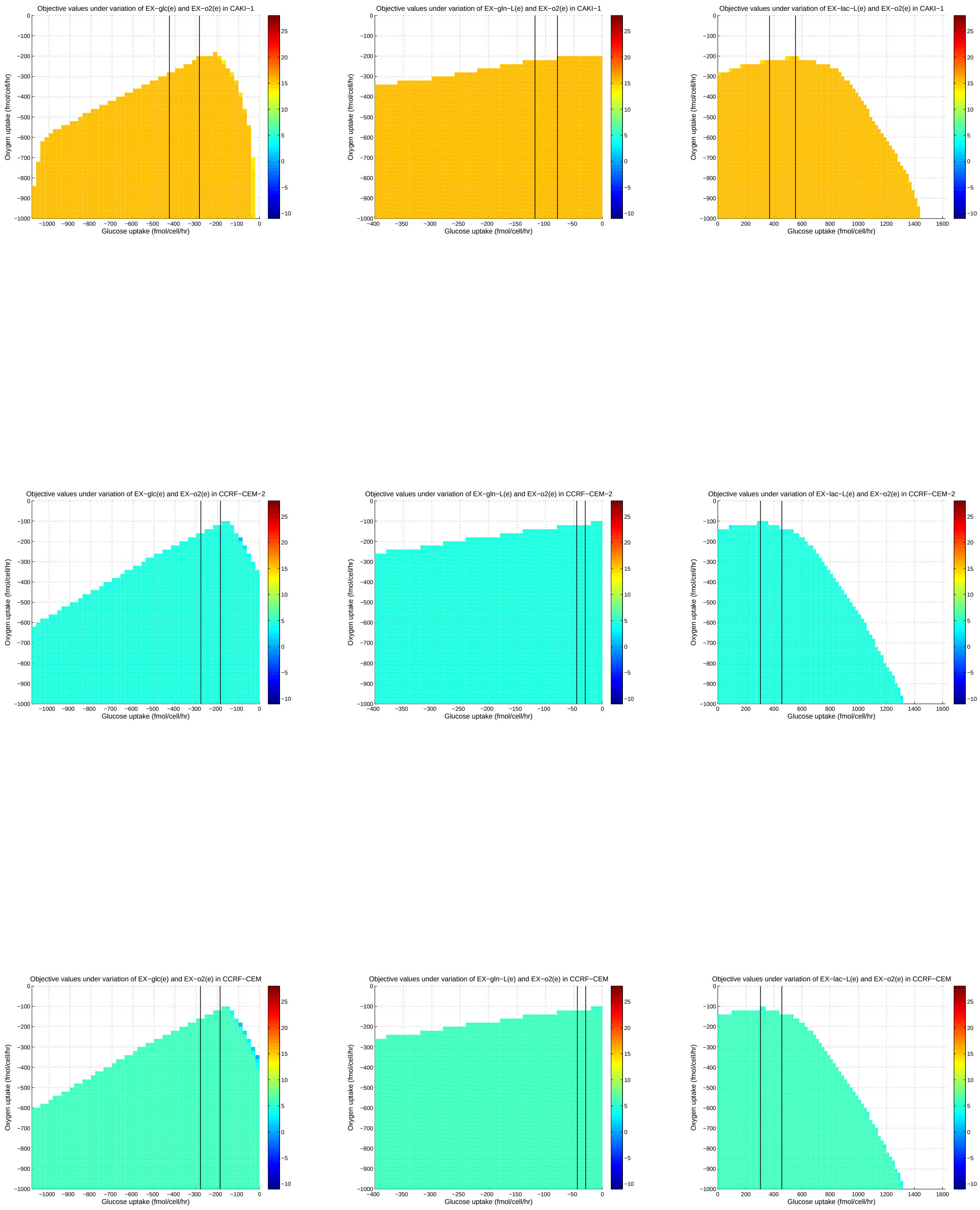
Objective values under variation of EX−glc(e) and EX−o2(e) in CAKI−1
Objective values under variation of EX−gln−L(e) and EX−o2(e) in CAKI−1
Objective values under variation of EX−lac−L(e) and EX−o2(e) in CAKI−1
Objective values under variation of EX−glc(e) and EX−o2(e) in CCRF−CEM−2
Objective values under variation of EX−gln−L(e) and EX−o2(e) in CCRF−CEM−2
Objective values under variation of EX−lac−L(e) and EX−o2(e) in CCRF−CEM−2
Objective values under variation of EX−glc(e) and EX−o2(e) in CCRF−CEM
Objective values under variation of EX−gln−L(e) and EX−o2(e) in CCRF−CEM
Objective values under variation of EX−lac−L(e) and EX−o2(e) in CCRF−CEM
Oxygen uptake (fmol/cell/hr)
Glucose uptake (fmol/cell/hr)

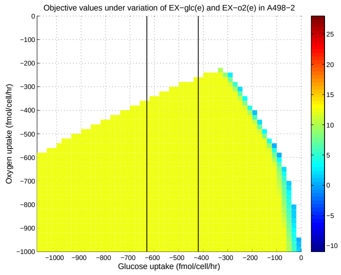
Objective values under variation of EX−glc(e) and EX−o2(e) in A498−2
Oxygen uptake (fmol/cell/hr)
Glucose uptake (fmol/cell/hr)

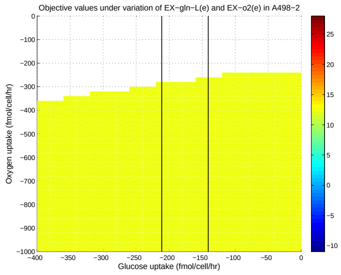
Objective values under variation of EX−gln−L(e) and EX−o2(e) in A498−2
Oxygen uptake (fmol/cell/hr)
Glucose uptake (fmol/cell/hr)

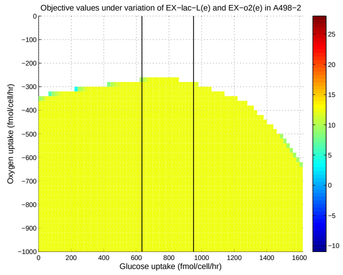
Objective values under variation of EX−lac−L(e) and EX−o2(e) in A498−2
Oxygen uptake (fmol/cell/hr)
Glucose uptake (fmol/cell/hr)

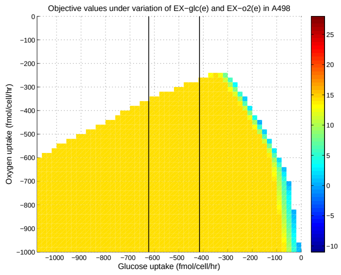
Objective values under variation of EX−glc(e) and EX−o2(e) in A498
Oxygen uptake (fmol/cell/hr)
Glucose uptake (fmol/cell/hr)

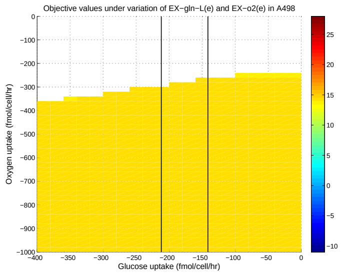
Objective values under variation of EX−gln−L(e) and EX−o2(e) in A498
Oxygen uptake (fmol/cell/hr)
Glucose uptake (fmol/cell/hr)

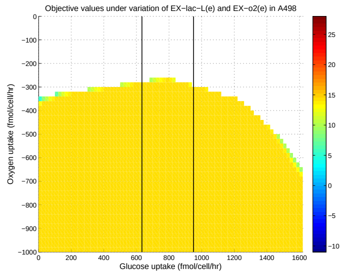
Objective values under variation of EX−lac−L(e) and EX−o2(e) in A498
Oxygen uptake (fmol/cell/hr)
Glucose uptake (fmol/cell/hr)

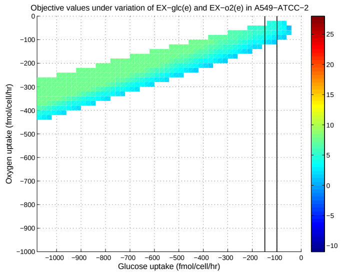
Objective values under variation of EX−glc(e) and EX−o2(e) in A549−ATCC−2
Oxygen uptake (fmol/cell/hr)
Glucose uptake (fmol/cell/hr)

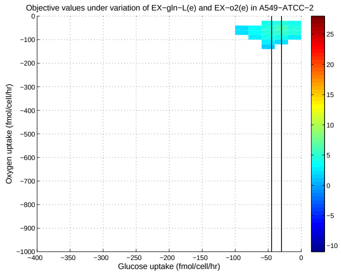
Objective values under variation of EX−gln−L(e) and EX−o2(e) in A549−ATCC−2
Oxygen uptake (fmol/cell/hr)
Glucose uptake (fmol/cell/hr)

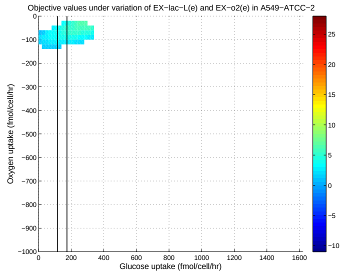
Objective values under variation of EX−lac−L(e) and EX−o2(e) in A549−ATCC−2
Oxygen uptake (fmol/cell/hr)
Glucose uptake (fmol/cell/hr)

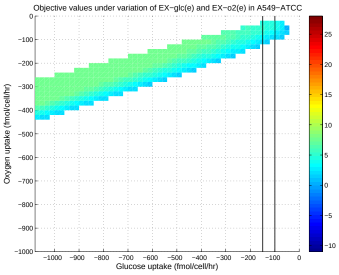
Objective values under variation of EX−glc(e) and EX−o2(e) in A549−ATCC
Oxygen uptake (fmol/cell/hr)
Glucose uptake (fmol/cell/hr)

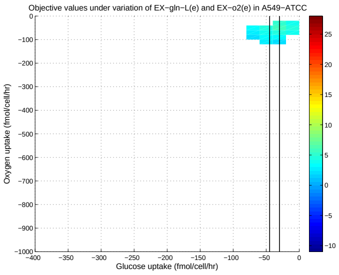
Objective values under variation of EX−gln−L(e) and EX−o2(e) in A549−ATCC
Oxygen uptake (fmol/cell/hr)
Glucose uptake (fmol/cell/hr)

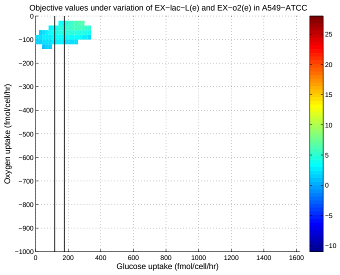
Objective values under variation of EX−lac−L(e) and EX−o2(e) in A549−ATCC
Oxygen uptake (fmol/cell/hr)
Glucose uptake (fmol/cell/hr)

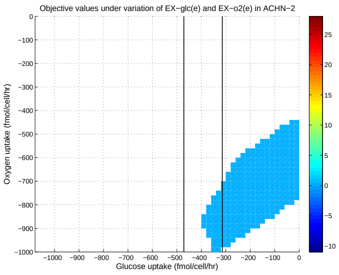
Objective values under variation of EX−glc(e) and EX−o2(e) in ACHN−2
Oxygen uptake (fmol/cell/hr)
Glucose uptake (fmol/cell/hr)

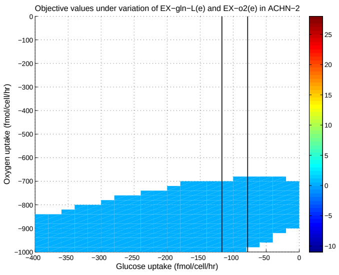
Objective values under variation of EX−gln−L(e) and EX−o2(e) in ACHN−2
Oxygen uptake (fmol/cell/hr)
Glucose uptake (fmol/cell/hr)

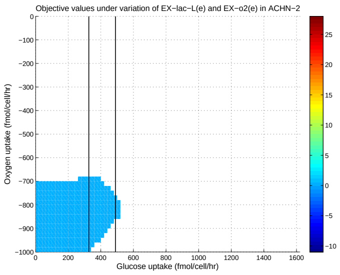
Objective values under variation of EX−lac−L(e) and EX−o2(e) in ACHN−2
Oxygen uptake (fmol/cell/hr)
Glucose uptake (fmol/cell/hr)

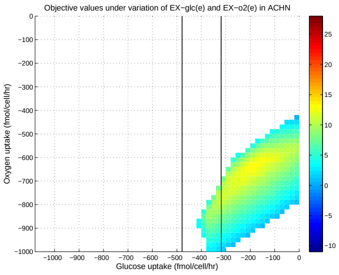
Objective values under variation of EX−glc(e) and EX−o2(e) in ACHN
Oxygen uptake (fmol/cell/hr)
Glucose uptake (fmol/cell/hr)

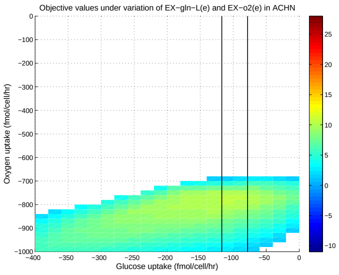
Objective values under variation of EX−gln−L(e) and EX−o2(e) in ACHN
Oxygen uptake (fmol/cell/hr)
Glucose uptake (fmol/cell/hr)

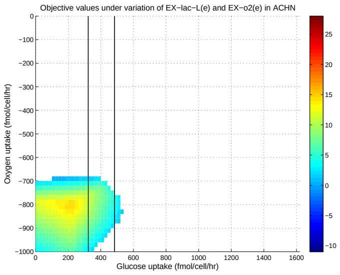
Objective values under variation of EX−lac−L(e) and EX−o2(e) in ACHN
Oxygen uptake (fmol/cell/hr)
Glucose uptake (fmol/cell/hr)

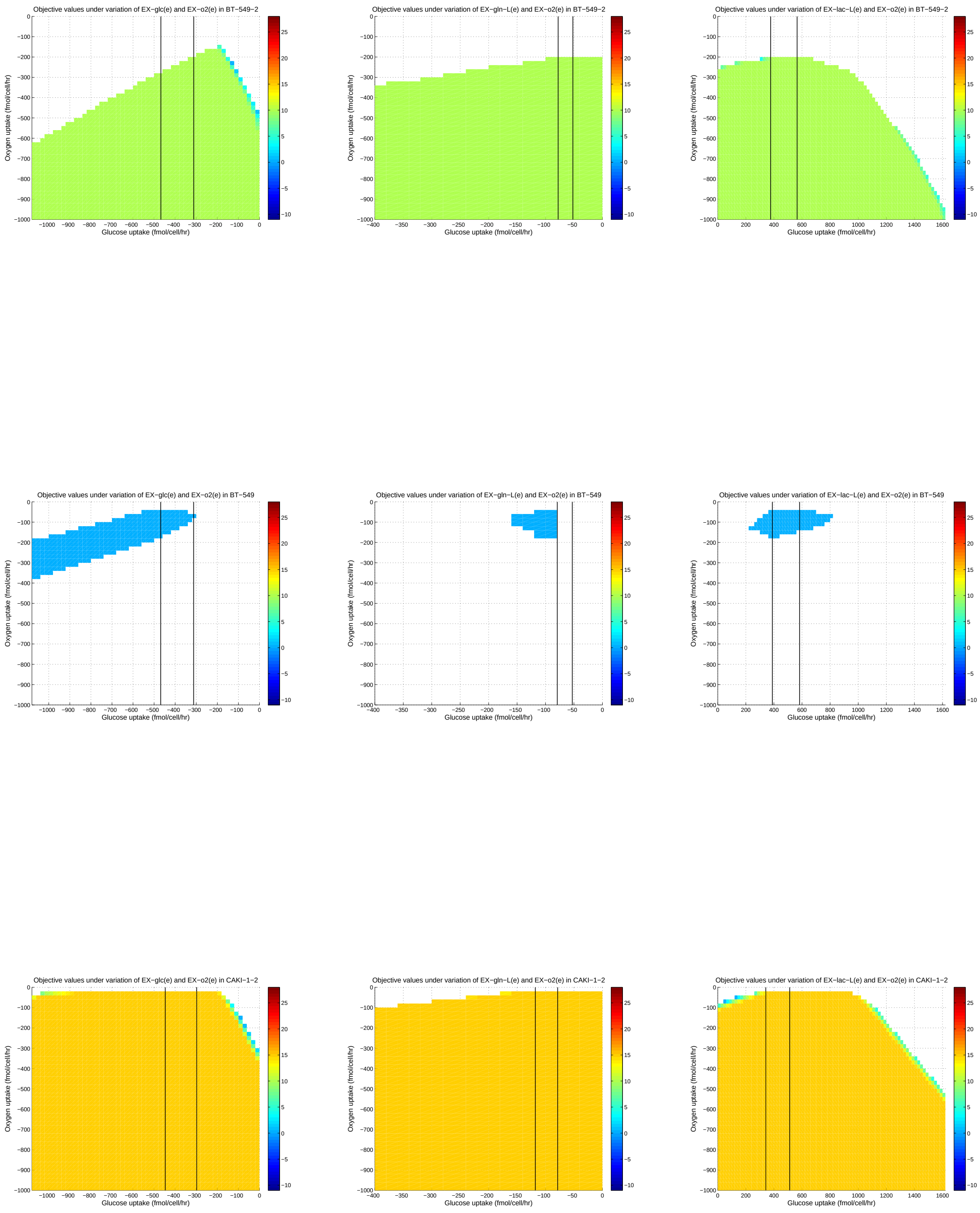
Objective values under variation of EX−glc(e) and EX−o2(e) in BT−549−2
Objective values under variation of EX−gln−L(e) and EX−o2(e) in BT−549−2
Objective values under variation of EX−lac−L(e) and EX−o2(e) in BT−549−2
Objective values under variation of EX−glc(e) and EX−o2(e) in BT−549
Objective values under variation of EX−gln−L(e) and EX−o2(e) in BT−549
Objective values under variation of EX−lac−L(e) and EX−o2(e) in BT−549
Objective values under variation of EX−glc(e) and EX−o2(e) in CAKI−1−2
Objective values under variation of EX−gln−L(e) and EX−o2(e) in CAKI−1−2
Objective values under variation of EX−lac−L(e) and EX−o2(e) in CAKI−1−2
Oxygen uptake (fmol/cell/hr)
Glucose uptake (fmol/cell/hr)

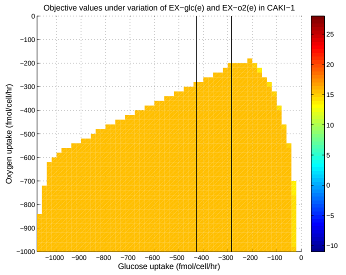
Objective values under variation of EX−glc(e) and EX−o2(e) in CAKI−1
Oxygen uptake (fmol/cell/hr)
Glucose uptake (fmol/cell/hr)

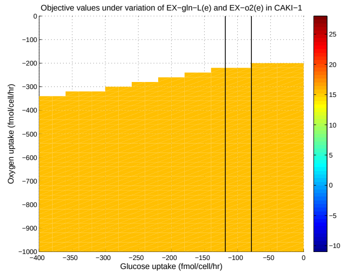
Objective values under variation of EX−gln−L(e) and EX−o2(e) in CAKI−1
Oxygen uptake (fmol/cell/hr)
Glucose uptake (fmol/cell/hr)

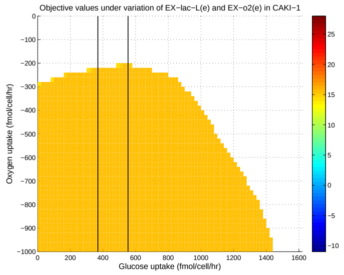
Objective values under variation of EX−lac−L(e) and EX−o2(e) in CAKI−1
Oxygen uptake (fmol/cell/hr)
Glucose uptake (fmol/cell/hr)

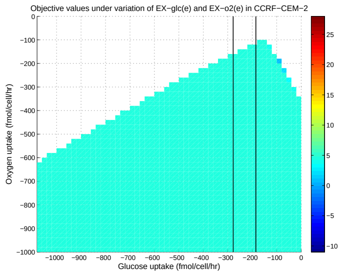
Objective values under variation of EX−glc(e) and EX−o2(e) in CCRF−CEM−2
Oxygen uptake (fmol/cell/hr)
Glucose uptake (fmol/cell/hr)

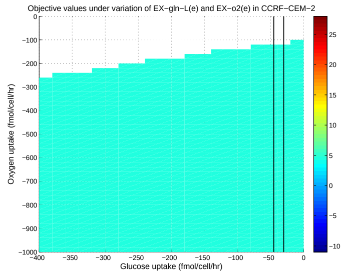
Objective values under variation of EX−gln−L(e) and EX−o2(e) in CCRF−CEM−2
Oxygen uptake (fmol/cell/hr)
Glucose uptake (fmol/cell/hr)

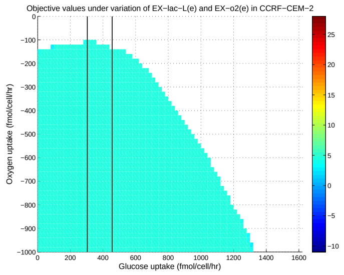
Objective values under variation of EX−lac−L(e) and EX−o2(e) in CCRF−CEM−2
Oxygen uptake (fmol/cell/hr)
Glucose uptake (fmol/cell/hr)

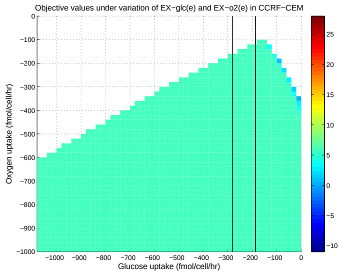
Objective values under variation of EX−glc(e) and EX−o2(e) in CCRF−CEM
Oxygen uptake (fmol/cell/hr)
Glucose uptake (fmol/cell/hr)

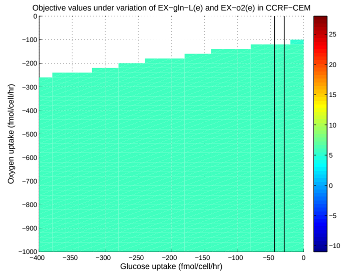
Objective values under variation of EX−gln−L(e) and EX−o2(e) in CCRF−CEM
Oxygen uptake (fmol/cell/hr)
Glucose uptake (fmol/cell/hr)

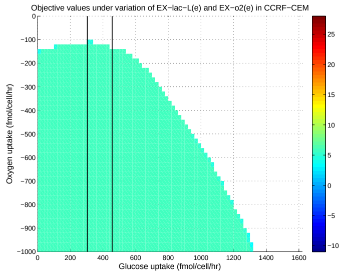
Objective values under variation of EX−lac−L(e) and EX−o2(e) in CCRF−CEM
Oxygen uptake (fmol/cell/hr)
Glucose uptake (fmol/cell/hr)

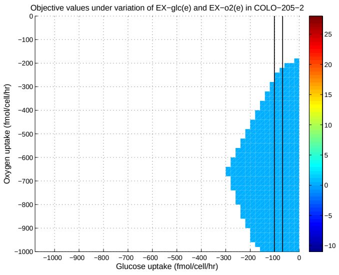
Objective values under variation of EX−glc(e) and EX−o2(e) in COLO−205−2
Oxygen uptake (fmol/cell/hr)
Glucose uptake (fmol/cell/hr)

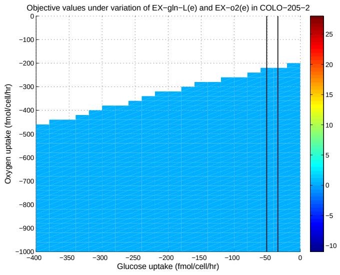
Objective values under variation of EX−gln−L(e) and EX−o2(e) in COLO−205−2
Oxygen uptake (fmol/cell/hr)
Glucose uptake (fmol/cell/hr)

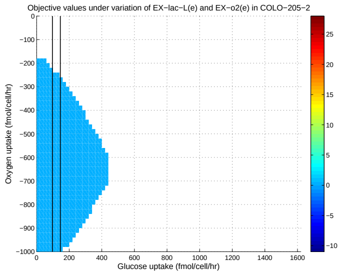
Objective values under variation of EX−lac−L(e) and EX−o2(e) in COLO−205−2
Oxygen uptake (fmol/cell/hr)
Glucose uptake (fmol/cell/hr)

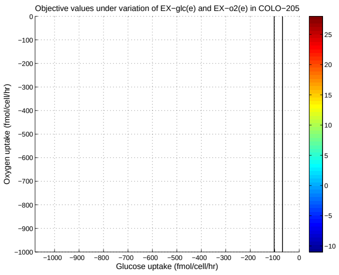
Objective values under variation of EX−glc(e) and EX−o2(e) in COLO−205
Oxygen uptake (fmol/cell/hr)
Glucose uptake (fmol/cell/hr)

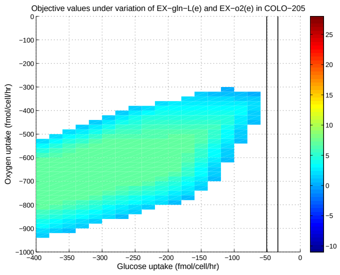
Objective values under variation of EX−gln−L(e) and EX−o2(e) in COLO−205
Oxygen uptake (fmol/cell/hr)
Glucose uptake (fmol/cell/hr)

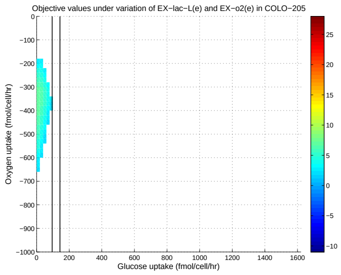
Objective values under variation of EX−lac−L(e) and EX−o2(e) in COLO−205
Oxygen uptake (fmol/cell/hr)
Glucose uptake (fmol/cell/hr)

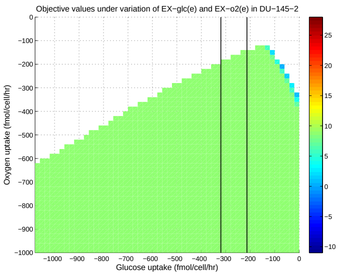
Objective values under variation of EX−glc(e) and EX−o2(e) in DU−145−2
Oxygen uptake (fmol/cell/hr)
Glucose uptake (fmol/cell/hr)

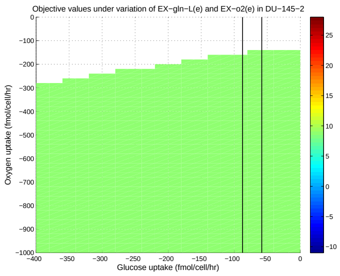
Objective values under variation of EX−gln−L(e) and EX−o2(e) in DU−145−2
Oxygen uptake (fmol/cell/hr)
Glucose uptake (fmol/cell/hr)

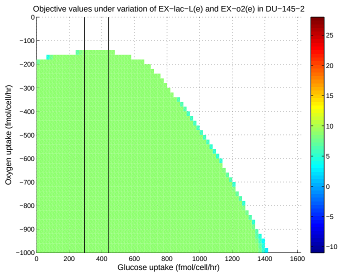
Objective values under variation of EX−lac−L(e) and EX−o2(e) in DU−145−2
Oxygen uptake (fmol/cell/hr)
Glucose uptake (fmol/cell/hr)

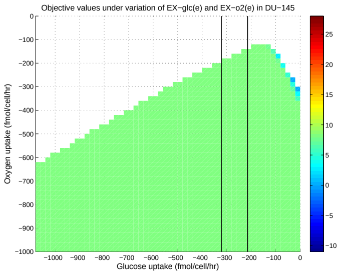
Objective values under variation of EX−glc(e) and EX−o2(e) in DU−145
Oxygen uptake (fmol/cell/hr)
Glucose uptake (fmol/cell/hr)

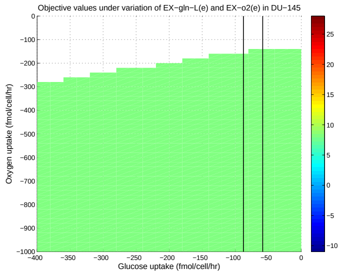
Objective values under variation of EX−gln−L(e) and EX−o2(e) in DU−145
Oxygen uptake (fmol/cell/hr)
Glucose uptake (fmol/cell/hr)

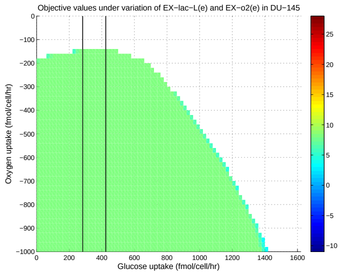
Objective values under variation of EX−lac−L(e) and EX−o2(e) in DU−145
Oxygen uptake (fmol/cell/hr)
Glucose uptake (fmol/cell/hr)

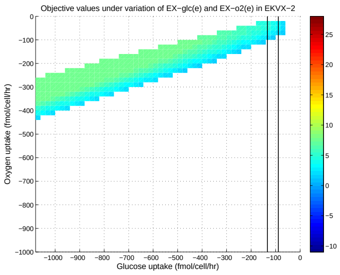
Objective values under variation of EX−glc(e) and EX−o2(e) in EKVX−2
Oxygen uptake (fmol/cell/hr)
Glucose uptake (fmol/cell/hr)

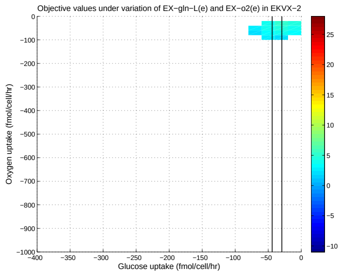
Objective values under variation of EX−gln−L(e) and EX−o2(e) in EKVX−2
Oxygen uptake (fmol/cell/hr)
Glucose uptake (fmol/cell/hr)

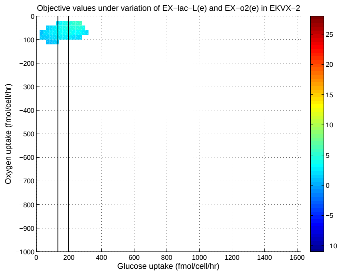
Objective values under variation of EX−lac−L(e) and EX−o2(e) in EKVX−2
Oxygen uptake (fmol/cell/hr)
Glucose uptake (fmol/cell/hr)

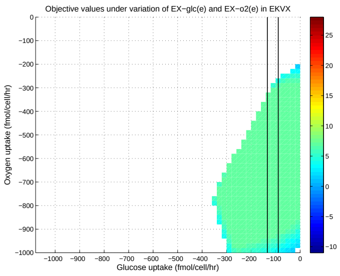
Objective values under variation of EX−glc(e) and EX−o2(e) in EKVX
Oxygen uptake (fmol/cell/hr)
Glucose uptake (fmol/cell/hr)

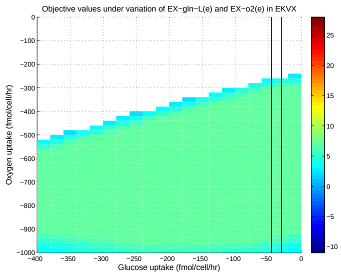
Objective values under variation of EX−gln−L(e) and EX−o2(e) in EKVX
Oxygen uptake (fmol/cell/hr)
Glucose uptake (fmol/cell/hr)

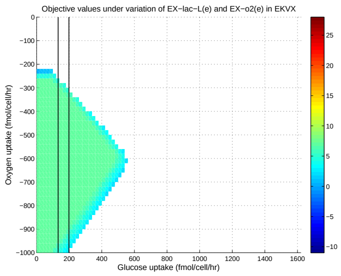
Objective values under variation of EX−lac−L(e) and EX−o2(e) in EKVX
Oxygen uptake (fmol/cell/hr)
Glucose uptake (fmol/cell/hr)

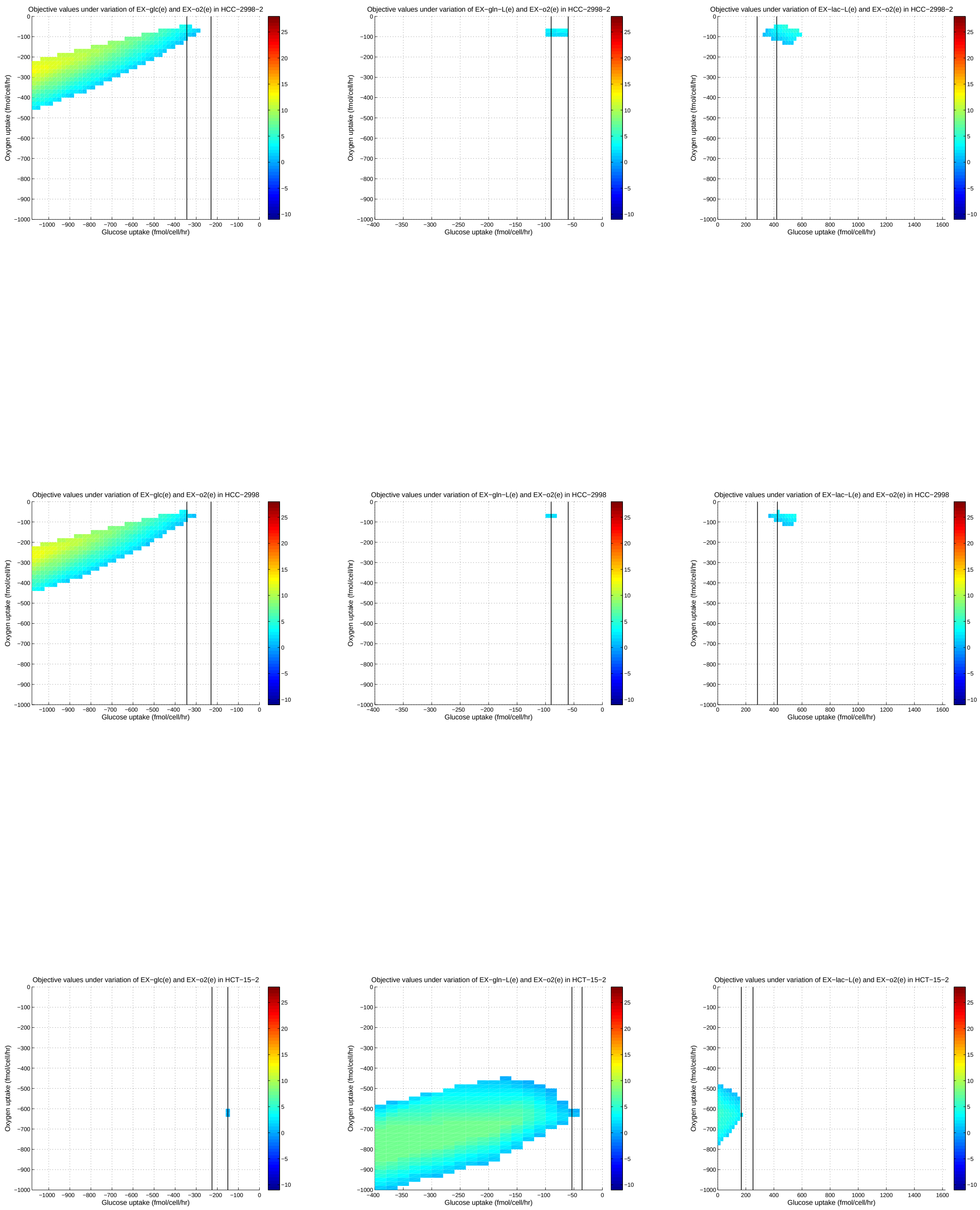

Objective values under variation of EX−glc(e) and EX−o2(e) in HCC−2998−2
Objective values under variation of EX−gln−L(e) and EX−o2(e) in HCC−2998−2
Objective values under variation of EX−lac−L(e) and EX−o2(e) in HCC−2998−2
Objective values under variation of EX−glc(e) and EX−o2(e) in HCC−2998
Objective values under variation of EX−gln−L(e) and EX−o2(e) in HCC−2998
Objective values under variation of EX−lac−L(e) and EX−o2(e) in HCC−2998
Objective values under variation of EX−glc(e) and EX−o2(e) in HCT−15−2
Objective values under variation of EX−gln−L(e) and EX−o2(e) in HCT−15−2
Objective values under variation of EX−lac−L(e) and EX−o2(e) in HCT−15−2
Oxygen uptake (fmol/cell/hr)
Glucose uptake (fmol/cell/hr)

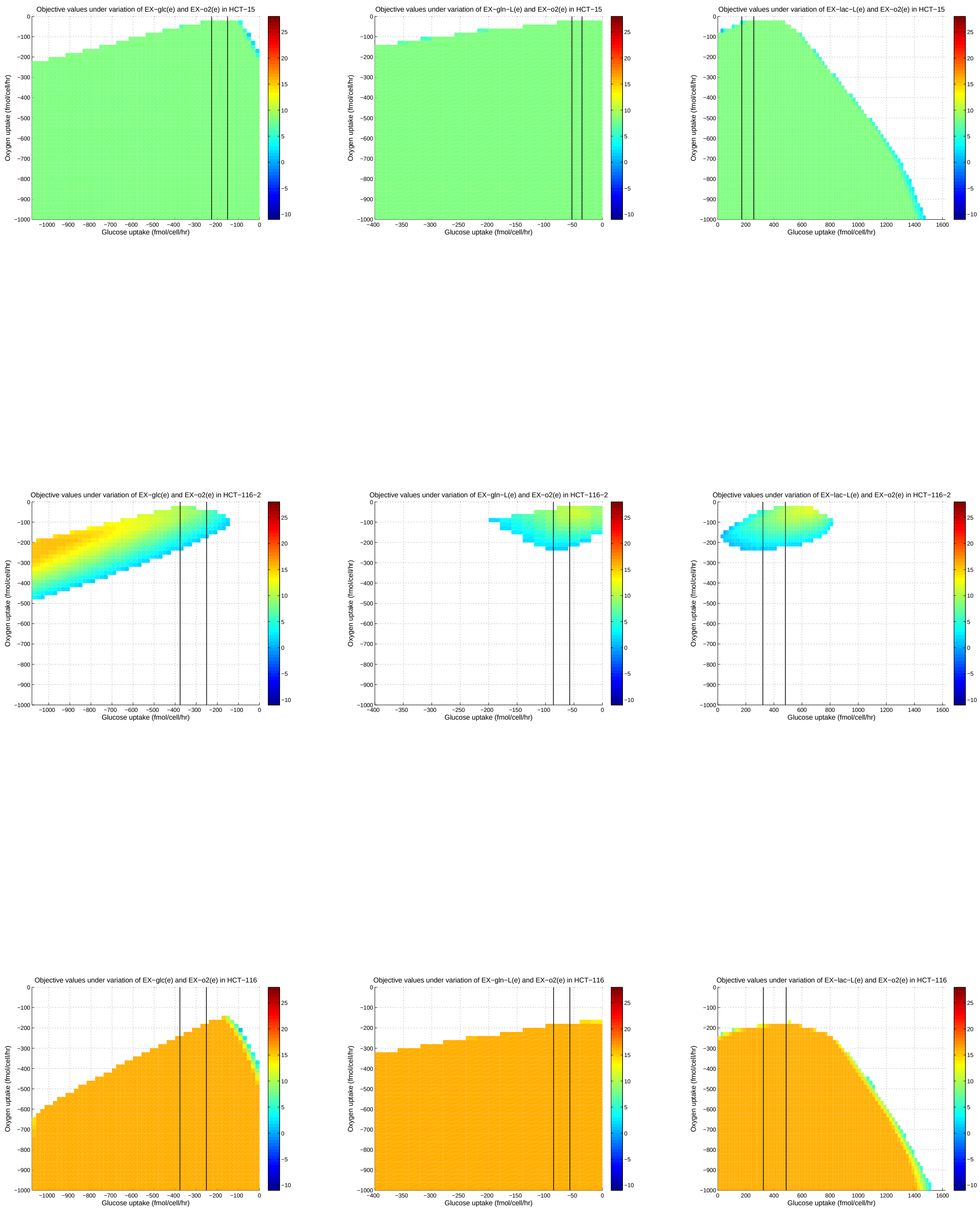
Objective values under variation of EX−glc(e) and EX−o2(e) in HCT−15
Objective values under variation of EX−gln−L(e) and EX−o2(e) in HCT−15
Objective values under variation of EX−lac−L(e) and EX−o2(e) in HCT−15
Objective values under variation of EX−glc(e) and EX−o2(e) in HCT−116−2
Objective values under variation of EX−gln−L(e) and EX−o2(e) in HCT−116−2
Objective values under variation of EX−lac−L(e) and EX−o2(e) in HCT−116−2
Objective values under variation of EX−glc(e) and EX−o2(e) in HCT−116
Objective values under variation of EX−gln−L(e) and EX−o2(e) in HCT−116
Objective values under variation of EX−lac−L(e) and EX−o2(e) in HCT−116
Oxygen uptake (fmol/cell/hr)
Glucose uptake (fmol/cell/hr)

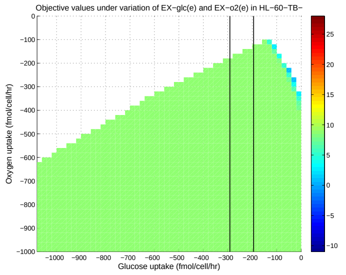
Objective values under variation of EX−glc(e) and EX−o2(e) in HL−60−TB−
Oxygen uptake (fmol/cell/hr)
Glucose uptake (fmol/cell/hr)

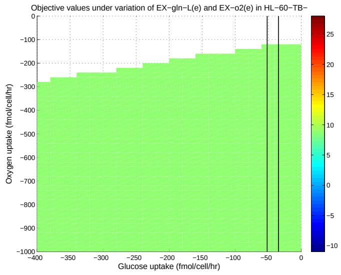
Objective values under variation of EX−gln−L(e) and EX−o2(e) in HL−60−TB−
Oxygen uptake (fmol/cell/hr)
Glucose uptake (fmol/cell/hr)

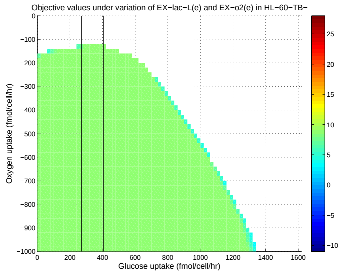
Objective values under variation of EX−lac−L(e) and EX−o2(e) in HL−60−TB−
Oxygen uptake (fmol/cell/hr)
Glucose uptake (fmol/cell/hr)

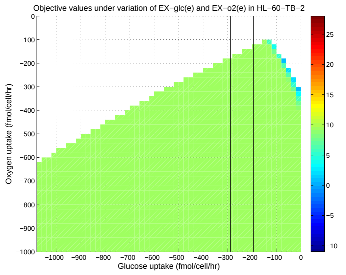
Objective values under variation of EX−glc(e) and EX−o2(e) in HL−60−TB−2
Oxygen uptake (fmol/cell/hr)
Glucose uptake (fmol/cell/hr)

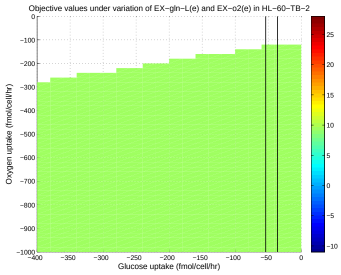
Objective values under variation of EX−gln−L(e) and EX−o2(e) in HL−60−TB−2
Oxygen uptake (fmol/cell/hr)
Glucose uptake (fmol/cell/hr)

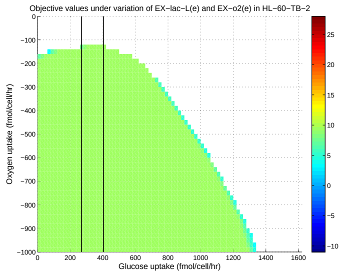
Objective values under variation of EX−lac−L(e) and EX−o2(e) in HL−60−TB−2
Oxygen uptake (fmol/cell/hr)
Glucose uptake (fmol/cell/hr)

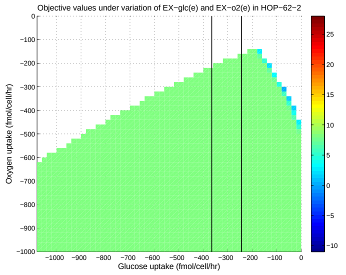
Objective values under variation of EX−glc(e) and EX−o2(e) in HOP−62−2
Oxygen uptake (fmol/cell/hr)
Glucose uptake (fmol/cell/hr)

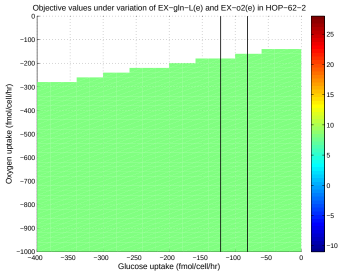
Objective values under variation of EX−gln−L(e) and EX−o2(e) in HOP−62−2
Oxygen uptake (fmol/cell/hr)
Glucose uptake (fmol/cell/hr)

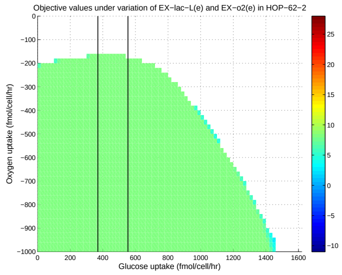
Objective values under variation of EX−lac−L(e) and EX−o2(e) in HOP−62−2
Oxygen uptake (fmol/cell/hr)
Glucose uptake (fmol/cell/hr)

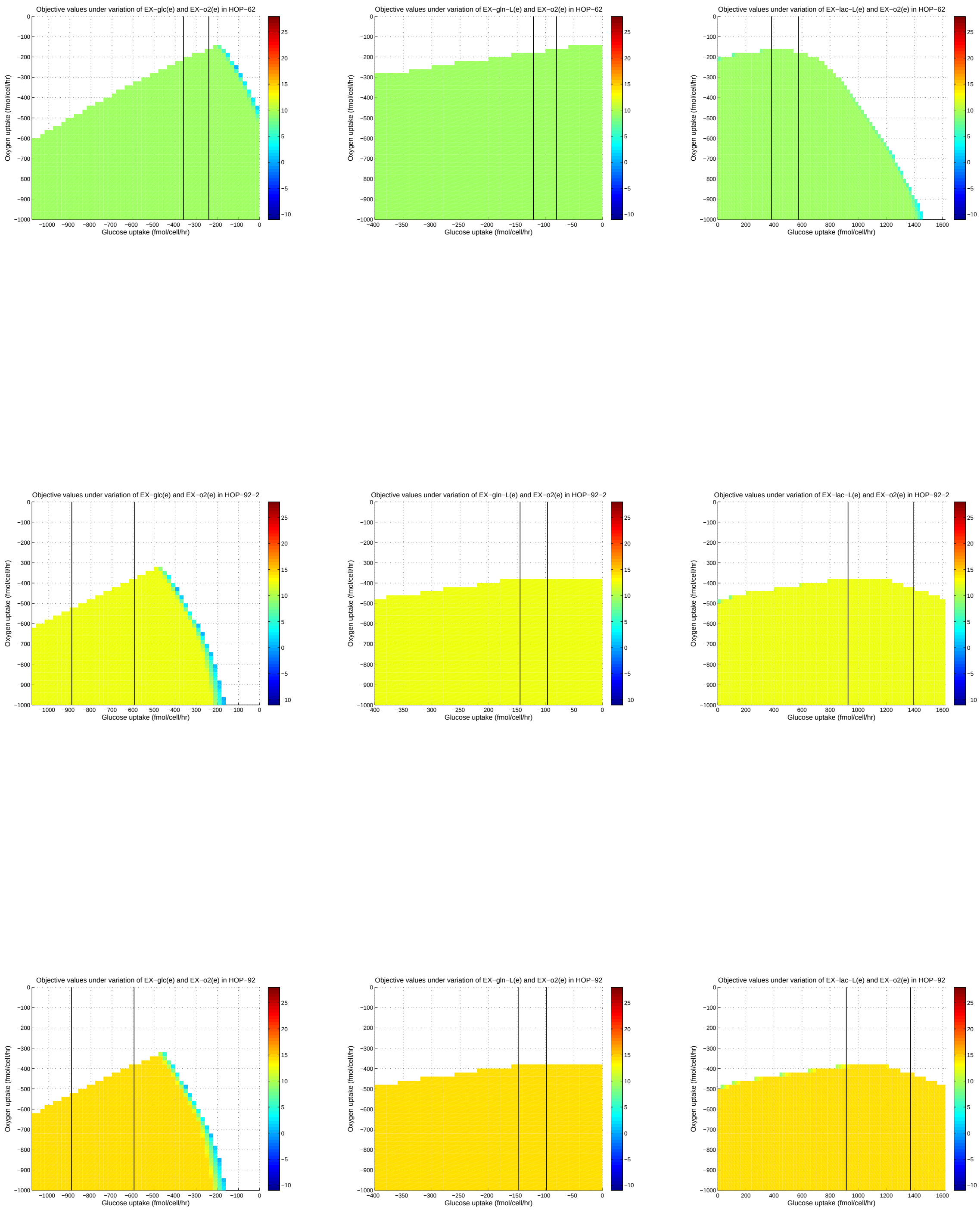

Objective values under variation of EX−glc(e) and EX−o2(e) in HOP−62
Objective values under variation of EX−gln−L(e) and EX−o2(e) in HOP−62
Objective values under variation of EX−lac−L(e) and EX−o2(e) in HOP−62
Objective values under variation of EX−glc(e) and EX−o2(e) in HOP−92−2
Objective values under variation of EX−gln−L(e) and EX−o2(e) in HOP−92−2
Objective values under variation of EX−lac−L(e) and EX−o2(e) in HOP−92−2
Objective values under variation of EX−glc(e) and EX−o2(e) in HOP−92
Objective values under variation of EX−gln−L(e) and EX−o2(e) in HOP−92
Objective values under variation of EX−lac−L(e) and EX−o2(e) in HOP−92
Oxygen uptake (fmol/cell/hr)
Glucose uptake (fmol/cell/hr)

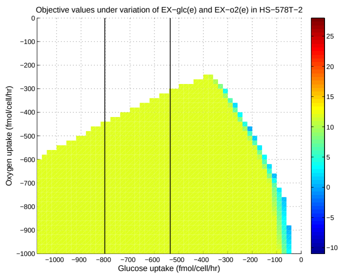
Objective values under variation of EX−glc(e) and EX−o2(e) in HS−578T−2
Oxygen uptake (fmol/cell/hr)
Glucose uptake (fmol/cell/hr)

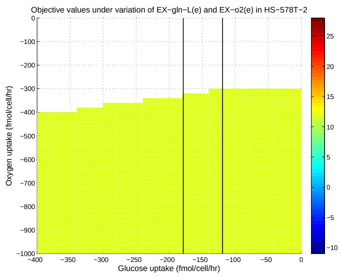
Objective values under variation of EX−gln−L(e) and EX−o2(e) in HS−578T−2
Oxygen uptake (fmol/cell/hr)
Glucose uptake (fmol/cell/hr)

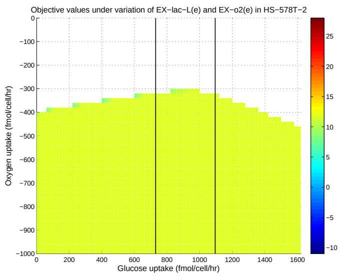
Objective values under variation of EX−lac−L(e) and EX−o2(e) in HS−578T−2
Oxygen uptake (fmol/cell/hr)
Glucose uptake (fmol/cell/hr)

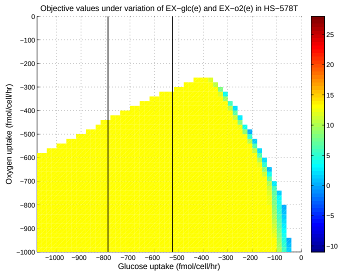
Objective values under variation of EX−glc(e) and EX−o2(e) in HS−578T
Oxygen uptake (fmol/cell/hr)
Glucose uptake (fmol/cell/hr)

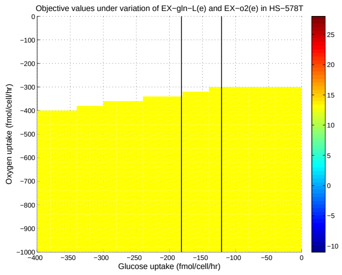
Objective values under variation of EX−gln−L(e) and EX−o2(e) in HS−578T
Oxygen uptake (fmol/cell/hr)
Glucose uptake (fmol/cell/hr)

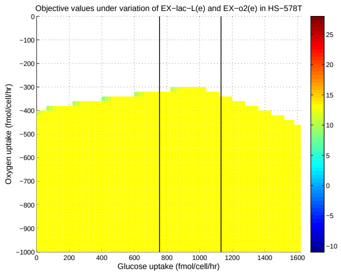
Objective values under variation of EX−lac−L(e) and EX−o2(e) in HS−578T
Oxygen uptake (fmol/cell/hr)
Glucose uptake (fmol/cell/hr)

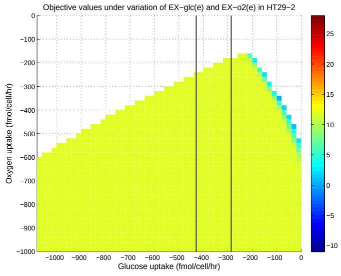
Objective values under variation of EX−glc(e) and EX−o2(e) in HT29−2
Oxygen uptake (fmol/cell/hr)
Glucose uptake (fmol/cell/hr)

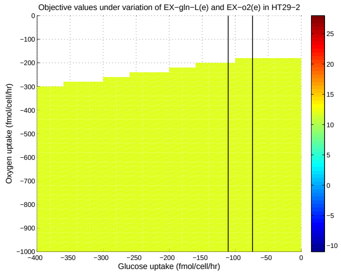
Objective values under variation of EX−gln−L(e) and EX−o2(e) in HT29−2
Oxygen uptake (fmol/cell/hr)
Glucose uptake (fmol/cell/hr)

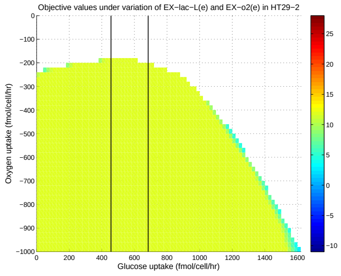
Objective values under variation of EX−lac−L(e) and EX−o2(e) in HT29−2
Oxygen uptake (fmol/cell/hr)
Glucose uptake (fmol/cell/hr)

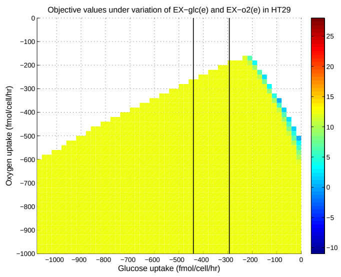
Objective values under variation of EX−glc(e) and EX−o2(e) in HT29
Oxygen uptake (fmol/cell/hr)
Glucose uptake (fmol/cell/hr)

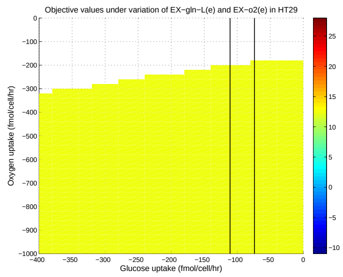
Objective values under variation of EX−gln−L(e) and EX−o2(e) in HT29
Oxygen uptake (fmol/cell/hr)
Glucose uptake (fmol/cell/hr)

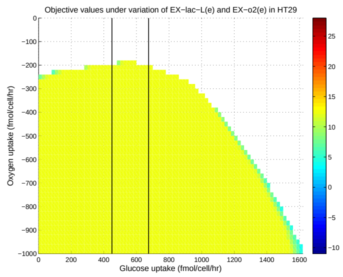
Objective values under variation of EX−lac−L(e) and EX−o2(e) in HT29
Oxygen uptake (fmol/cell/hr)
Glucose uptake (fmol/cell/hr)

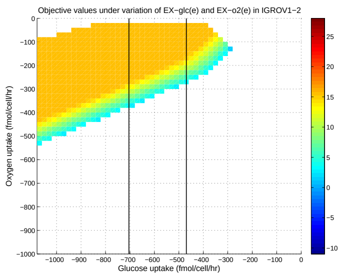
Objective values under variation of EX−glc(e) and EX−o2(e) in IGROV1−2
Oxygen uptake (fmol/cell/hr)
Glucose uptake (fmol/cell/hr)

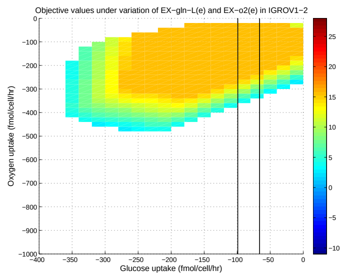
Objective values under variation of EX−gln−L(e) and EX−o2(e) in IGROV1−2
Oxygen uptake (fmol/cell/hr)
Glucose uptake (fmol/cell/hr)

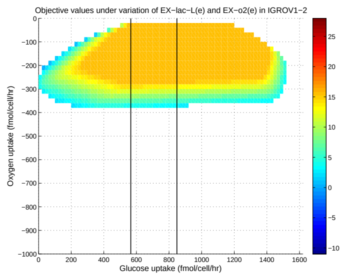
Objective values under variation of EX−lac−L(e) and EX−o2(e) in IGROV1−2
Oxygen uptake (fmol/cell/hr)
Glucose uptake (fmol/cell/hr)

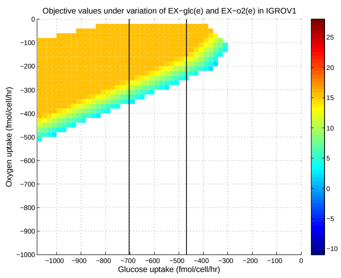
Objective values under variation of EX−glc(e) and EX−o2(e) in IGROV1
Oxygen uptake (fmol/cell/hr)
Glucose uptake (fmol/cell/hr)

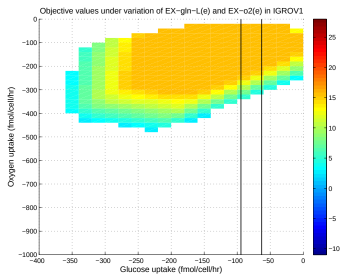
Objective values under variation of EX−gln−L(e) and EX−o2(e) in IGROV1
Oxygen uptake (fmol/cell/hr)
Glucose uptake (fmol/cell/hr)

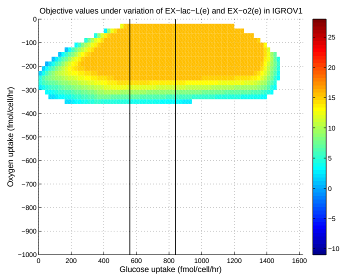
Objective values under variation of EX−lac−L(e) and EX−o2(e) in IGROV1
Oxygen uptake (fmol/cell/hr)
Glucose uptake (fmol/cell/hr)

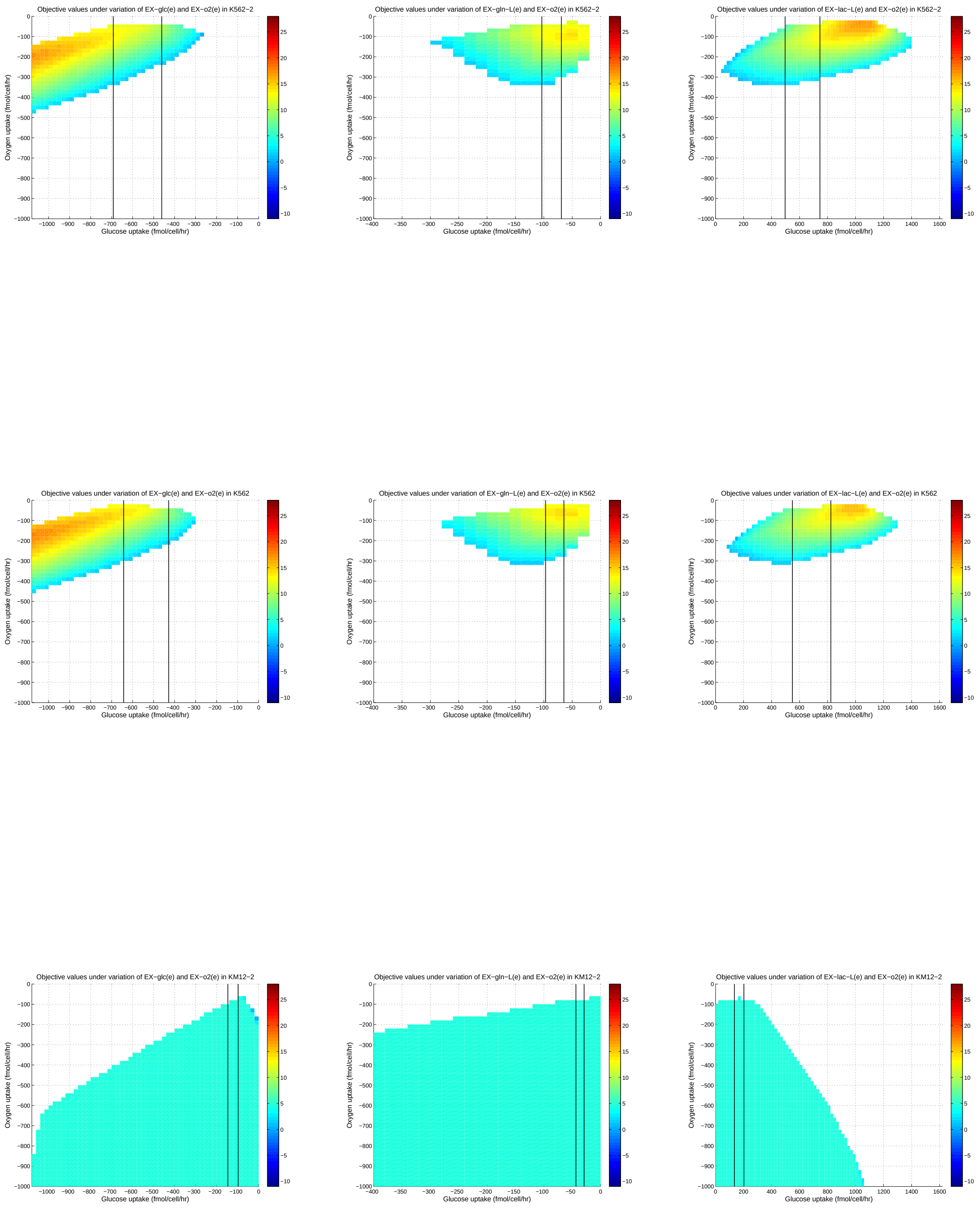
Objective values under variation of EX−glc(e) and EX−o2(e) in K562−2
Objective values under variation of EX−gln−L(e) and EX−o2(e) in K562−2
Objective values under variation of EX−lac−L(e) and EX−o2(e) in K562−2
Objective values under variation of EX−glc(e) and EX−o2(e) in K562
Objective values under variation of EX−gln−L(e) and EX−o2(e) in K562
Objective values under variation of EX−lac−L(e) and EX−o2(e) in K562
Objective values under variation of EX−glc(e) and EX−o2(e) in KM12−2
Objective values under variation of EX−gln−L(e) and EX−o2(e) in KM12−2
Objective values under variation of EX−lac−L(e) and EX−o2(e) in KM12−2
Oxygen uptake (fmol/cell/hr)
Glucose uptake (fmol/cell/hr)

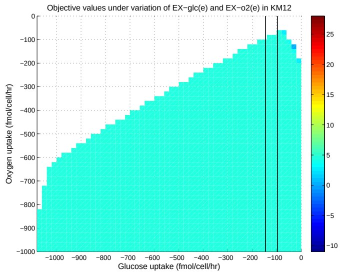
Objective values under variation of EX−glc(e) and EX−o2(e) in KM12
Oxygen uptake (fmol/cell/hr)
Glucose uptake (fmol/cell/hr)

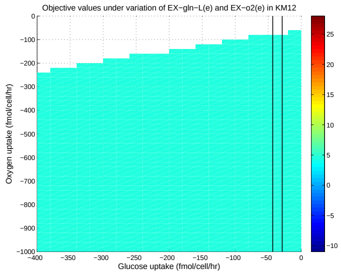
Objective values under variation of EX−gln−L(e) and EX−o2(e) in KM12
Oxygen uptake (fmol/cell/hr)
Glucose uptake (fmol/cell/hr)

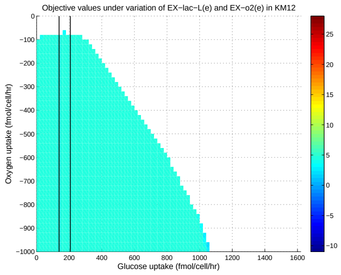
Objective values under variation of EX−lac−L(e) and EX−o2(e) in KM12
Oxygen uptake (fmol/cell/hr)
Glucose uptake (fmol/cell/hr)

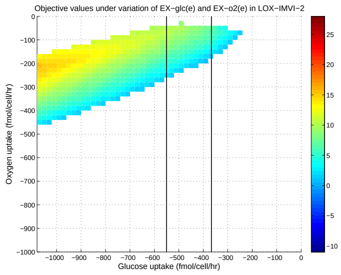
Objective values under variation of EX−glc(e) and EX−o2(e) in LOX−IMVI−2
Oxygen uptake (fmol/cell/hr)
Glucose uptake (fmol/cell/hr)

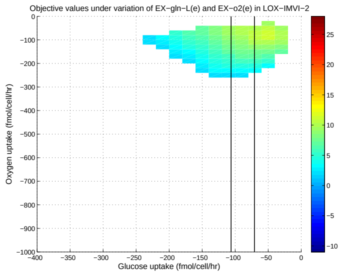
Objective values under variation of EX−gln−L(e) and EX−o2(e) in LOX−IMVI−2
Oxygen uptake (fmol/cell/hr)
Glucose uptake (fmol/cell/hr)

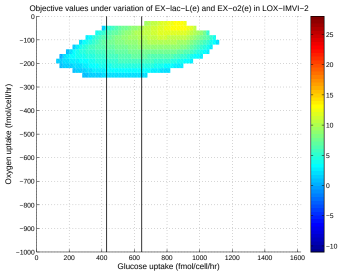
Objective values under variation of EX−lac−L(e) and EX−o2(e) in LOX−IMVI−2
Oxygen uptake (fmol/cell/hr)
Glucose uptake (fmol/cell/hr)

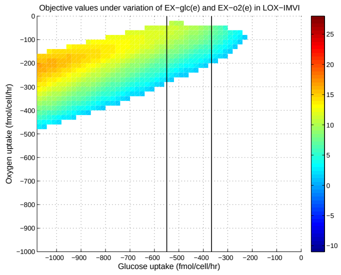
Objective values under variation of EX−glc(e) and EX−o2(e) in LOX−IMVI
Oxygen uptake (fmol/cell/hr)
Glucose uptake (fmol/cell/hr)

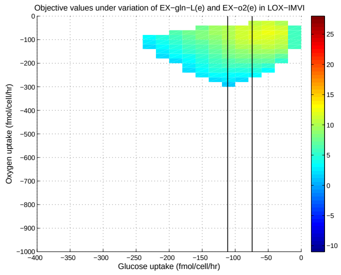
Objective values under variation of EX−gln−L(e) and EX−o2(e) in LOX−IMVI
Oxygen uptake (fmol/cell/hr)
Glucose uptake (fmol/cell/hr)

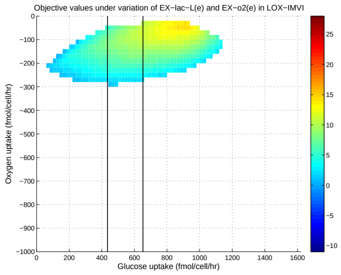
Objective values under variation of EX−lac−L(e) and EX−o2(e) in LOX−IMVI
Oxygen uptake (fmol/cell/hr)
Glucose uptake (fmol/cell/hr)

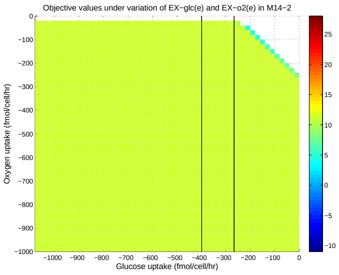
Objective values under variation of EX−glc(e) and EX−o2(e) in M14−2
Oxygen uptake (fmol/cell/hr)
Glucose uptake (fmol/cell/hr)

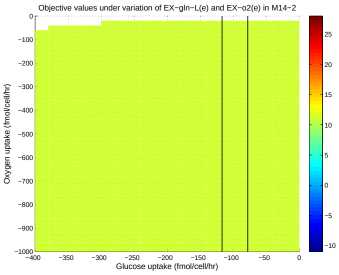
Objective values under variation of EX−gln−L(e) and EX−o2(e) in M14−2
Oxygen uptake (fmol/cell/hr)
Glucose uptake (fmol/cell/hr)

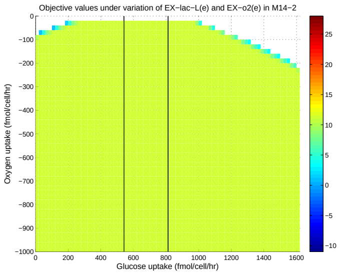
Objective values under variation of EX−lac−L(e) and EX−o2(e) in M14−2
Oxygen uptake (fmol/cell/hr)
Glucose uptake (fmol/cell/hr)

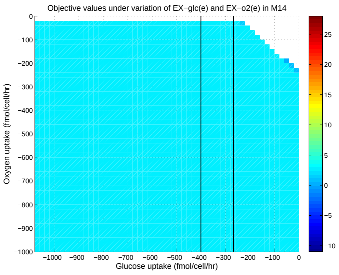
Objective values under variation of EX−glc(e) and EX−o2(e) in M14
Oxygen uptake (fmol/cell/hr)
Glucose uptake (fmol/cell/hr)

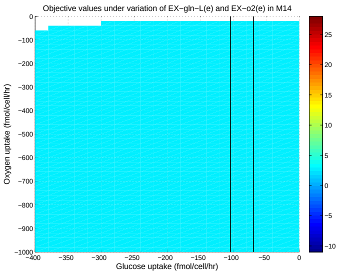
Objective values under variation of EX−gln−L(e) and EX−o2(e) in M14
Oxygen uptake (fmol/cell/hr)
Glucose uptake (fmol/cell/hr)

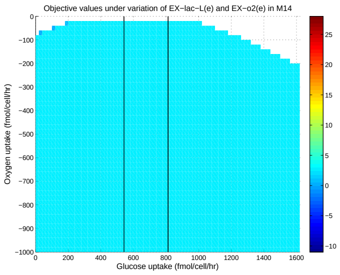
Objective values under variation of EX−lac−L(e) and EX−o2(e) in M14
Oxygen uptake (fmol/cell/hr)
Glucose uptake (fmol/cell/hr)

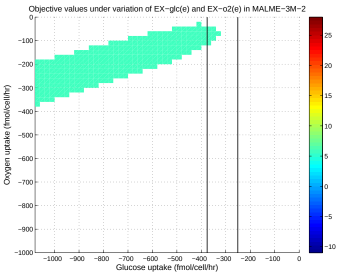
Objective values under variation of EX−glc(e) and EX−o2(e) in MALME−3M−2
Oxygen uptake (fmol/cell/hr)
Glucose uptake (fmol/cell/hr)

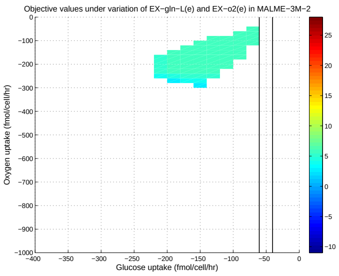
Objective values under variation of EX−gln−L(e) and EX−o2(e) in MALME−3M−2
Oxygen uptake (fmol/cell/hr)
Glucose uptake (fmol/cell/hr)

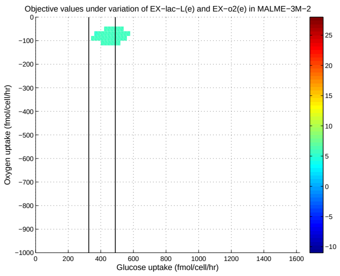
Objective values under variation of EX−lac−L(e) and EX−o2(e) in MALME−3M−2
Oxygen uptake (fmol/cell/hr)
Glucose uptake (fmol/cell/hr)

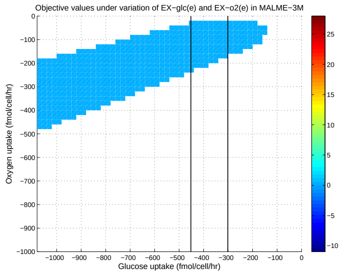
Objective values under variation of EX−glc(e) and EX−o2(e) in MALME−3M
Oxygen uptake (fmol/cell/hr)
Glucose uptake (fmol/cell/hr)

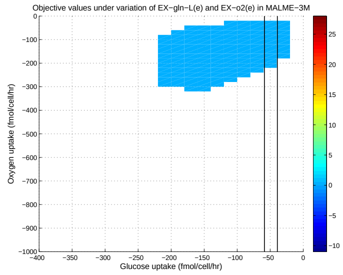
Objective values under variation of EX−gln−L(e) and EX−o2(e) in MALME−3M
Oxygen uptake (fmol/cell/hr)
Glucose uptake (fmol/cell/hr)

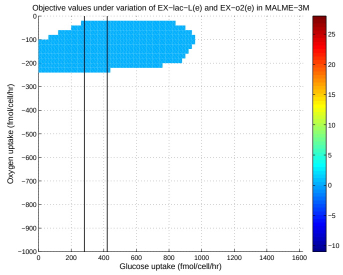
Objective values under variation of EX−lac−L(e) and EX−o2(e) in MALME−3M
Oxygen uptake (fmol/cell/hr)
Glucose uptake (fmol/cell/hr)

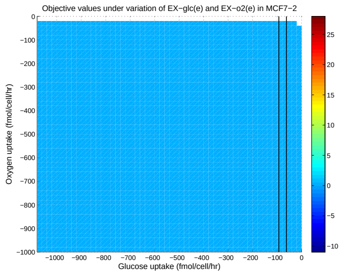
Objective values under variation of EX−glc(e) and EX−o2(e) in MCF7−2
Oxygen uptake (fmol/cell/hr)
Glucose uptake (fmol/cell/hr)

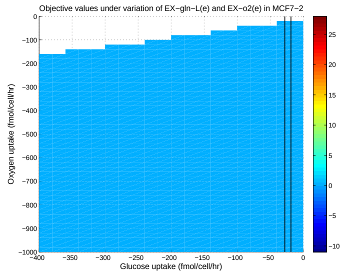
Objective values under variation of EX−gln−L(e) and EX−o2(e) in MCF7−2
Oxygen uptake (fmol/cell/hr)
Glucose uptake (fmol/cell/hr)

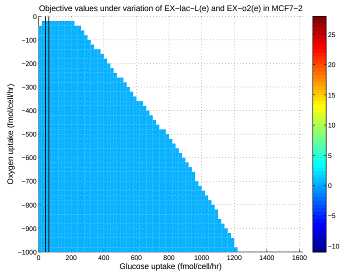
Objective values under variation of EX−lac−L(e) and EX−o2(e) in MCF7−2
Oxygen uptake (fmol/cell/hr)
Glucose uptake (fmol/cell/hr)

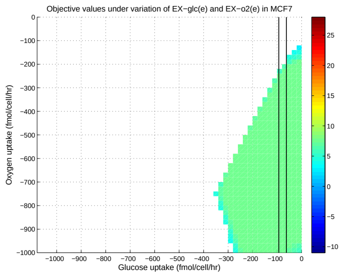
Objective values under variation of EX−glc(e) and EX−o2(e) in MCF7
Oxygen uptake (fmol/cell/hr)
Glucose uptake (fmol/cell/hr)

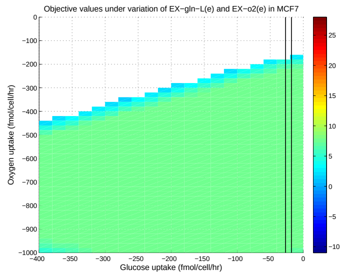
Objective values under variation of EX−gln−L(e) and EX−o2(e) in MCF7
Oxygen uptake (fmol/cell/hr)
Glucose uptake (fmol/cell/hr)

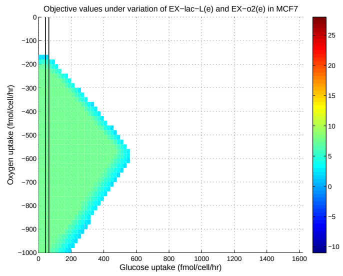
Objective values under variation of EX−lac−L(e) and EX−o2(e) in MCF7
Oxygen uptake (fmol/cell/hr)
Glucose uptake (fmol/cell/hr)

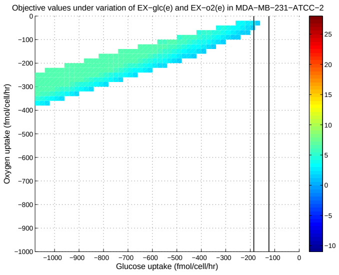
Objective values under variation of EX−glc(e) and EX−o2(e) in MDA−MB−231−ATCC−2
Oxygen uptake (fmol/cell/hr)
Glucose uptake (fmol/cell/hr)

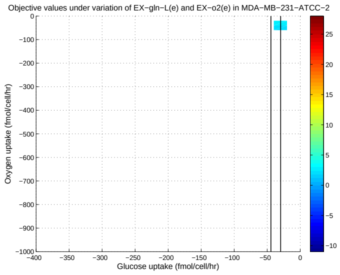
Objective values under variation of EX−gln−L(e) and EX−o2(e) in MDA−MB−231−ATCC−2
Oxygen uptake (fmol/cell/hr)
Glucose uptake (fmol/cell/hr)

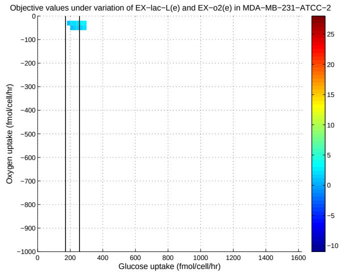
Objective values under variation of EX−lac−L(e) and EX−o2(e) in MDA−MB−231−ATCC−2
Oxygen uptake (fmol/cell/hr)
Glucose uptake (fmol/cell/hr)

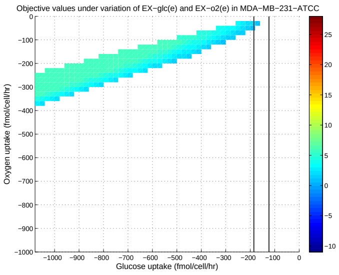
Objective values under variation of EX−glc(e) and EX−o2(e) in MDA−MB−231−ATCC
Oxygen uptake (fmol/cell/hr)
Glucose uptake (fmol/cell/hr)

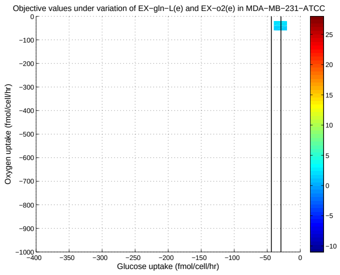
Objective values under variation of EX−gln−L(e) and EX−o2(e) in MDA−MB−231−ATCC
Oxygen uptake (fmol/cell/hr)
Glucose uptake (fmol/cell/hr)

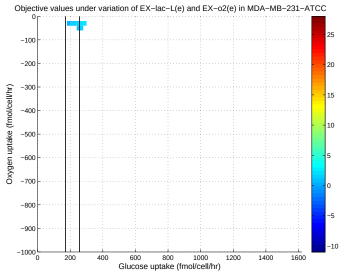
Objective values under variation of EX−lac−L(e) and EX−o2(e) in MDA−MB−231−ATCC
Oxygen uptake (fmol/cell/hr)
Glucose uptake (fmol/cell/hr)

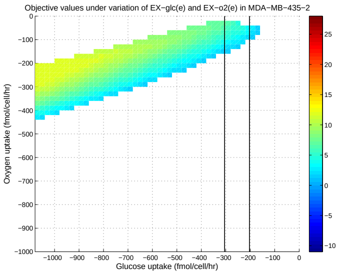
Objective values under variation of EX−glc(e) and EX−o2(e) in MDA−MB−435−2
Oxygen uptake (fmol/cell/hr)
Glucose uptake (fmol/cell/hr)

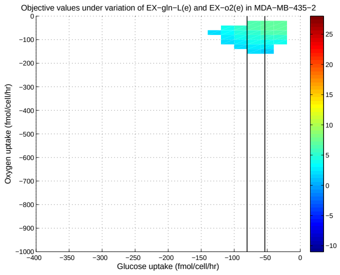
Objective values under variation of EX−gln−L(e) and EX−o2(e) in MDA−MB−435−2
Oxygen uptake (fmol/cell/hr)
Glucose uptake (fmol/cell/hr)

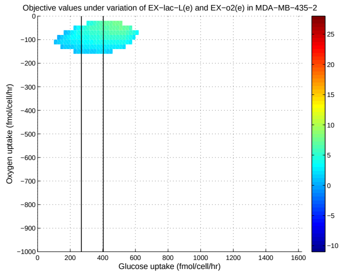
Objective values under variation of EX−lac−L(e) and EX−o2(e) in MDA−MB−435−2
Oxygen uptake (fmol/cell/hr)
Glucose uptake (fmol/cell/hr)

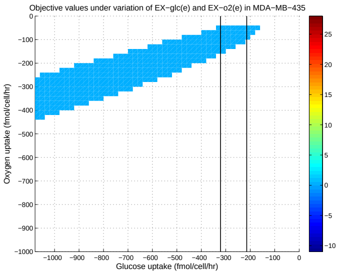
Objective values under variation of EX−glc(e) and EX−o2(e) in MDA−MB−435
Oxygen uptake (fmol/cell/hr)
Glucose uptake (fmol/cell/hr)

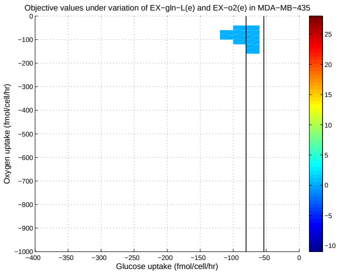
Objective values under variation of EX−gln−L(e) and EX−o2(e) in MDA−MB−435
Oxygen uptake (fmol/cell/hr)
Glucose uptake (fmol/cell/hr)

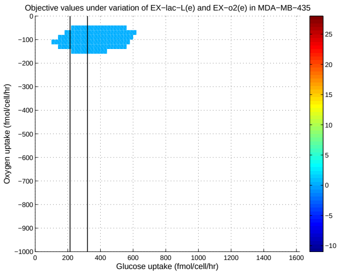
Objective values under variation of EX−lac−L(e) and EX−o2(e) in MDA−MB−435
Oxygen uptake (fmol/cell/hr)
Glucose uptake (fmol/cell/hr)

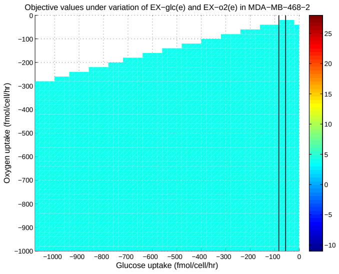
Objective values under variation of EX−glc(e) and EX−o2(e) in MDA−MB−468−2
Oxygen uptake (fmol/cell/hr)
Glucose uptake (fmol/cell/hr)

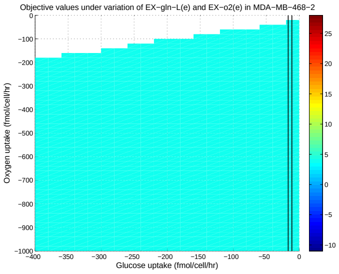
Objective values under variation of EX−gln−L(e) and EX−o2(e) in MDA−MB−468−2
Oxygen uptake (fmol/cell/hr)
Glucose uptake (fmol/cell/hr)

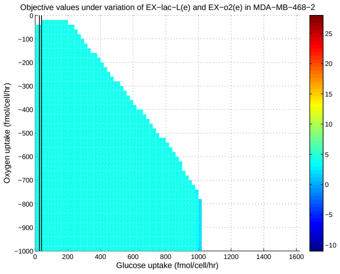
Objective values under variation of EX−lac−L(e) and EX−o2(e) in MDA−MB−468−2
Oxygen uptake (fmol/cell/hr)
Glucose uptake (fmol/cell/hr)

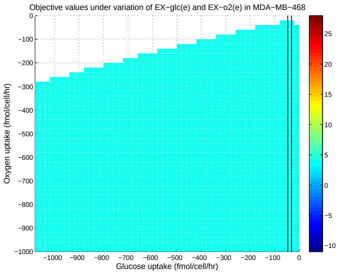
Objective values under variation of EX−glc(e) and EX−o2(e) in MDA−MB−468
Oxygen uptake (fmol/cell/hr)
Glucose uptake (fmol/cell/hr)

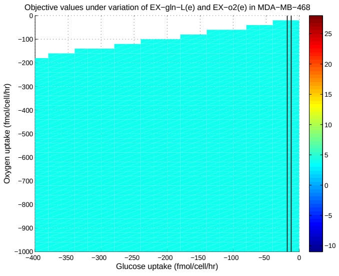
Objective values under variation of EX−gln−L(e) and EX−o2(e) in MDA−MB−468
Oxygen uptake (fmol/cell/hr)
Glucose uptake (fmol/cell/hr)

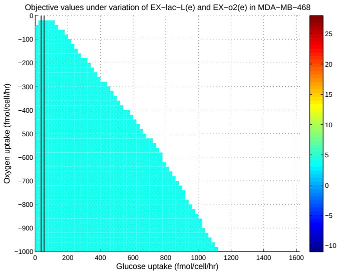
Objective values under variation of EX−lac−L(e) and EX−o2(e) in MDA−MB−468
Oxygen uptake (fmol/cell/hr)
Glucose uptake (fmol/cell/hr)

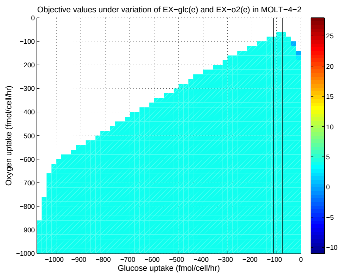
Objective values under variation of EX−glc(e) and EX−o2(e) in MOLT−4−2
Oxygen uptake (fmol/cell/hr)
Glucose uptake (fmol/cell/hr)

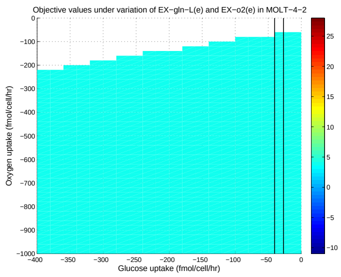
Objective values under variation of EX−gln−L(e) and EX−o2(e) in MOLT−4−2
Oxygen uptake (fmol/cell/hr)
Glucose uptake (fmol/cell/hr)

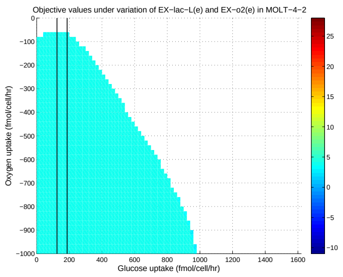
Objective values under variation of EX−lac−L(e) and EX−o2(e) in MOLT−4−2
Oxygen uptake (fmol/cell/hr)
Glucose uptake (fmol/cell/hr)

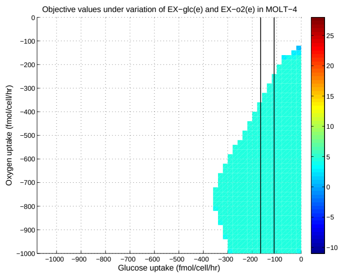
Objective values under variation of EX−glc(e) and EX−o2(e) in MOLT−4
Oxygen uptake (fmol/cell/hr)
Glucose uptake (fmol/cell/hr)

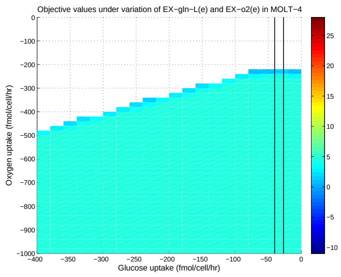
Objective values under variation of EX−gln−L(e) and EX−o2(e) in MOLT−4
Oxygen uptake (fmol/cell/hr)
Glucose uptake (fmol/cell/hr)

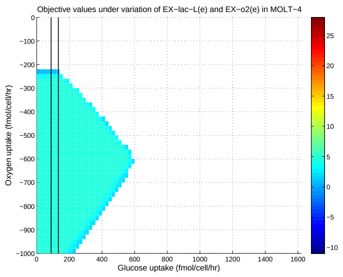
Objective values under variation of EX−lac−L(e) and EX−o2(e) in MOLT−4
Oxygen uptake (fmol/cell/hr)
Glucose uptake (fmol/cell/hr)

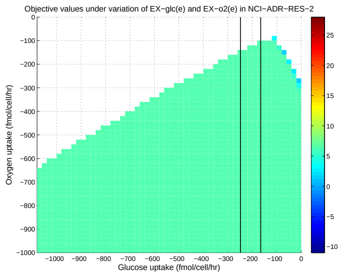
Objective values under variation of EX−glc(e) and EX−o2(e) in NCI−ADR−RES−2
Oxygen uptake (fmol/cell/hr)
Glucose uptake (fmol/cell/hr)

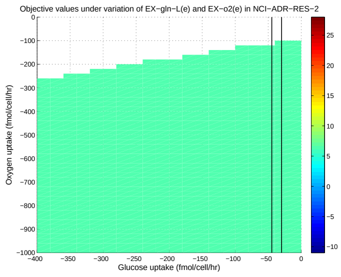
Objective values under variation of EX−gln−L(e) and EX−o2(e) in NCI−ADR−RES−2
Oxygen uptake (fmol/cell/hr)
Glucose uptake (fmol/cell/hr)

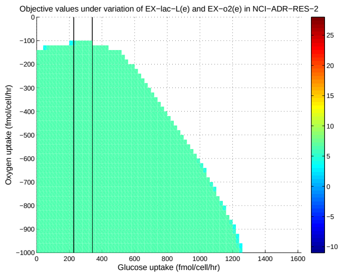
Objective values under variation of EX−lac−L(e) and EX−o2(e) in NCI−ADR−RES−2
Oxygen uptake (fmol/cell/hr)
Glucose uptake (fmol/cell/hr)

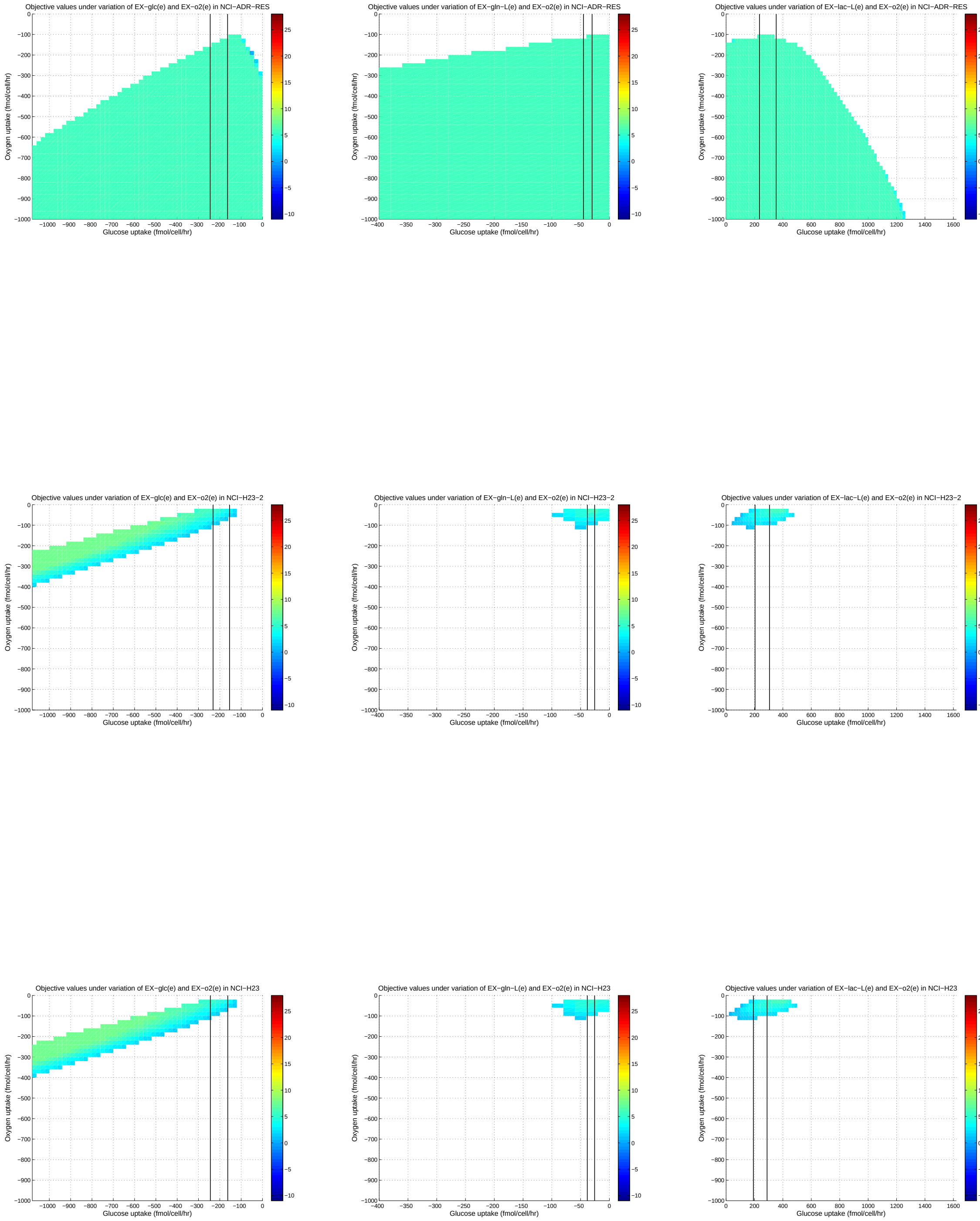
Objective values under variation of EX−glc(e) and EX−o2(e) in NCI−ADR−RES
Objective values under variation of EX−gln−L(e) and EX−o2(e) in NCI−ADR−RES
Objective values under variation of EX−lac−L(e) and EX−o2(e) in NCI−ADR−RES
Objective values under variation of EX−glc(e) and EX−o2(e) in NCI−H23−2
Objective values under variation of EX−gln−L(e) and EX−o2(e) in NCI−H23−2
Objective values under variation of EX−lac−L(e) and EX−o2(e) in NCI−H23−2
Objective values under variation of EX−glc(e) and EX−o2(e) in NCI−H23
Objective values under variation of EX−gln−L(e) and EX−o2(e) in NCI−H23
Objective values under variation of EX−lac−L(e) and EX−o2(e) in NCI−H23
Oxygen uptake (fmol/cell/hr)
Glucose uptake (fmol/cell/hr)

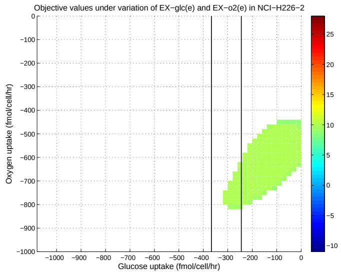
Objective values under variation of EX−glc(e) and EX−o2(e) in NCI−H226−2
Oxygen uptake (fmol/cell/hr)
Glucose uptake (fmol/cell/hr)

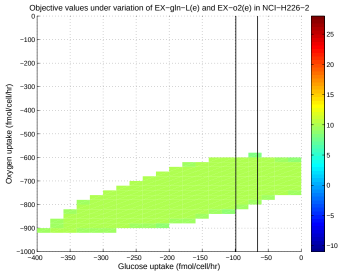
Objective values under variation of EX−gln−L(e) and EX−o2(e) in NCI−H226−2
Oxygen uptake (fmol/cell/hr)
Glucose uptake (fmol/cell/hr)

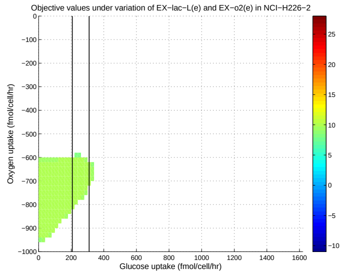
Objective values under variation of EX−lac−L(e) and EX−o2(e) in NCI−H226−2
Oxygen uptake (fmol/cell/hr)
Glucose uptake (fmol/cell/hr)

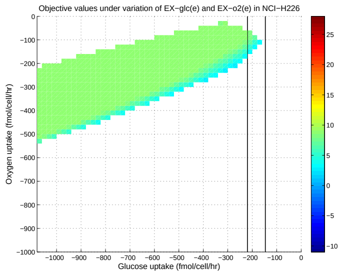
Objective values under variation of EX−glc(e) and EX−o2(e) in NCI−H226
Oxygen uptake (fmol/cell/hr)
Glucose uptake (fmol/cell/hr)

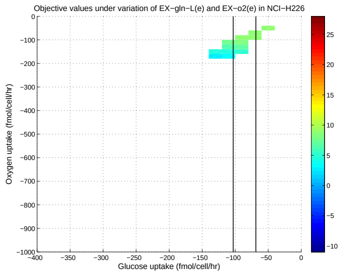
Objective values under variation of EX−gln−L(e) and EX−o2(e) in NCI−H226
Oxygen uptake (fmol/cell/hr)
Glucose uptake (fmol/cell/hr)

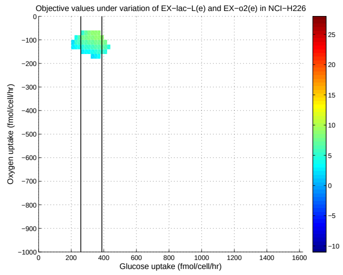
Objective values under variation of EX−lac−L(e) and EX−o2(e) in NCI−H226
Oxygen uptake (fmol/cell/hr)
Glucose uptake (fmol/cell/hr)

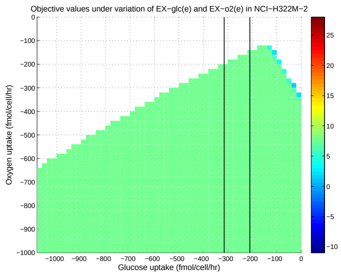
Objective values under variation of EX−glc(e) and EX−o2(e) in NCI−H322M−2
Oxygen uptake (fmol/cell/hr)
Glucose uptake (fmol/cell/hr)

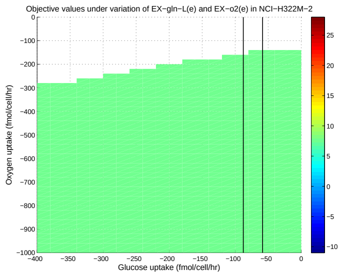
Objective values under variation of EX−gln−L(e) and EX−o2(e) in NCI−H322M−2
Oxygen uptake (fmol/cell/hr)
Glucose uptake (fmol/cell/hr)

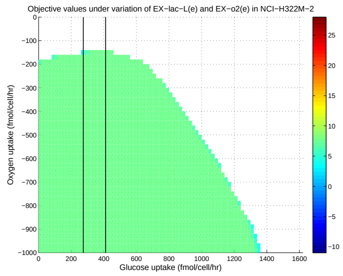
Objective values under variation of EX−lac−L(e) and EX−o2(e) in NCI−H322M−2
Oxygen uptake (fmol/cell/hr)
Glucose uptake (fmol/cell/hr)

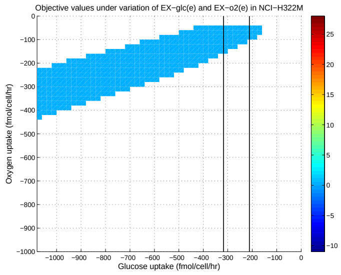
Objective values under variation of EX−glc(e) and EX−o2(e) in NCI−H322M
Oxygen uptake (fmol/cell/hr)
Glucose uptake (fmol/cell/hr)
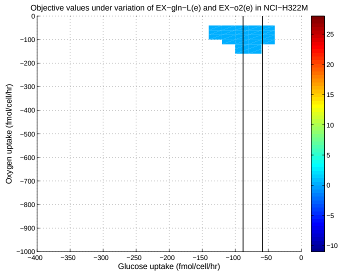
Objective values under variation of EX−gln−L(e) and EX−o2(e) in NCI−H322M
Oxygen uptake (fmol/cell/hr)
Glucose uptake (fmol/cell/hr)
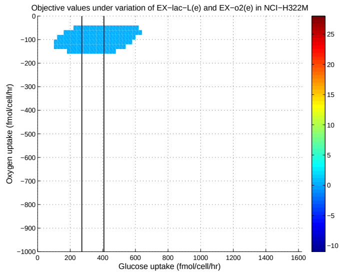
Objective values under variation of EX−lac−L(e) and EX−o2(e) in NCI−H322M
Oxygen uptake (fmol/cell/hr)
Glucose uptake (fmol/cell/hr)
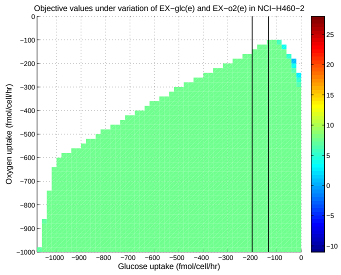
Objective values under variation of EX−glc(e) and EX−o2(e) in NCI−H460−2
Oxygen uptake (fmol/cell/hr)
Glucose uptake (fmol/cell/hr)
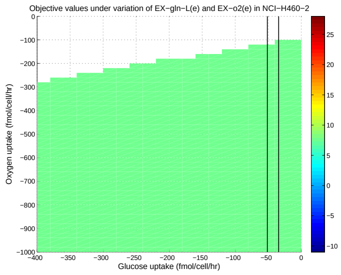
Objective values under variation of EX−gln−L(e) and EX−o2(e) in NCI−H460−2
Oxygen uptake (fmol/cell/hr)
Glucose uptake (fmol/cell/hr)
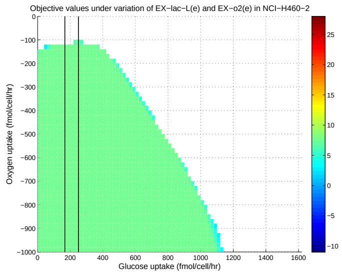
Objective values under variation of EX−lac−L(e) and EX−o2(e) in NCI−H460−2
Oxygen uptake (fmol/cell/hr)
Glucose uptake (fmol/cell/hr)
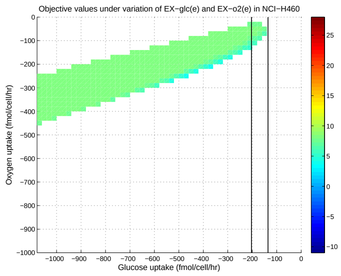
Objective values under variation of EX−glc(e) and EX−o2(e) in NCI−H460
Oxygen uptake (fmol/cell/hr)
Glucose uptake (fmol/cell/hr)
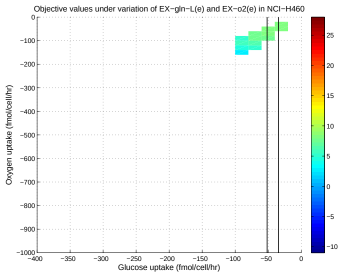
Objective values under variation of EX−gln−L(e) and EX−o2(e) in NCI−H460
Oxygen uptake (fmol/cell/hr)
Glucose uptake (fmol/cell/hr)
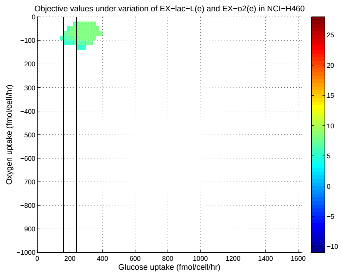
Objective values under variation of EX−lac−L(e) and EX−o2(e) in NCI−H460
Oxygen uptake (fmol/cell/hr)
Glucose uptake (fmol/cell/hr)

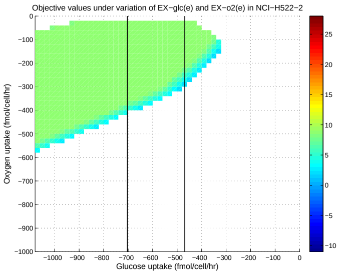
Objective values under variation of EX−glc(e) and EX−o2(e) in NCI−H522−2
Oxygen uptake (fmol/cell/hr)
Glucose uptake (fmol/cell/hr)

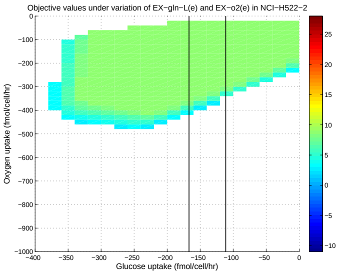
Objective values under variation of EX−gln−L(e) and EX−o2(e) in NCI−H522−2
Oxygen uptake (fmol/cell/hr)
Glucose uptake (fmol/cell/hr)

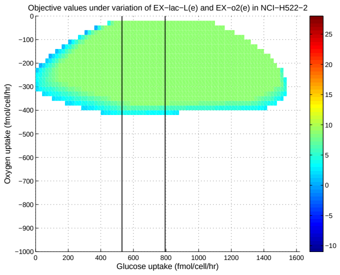
Objective values under variation of EX−lac−L(e) and EX−o2(e) in NCI−H522−2
Oxygen uptake (fmol/cell/hr)
Glucose uptake (fmol/cell/hr)

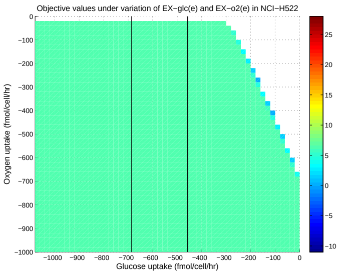
Objective values under variation of EX−glc(e) and EX−o2(e) in NCI−H522
Oxygen uptake (fmol/cell/hr)
Glucose uptake (fmol/cell/hr)

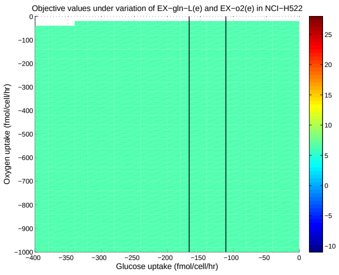
Objective values under variation of EX−gln−L(e) and EX−o2(e) in NCI−H522
Oxygen uptake (fmol/cell/hr)
Glucose uptake (fmol/cell/hr)

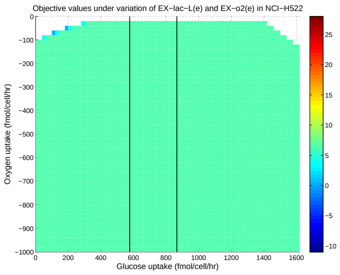
Objective values under variation of EX−lac−L(e) and EX−o2(e) in NCI−H522
Oxygen uptake (fmol/cell/hr)
Glucose uptake (fmol/cell/hr)

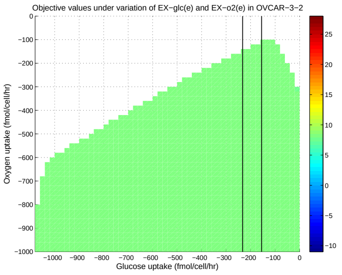
Objective values under variation of EX−glc(e) and EX−o2(e) in OVCAR−3−2
Oxygen uptake (fmol/cell/hr)
Glucose uptake (fmol/cell/hr)

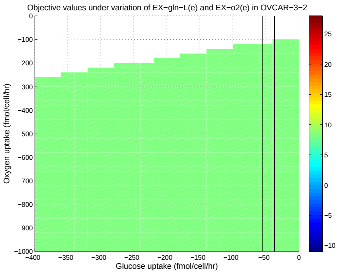
Objective values under variation of EX−gln−L(e) and EX−o2(e) in OVCAR−3−2
Oxygen uptake (fmol/cell/hr)
Glucose uptake (fmol/cell/hr)

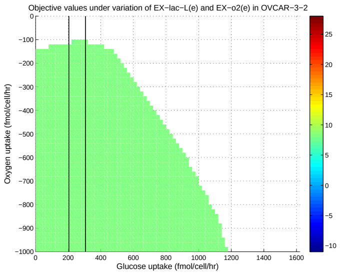
Objective values under variation of EX−lac−L(e) and EX−o2(e) in OVCAR−3−2
Oxygen uptake (fmol/cell/hr)
Glucose uptake (fmol/cell/hr)

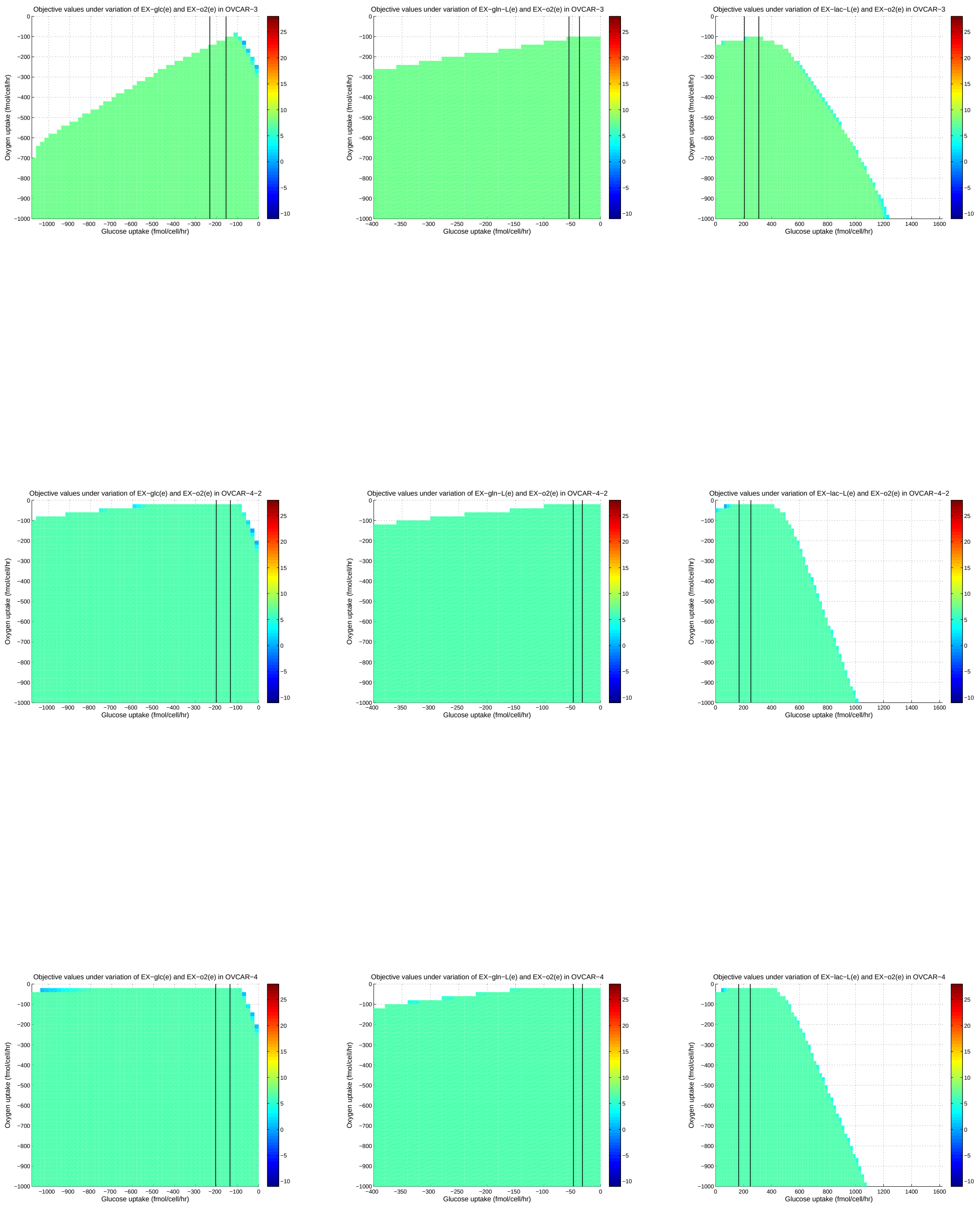
Objective values under variation of EX−glc(e) and EX−o2(e) in OVCAR−3
Objective values under variation of EX−gln−L(e) and EX−o2(e) in OVCAR−3
Objective values under variation of EX−lac−L(e) and EX−o2(e) in OVCAR−3
Objective values under variation of EX−glc(e) and EX−o2(e) in OVCAR−4−2
Objective values under variation of EX−gln−L(e) and EX−o2(e) in OVCAR−4−2
Objective values under variation of EX−lac−L(e) and EX−o2(e) in OVCAR−4−2
Objective values under variation of EX−glc(e) and EX−o2(e) in OVCAR−4
Objective values under variation of EX−gln−L(e) and EX−o2(e) in OVCAR−4
Objective values under variation of EX−lac−L(e) and EX−o2(e) in OVCAR−4
Oxygen uptake (fmol/cell/hr)
Glucose uptake (fmol/cell/hr)

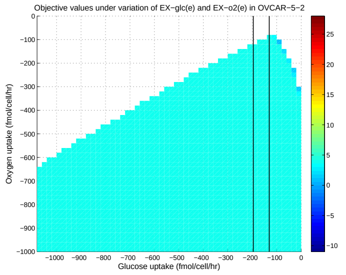
Objective values under variation of EX−glc(e) and EX−o2(e) in OVCAR−5−2
Oxygen uptake (fmol/cell/hr)
Glucose uptake (fmol/cell/hr)

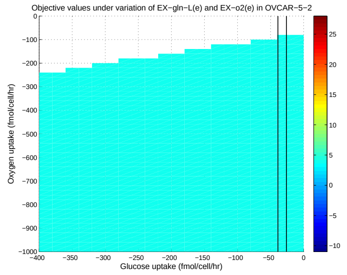
Objective values under variation of EX−gln−L(e) and EX−o2(e) in OVCAR−5−2
Oxygen uptake (fmol/cell/hr)
Glucose uptake (fmol/cell/hr)

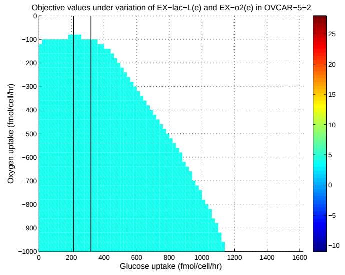
Objective values under variation of EX−lac−L(e) and EX−o2(e) in OVCAR−5−2
Oxygen uptake (fmol/cell/hr)
Glucose uptake (fmol/cell/hr)

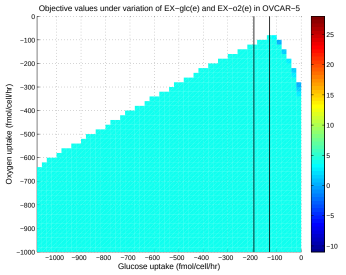
Objective values under variation of EX−glc(e) and EX−o2(e) in OVCAR−5
Oxygen uptake (fmol/cell/hr)
Glucose uptake (fmol/cell/hr)

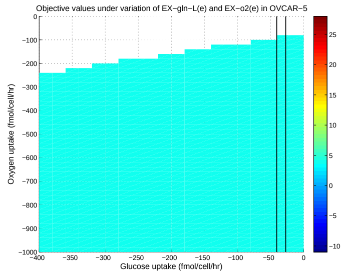
Objective values under variation of EX−gln−L(e) and EX−o2(e) in OVCAR−5
Oxygen uptake (fmol/cell/hr)
Glucose uptake (fmol/cell/hr)

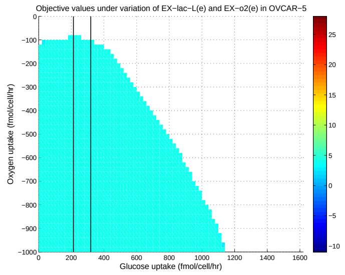
Objective values under variation of EX−lac−L(e) and EX−o2(e) in OVCAR−5
Oxygen uptake (fmol/cell/hr)
Glucose uptake (fmol/cell/hr)

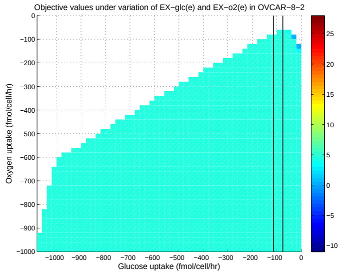
Objective values under variation of EX−glc(e) and EX−o2(e) in OVCAR−8−2
Oxygen uptake (fmol/cell/hr)
Glucose uptake (fmol/cell/hr)

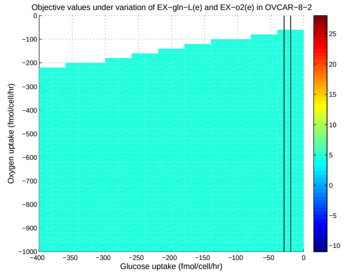
Objective values under variation of EX−gln−L(e) and EX−o2(e) in OVCAR−8−2
Oxygen uptake (fmol/cell/hr)
Glucose uptake (fmol/cell/hr)

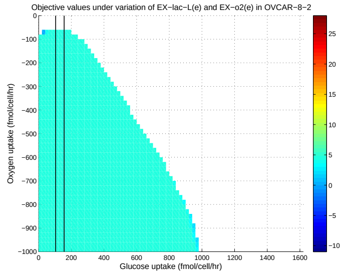
Objective values under variation of EX−lac−L(e) and EX−o2(e) in OVCAR−8−2
Oxygen uptake (fmol/cell/hr)
Glucose uptake (fmol/cell/hr)

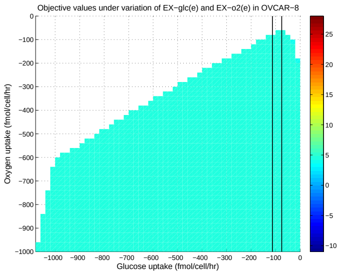
Objective values under variation of EX−glc(e) and EX−o2(e) in OVCAR−8
Oxygen uptake (fmol/cell/hr)
Glucose uptake (fmol/cell/hr)
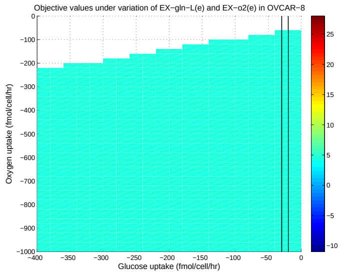
Objective values under variation of EX−gln−L(e) and EX−o2(e) in OVCAR−8
Oxygen uptake (fmol/cell/hr)
Glucose uptake (fmol/cell/hr)
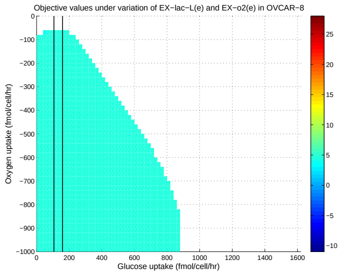
Objective values under variation of EX−lac−L(e) and EX−o2(e) in OVCAR−8
Oxygen uptake (fmol/cell/hr)
Glucose uptake (fmol/cell/hr)
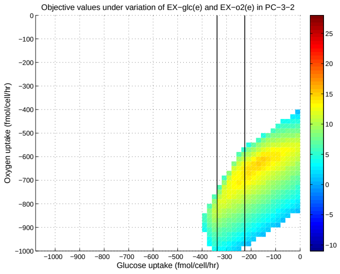
Objective values under variation of EX−glc(e) and EX−o2(e) in PC−3−2
Oxygen uptake (fmol/cell/hr)
Glucose uptake (fmol/cell/hr)
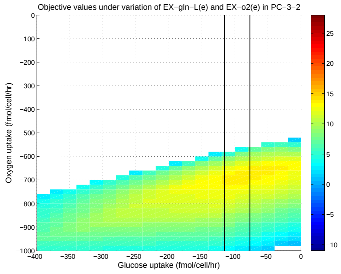
Objective values under variation of EX−gln−L(e) and EX−o2(e) in PC−3−2
Oxygen uptake (fmol/cell/hr)
Glucose uptake (fmol/cell/hr)
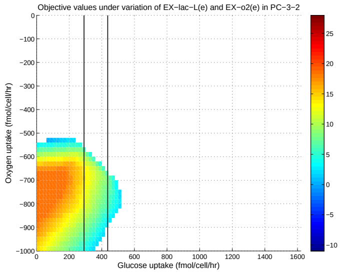
Objective values under variation of EX−lac−L(e) and EX−o2(e) in PC−3−2
Oxygen uptake (fmol/cell/hr)
Glucose uptake (fmol/cell/hr)
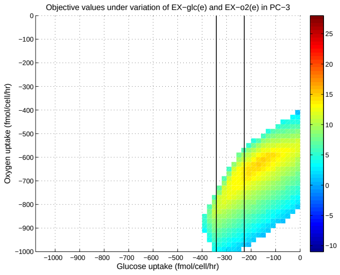
Objective values under variation of EX−glc(e) and EX−o2(e) in PC−3
Oxygen uptake (fmol/cell/hr)
Glucose uptake (fmol/cell/hr)
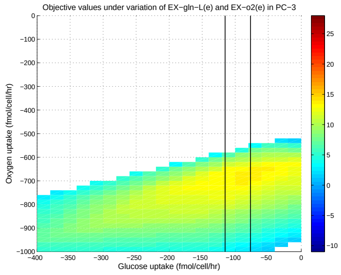
Objective values under variation of EX−gln−L(e) and EX−o2(e) in PC−3
Oxygen uptake (fmol/cell/hr)
Glucose uptake (fmol/cell/hr)
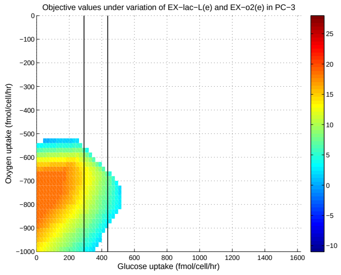
Objective values under variation of EX−lac−L(e) and EX−o2(e) in PC−3
Oxygen uptake (fmol/cell/hr)
Glucose uptake (fmol/cell/hr)

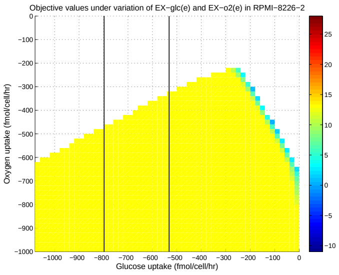
Objective values under variation of EX−glc(e) and EX−o2(e) in RPMI−8226−2
Oxygen uptake (fmol/cell/hr)
Glucose uptake (fmol/cell/hr)

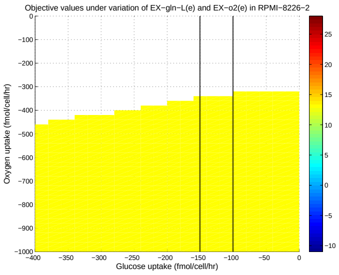
Objective values under variation of EX−gln−L(e) and EX−o2(e) in RPMI−8226−2
Oxygen uptake (fmol/cell/hr)
Glucose uptake (fmol/cell/hr)

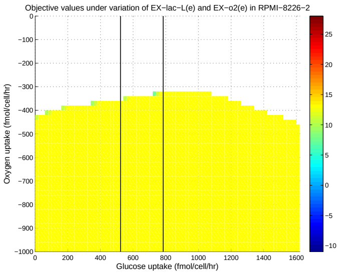
Objective values under variation of EX−lac−L(e) and EX−o2(e) in RPMI−8226−2
Oxygen uptake (fmol/cell/hr)
Glucose uptake (fmol/cell/hr)

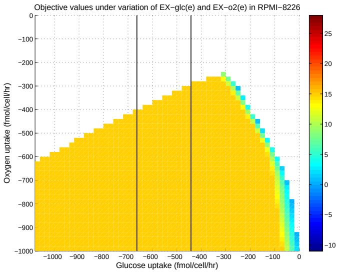
Objective values under variation of EX−glc(e) and EX−o2(e) in RPMI−8226
Oxygen uptake (fmol/cell/hr)
Glucose uptake (fmol/cell/hr)

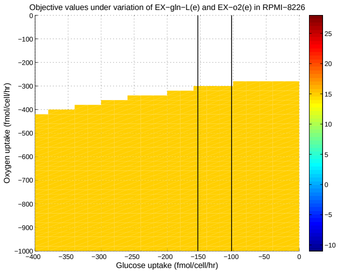
Objective values under variation of EX−gln−L(e) and EX−o2(e) in RPMI−8226
Oxygen uptake (fmol/cell/hr)
Glucose uptake (fmol/cell/hr)

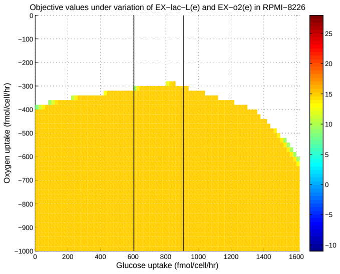
Objective values under variation of EX−lac−L(e) and EX−o2(e) in RPMI−8226
Oxygen uptake (fmol/cell/hr)
Glucose uptake (fmol/cell/hr)

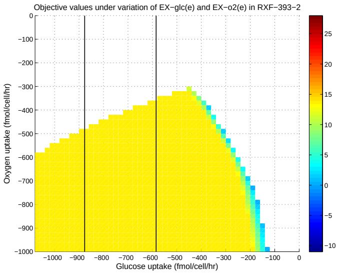
Objective values under variation of EX−glc(e) and EX−o2(e) in RXF−393−2
Oxygen uptake (fmol/cell/hr)
Glucose uptake (fmol/cell/hr)

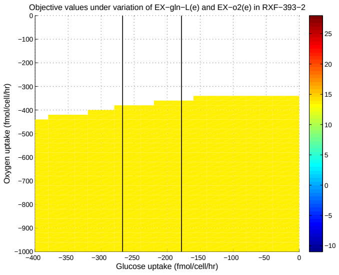
Objective values under variation of EX−gln−L(e) and EX−o2(e) in RXF−393−2
Oxygen uptake (fmol/cell/hr)
Glucose uptake (fmol/cell/hr)

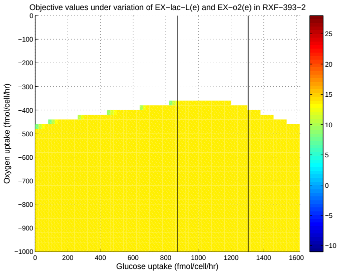
Objective values under variation of EX−lac−L(e) and EX−o2(e) in RXF−393−2
Oxygen uptake (fmol/cell/hr)
Glucose uptake (fmol/cell/hr)

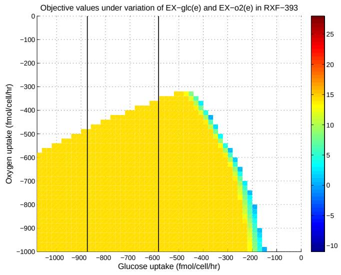
Objective values under variation of EX−glc(e) and EX−o2(e) in RXF−393
Oxygen uptake (fmol/cell/hr)
Glucose uptake (fmol/cell/hr)

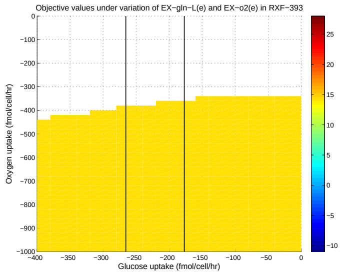
Objective values under variation of EX−gln−L(e) and EX−o2(e) in RXF−393
Oxygen uptake (fmol/cell/hr)
Glucose uptake (fmol/cell/hr)

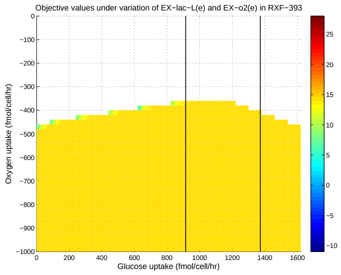
Objective values under variation of EX−lac−L(e) and EX−o2(e) in RXF−393
Oxygen uptake (fmol/cell/hr)
Glucose uptake (fmol/cell/hr)

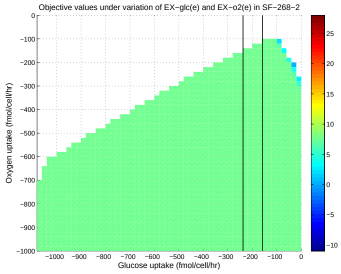
Objective values under variation of EX−glc(e) and EX−o2(e) in SF−268−2
Oxygen uptake (fmol/cell/hr)
Glucose uptake (fmol/cell/hr)

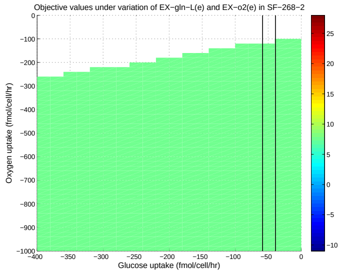
Objective values under variation of EX−gln−L(e) and EX−o2(e) in SF−268−2
Oxygen uptake (fmol/cell/hr)
Glucose uptake (fmol/cell/hr)

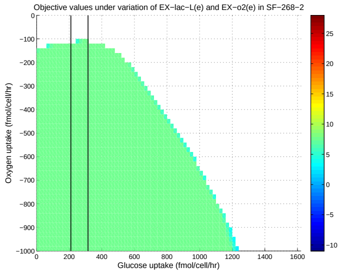
Objective values under variation of EX−lac−L(e) and EX−o2(e) in SF−268−2
Oxygen uptake (fmol/cell/hr)
Glucose uptake (fmol/cell/hr)

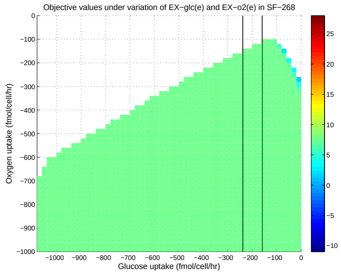
Objective values under variation of EX−glc(e) and EX−o2(e) in SF−268
Oxygen uptake (fmol/cell/hr)
Glucose uptake (fmol/cell/hr)

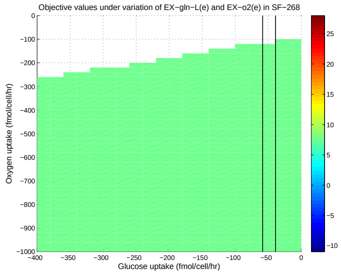
Objective values under variation of EX−gln−L(e) and EX−o2(e) in SF−268
Oxygen uptake (fmol/cell/hr)
Glucose uptake (fmol/cell/hr)

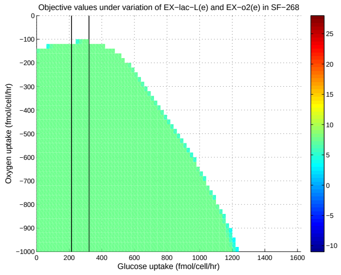
Objective values under variation of EX−lac−L(e) and EX−o2(e) in SF−268
Oxygen uptake (fmol/cell/hr)
Glucose uptake (fmol/cell/hr)

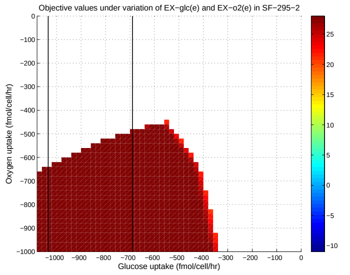
Objective values under variation of EX−glc(e) and EX−o2(e) in SF−295−2
Oxygen uptake (fmol/cell/hr)
Glucose uptake (fmol/cell/hr)

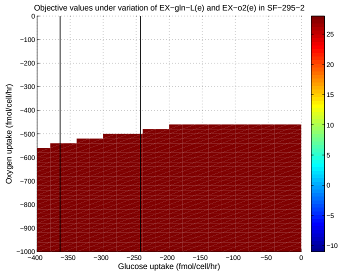
Objective values under variation of EX−gln−L(e) and EX−o2(e) in SF−295−2
Oxygen uptake (fmol/cell/hr)
Glucose uptake (fmol/cell/hr)

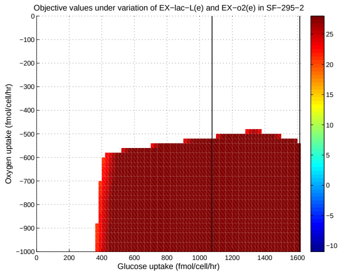
Objective values under variation of EX−lac−L(e) and EX−o2(e) in SF−295−2
Oxygen uptake (fmol/cell/hr)
Glucose uptake (fmol/cell/hr)

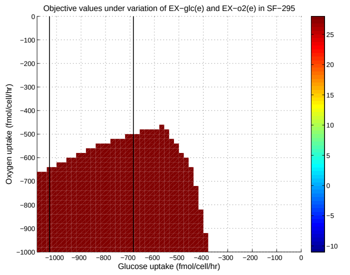
Objective values under variation of EX−glc(e) and EX−o2(e) in SF−295
Oxygen uptake (fmol/cell/hr)
Glucose uptake (fmol/cell/hr)

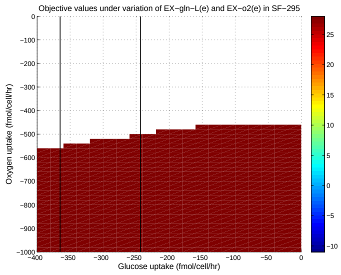
Objective values under variation of EX−gln−L(e) and EX−o2(e) in SF−295
Oxygen uptake (fmol/cell/hr)
Glucose uptake (fmol/cell/hr)

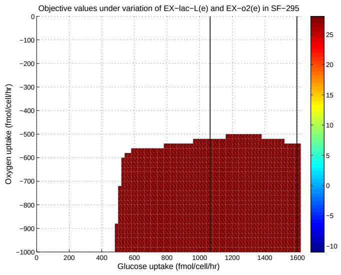
Objective values under variation of EX−lac−L(e) and EX−o2(e) in SF−295
Oxygen uptake (fmol/cell/hr)
Glucose uptake (fmol/cell/hr)

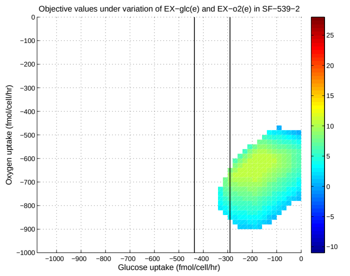
Objective values under variation of EX−glc(e) and EX−o2(e) in SF−539−2
Oxygen uptake (fmol/cell/hr)
Glucose uptake (fmol/cell/hr)

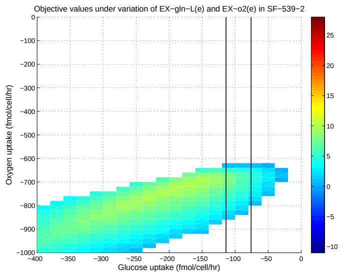
Objective values under variation of EX−gln−L(e) and EX−o2(e) in SF−539−2
Oxygen uptake (fmol/cell/hr)
Glucose uptake (fmol/cell/hr)

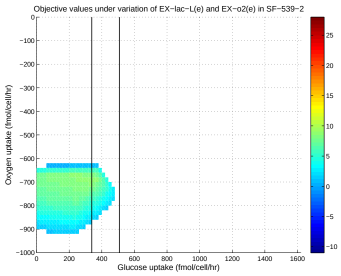
Objective values under variation of EX−lac−L(e) and EX−o2(e) in SF−539−2
Oxygen uptake (fmol/cell/hr)
Glucose uptake (fmol/cell/hr)

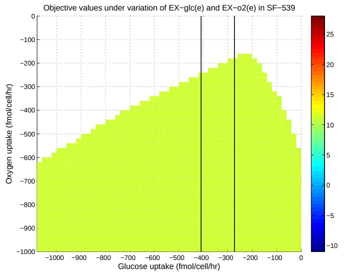
Objective values under variation of EX−glc(e) and EX−o2(e) in SF−539
Oxygen uptake (fmol/cell/hr)
Glucose uptake (fmol/cell/hr)

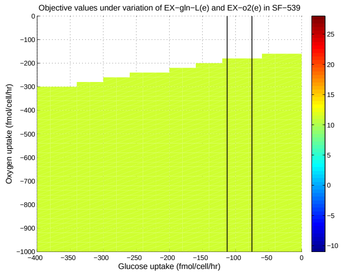
Objective values under variation of EX−gln−L(e) and EX−o2(e) in SF−539
Oxygen uptake (fmol/cell/hr)
Glucose uptake (fmol/cell/hr)

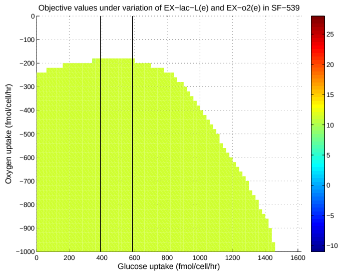
Objective values under variation of EX−lac−L(e) and EX−o2(e) in SF−539
Oxygen uptake (fmol/cell/hr)
Glucose uptake (fmol/cell/hr)

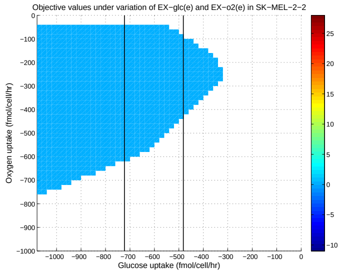
Objective values under variation of EX−glc(e) and EX−o2(e) in SK−MEL−2−2
Oxygen uptake (fmol/cell/hr)
Glucose uptake (fmol/cell/hr)

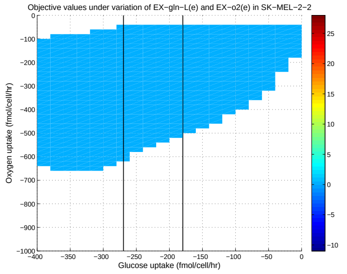
Objective values under variation of EX−gln−L(e) and EX−o2(e) in SK−MEL−2−2
Oxygen uptake (fmol/cell/hr)
Glucose uptake (fmol/cell/hr)

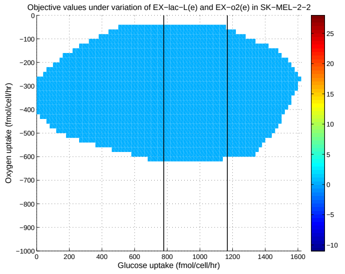
Objective values under variation of EX−lac−L(e) and EX−o2(e) in SK−MEL−2−2
Oxygen uptake (fmol/cell/hr)
Glucose uptake (fmol/cell/hr)

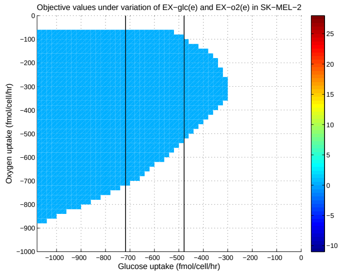
Objective values under variation of EX−glc(e) and EX−o2(e) in SK−MEL−2
Oxygen uptake (fmol/cell/hr)
Glucose uptake (fmol/cell/hr)

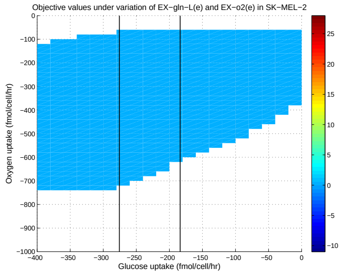
Objective values under variation of EX−gln−L(e) and EX−o2(e) in SK−MEL−2
Oxygen uptake (fmol/cell/hr)
Glucose uptake (fmol/cell/hr)

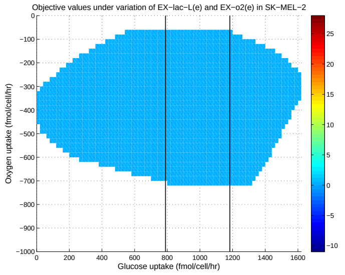
Objective values under variation of EX−lac−L(e) and EX−o2(e) in SK−MEL−2
Oxygen uptake (fmol/cell/hr)
Glucose uptake (fmol/cell/hr)

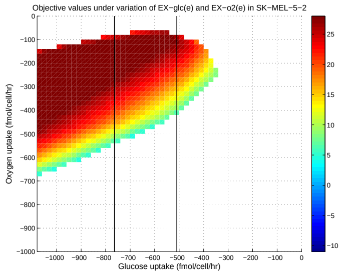
Objective values under variation of EX−glc(e) and EX−o2(e) in SK−MEL−5−2
Oxygen uptake (fmol/cell/hr)
Glucose uptake (fmol/cell/hr)
0 −100 −200 −300 −400 −500 −600 −700 −800 −900 −1000
−1000 −900 −800 −700 −600 −500 −400 −300 −200 −100 0
25 20 15 10 5 0 −5 −10

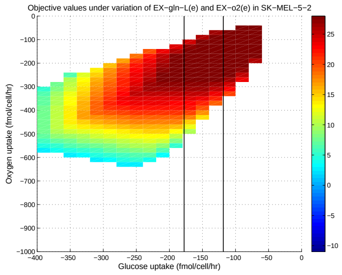
Objective values under variation of EX−gln−L(e) and EX−o2(e) in SK−MEL−5−2
Oxygen uptake (fmol/cell/hr)
Glucose uptake (fmol/cell/hr)
0 −100 −200 −300 −400 −500 −600 −700 −800 −900 −1000
−400 −350 −300 −250 −200 −150 −100 −50 0
25 20 15 10 5 0 −5 −10

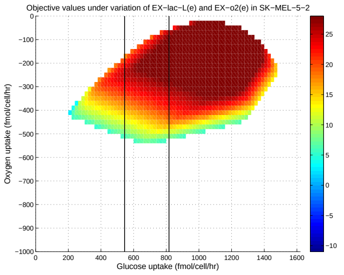
Objective values under variation of EX−lac−L(e) and EX−o2(e) in SK−MEL−5−2
Oxygen uptake (fmol/cell/hr)
Glucose uptake (fmol/cell/hr)
0 −100 −200 −300 −400 −500 −600 −700 −800 −900 −1000
0 200 400 600 800 1000 1200 1400 1600
25 20 15 10 5 0 −5 −10

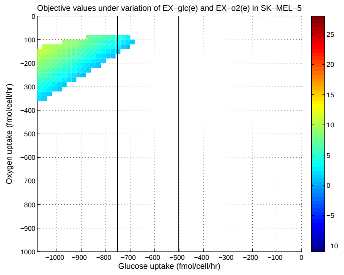
Objective values under variation of EX−glc(e) and EX−o2(e) in SK−MEL−5
Oxygen uptake (fmol/cell/hr)
Glucose uptake (fmol/cell/hr)
0 −100 −200 −300 −400 −500 −600 −700 −800 −900 −1000
−1000 −900 −800 −700 −600 −500 −400 −300 −200 −100 0
25 20 15 10 5 0 −5 −10

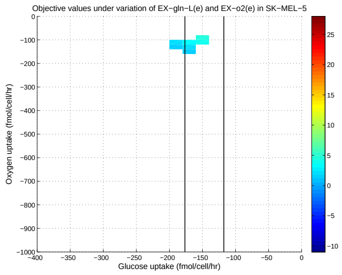
Objective values under variation of EX−gln−L(e) and EX−o2(e) in SK−MEL−5
Oxygen uptake (fmol/cell/hr)
Glucose uptake (fmol/cell/hr)
0 −100 −200 −300 −400 −500 −600 −700 −800 −900 −1000
−400 −350 −300 −250 −200 −150 −100 −50 0
25 20 15 10 5 0 −5 −10

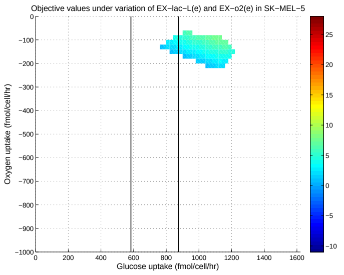
Objective values under variation of EX−lac−L(e) and EX−o2(e) in SK−MEL−5
Oxygen uptake (fmol/cell/hr)
Glucose uptake (fmol/cell/hr)
0 −100 −200 −300 −400 −500 −600 −700 −800 −900 −1000
0 200 400 600 800 1000 1200 1400 1600
25 20 15 10 5 0 −5 −10

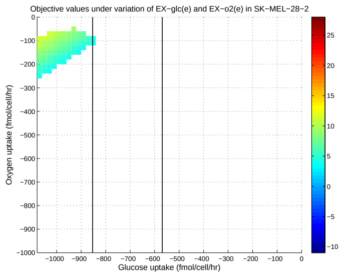
Objective values under variation of EX−glc(e) and EX−o2(e) in SK−MEL−28−2
Oxygen uptake (fmol/cell/hr)
Glucose uptake (fmol/cell/hr)
0 −100 −200 −300 −400 −500 −600 −700 −800 −900 −1000
−1000 −900 −800 −700 −600 −500 −400 −300 −200 −100 0
25 20 15 10 5 0 −5 −10

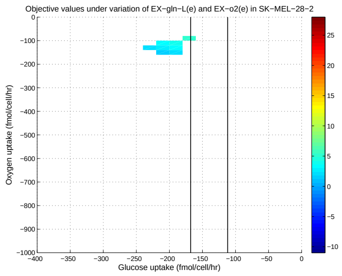
Objective values under variation of EX−gln−L(e) and EX−o2(e) in SK−MEL−28−2
Oxygen uptake (fmol/cell/hr)
Glucose uptake (fmol/cell/hr)
0 −100 −200 −300 −400 −500 −600 −700 −800 −900 −1000
−400 −350 −300 −250 −200 −150 −100 −50 0
25 20 15 10 5 0 −5 −10

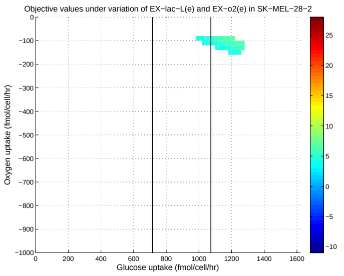
Objective values under variation of EX−lac−L(e) and EX−o2(e) in SK−MEL−28−2
Oxygen uptake (fmol/cell/hr)
Glucose uptake (fmol/cell/hr)
0 −100 −200 −300 −400 −500 −600 −700 −800 −900 −1000
0 200 400 600 800 1000 1200 1400 1600
25 20 15 10 5 0 −5 −10

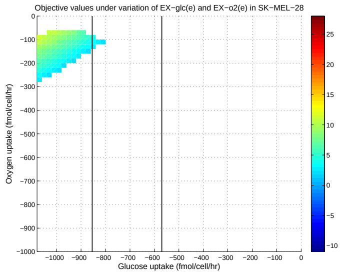
Objective values under variation of EX−glc(e) and EX−o2(e) in SK−MEL−28
Oxygen uptake (fmol/cell/hr)
Glucose uptake (fmol/cell/hr)

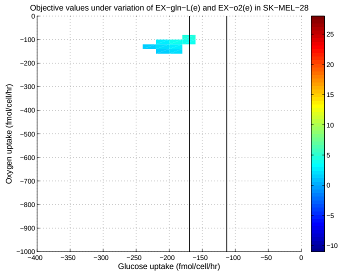
Objective values under variation of EX−gln−L(e) and EX−o2(e) in SK−MEL−28
Oxygen uptake (fmol/cell/hr)
Glucose uptake (fmol/cell/hr)

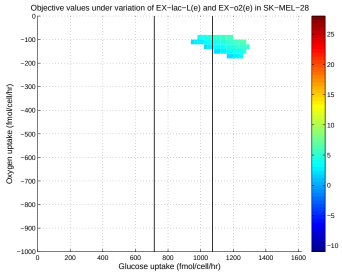
Objective values under variation of EX−lac−L(e) and EX−o2(e) in SK−MEL−28
Oxygen uptake (fmol/cell/hr)
Glucose uptake (fmol/cell/hr)

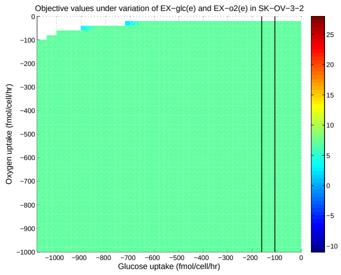
Objective values under variation of EX−glc(e) and EX−o2(e) in SK−OV−3−2
Oxygen uptake (fmol/cell/hr)
Glucose uptake (fmol/cell/hr)

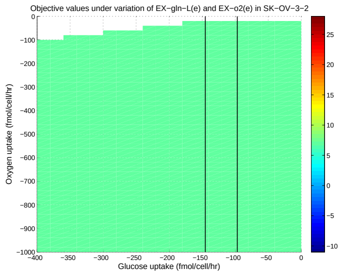
Objective values under variation of EX−gln−L(e) and EX−o2(e) in SK−OV−3−2
Oxygen uptake (fmol/cell/hr)
Glucose uptake (fmol/cell/hr)

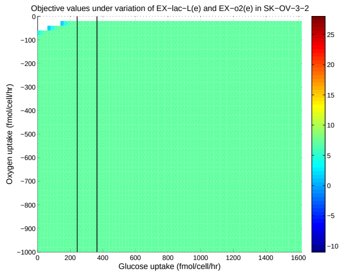
Objective values under variation of EX−lac−L(e) and EX−o2(e) in SK−OV−3−2
Oxygen uptake (fmol/cell/hr)
Glucose uptake (fmol/cell/hr)

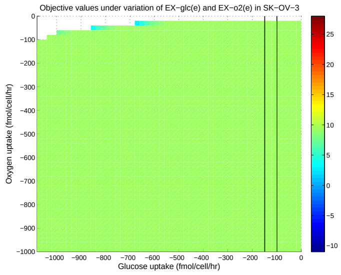
Objective values under variation of EX−glc(e) and EX−o2(e) in SK−OV−3
Oxygen uptake (fmol/cell/hr)
Glucose uptake (fmol/cell/hr)

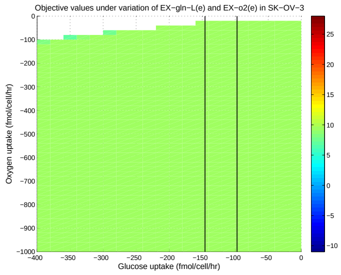
Objective values under variation of EX−gln−L(e) and EX−o2(e) in SK−OV−3
Oxygen uptake (fmol/cell/hr)
Glucose uptake (fmol/cell/hr)

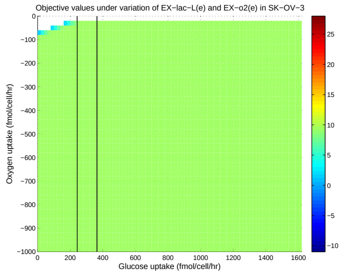
Objective values under variation of EX−lac−L(e) and EX−o2(e) in SK−OV−3
Oxygen uptake (fmol/cell/hr)
Glucose uptake (fmol/cell/hr)

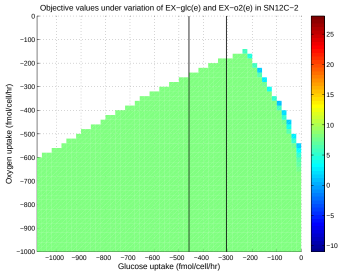
Objective values under variation of EX−glc(e) and EX−o2(e) in SN12C−2
Oxygen uptake (fmol/cell/hr)
Glucose uptake (fmol/cell/hr)

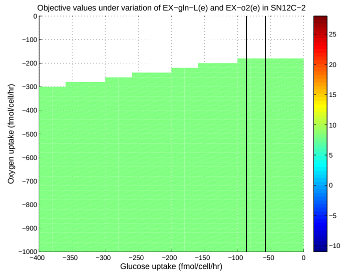
Objective values under variation of EX−gln−L(e) and EX−o2(e) in SN12C−2
Oxygen uptake (fmol/cell/hr)
Glucose uptake (fmol/cell/hr)

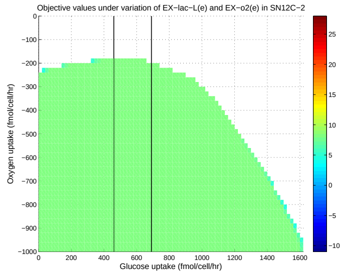
Objective values under variation of EX−lac−L(e) and EX−o2(e) in SN12C−2
Oxygen uptake (fmol/cell/hr)
Glucose uptake (fmol/cell/hr)

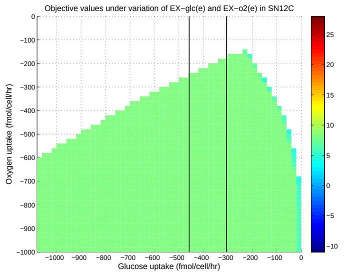
Objective values under variation of EX−glc(e) and EX−o2(e) in SN12C
Oxygen uptake (fmol/cell/hr)
Glucose uptake (fmol/cell/hr)

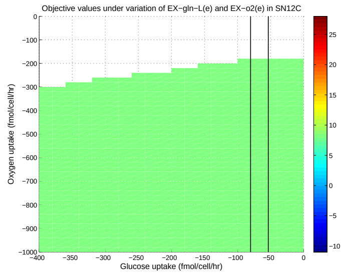
Objective values under variation of EX−gln−L(e) and EX−o2(e) in SN12C
Oxygen uptake (fmol/cell/hr)
Glucose uptake (fmol/cell/hr)

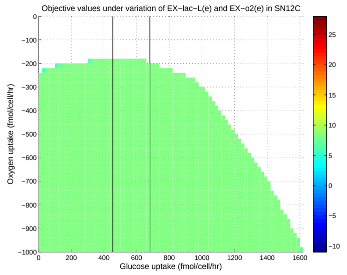
Objective values under variation of EX−lac−L(e) and EX−o2(e) in SN12C
Oxygen uptake (fmol/cell/hr)
Glucose uptake (fmol/cell/hr)

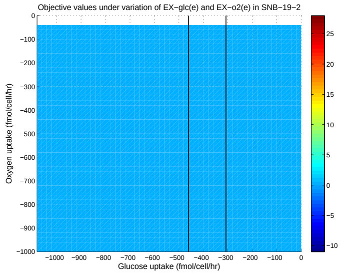
Objective values under variation of EX−glc(e) and EX−o2(e) in SNB−19−2
Oxygen uptake (fmol/cell/hr)
Glucose uptake (fmol/cell/hr)

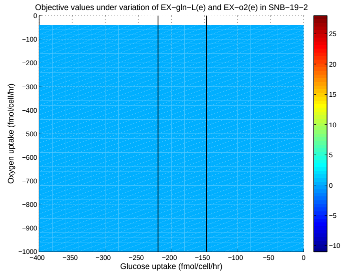
Objective values under variation of EX−gln−L(e) and EX−o2(e) in SNB−19−2
Oxygen uptake (fmol/cell/hr)
Glucose uptake (fmol/cell/hr)

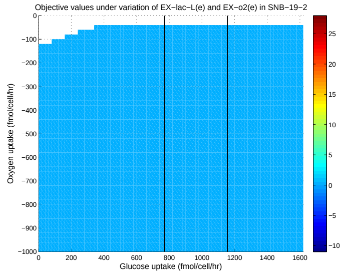
Objective values under variation of EX−lac−L(e) and EX−o2(e) in SNB−19−2
Oxygen uptake (fmol/cell/hr)
Glucose uptake (fmol/cell/hr)

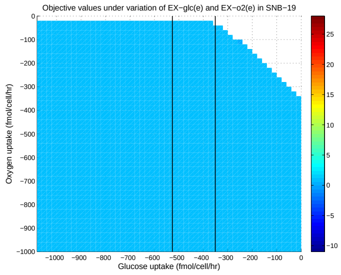
Objective values under variation of EX−glc(e) and EX−o2(e) in SNB−19
Oxygen uptake (fmol/cell/hr)
Glucose uptake (fmol/cell/hr)

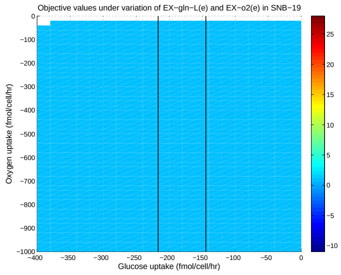
Objective values under variation of EX−gln−L(e) and EX−o2(e) in SNB−19
Oxygen uptake (fmol/cell/hr)
Glucose uptake (fmol/cell/hr)

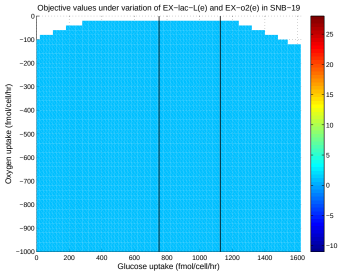
Objective values under variation of EX−lac−L(e) and EX−o2(e) in SNB−19
Oxygen uptake (fmol/cell/hr)
Glucose uptake (fmol/cell/hr)

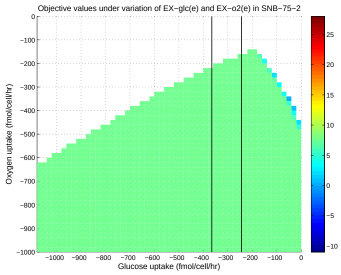
Objective values under variation of EX−glc(e) and EX−o2(e) in SNB−75−2
Oxygen uptake (fmol/cell/hr)
Glucose uptake (fmol/cell/hr)

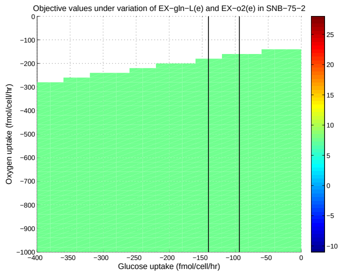
Objective values under variation of EX−gln−L(e) and EX−o2(e) in SNB−75−2
Oxygen uptake (fmol/cell/hr)
Glucose uptake (fmol/cell/hr)

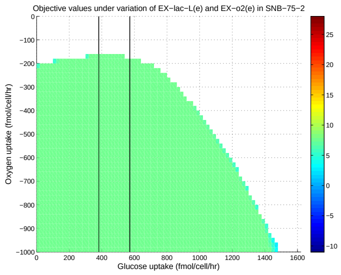
Objective values under variation of EX−lac−L(e) and EX−o2(e) in SNB−75−2
Oxygen uptake (fmol/cell/hr)
Glucose uptake (fmol/cell/hr)

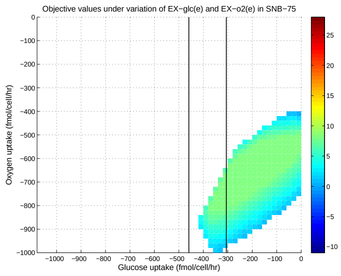
Objective values under variation of EX−glc(e) and EX−o2(e) in SNB−75
Oxygen uptake (fmol/cell/hr)
Glucose uptake (fmol/cell/hr)

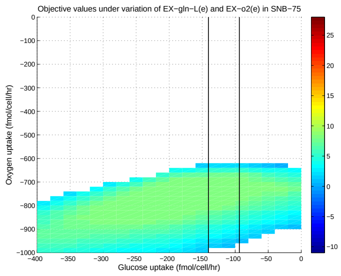
Objective values under variation of EX−gln−L(e) and EX−o2(e) in SNB−75
Oxygen uptake (fmol/cell/hr)
Glucose uptake (fmol/cell/hr)

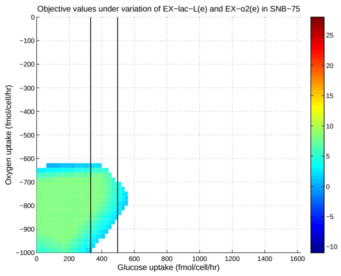
Objective values under variation of EX−lac−L(e) and EX−o2(e) in SNB−75
Oxygen uptake (fmol/cell/hr)
Glucose uptake (fmol/cell/hr)

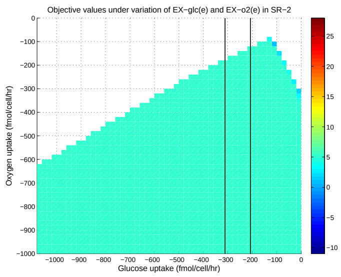
Objective values under variation of EX−glc(e) and EX−o2(e) in SR−2
Oxygen uptake (fmol/cell/hr)
Glucose uptake (fmol/cell/hr)

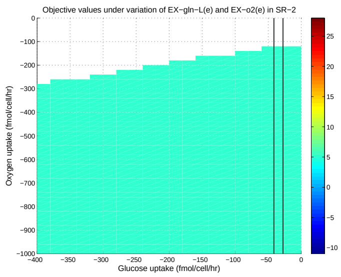
Objective values under variation of EX−gln−L(e) and EX−o2(e) in SR−2
Oxygen uptake (fmol/cell/hr)
Glucose uptake (fmol/cell/hr)

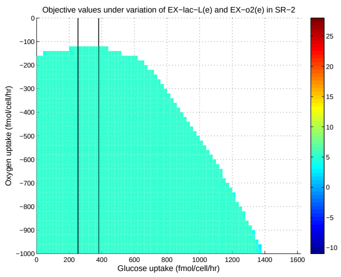
Objective values under variation of EX−lac−L(e) and EX−o2(e) in SR−2
Oxygen uptake (fmol/cell/hr)
Glucose uptake (fmol/cell/hr)

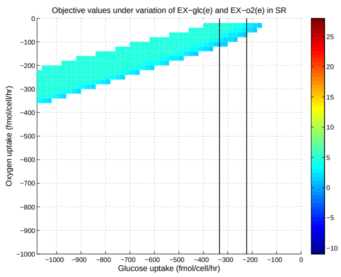
Objective values under variation of EX−glc(e) and EX−o2(e) in SR
Oxygen uptake (fmol/cell/hr)
Glucose uptake (fmol/cell/hr)

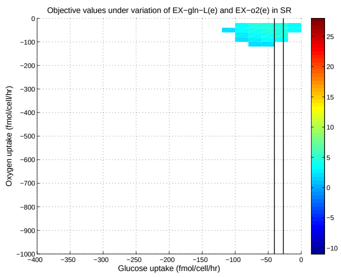
Objective values under variation of EX−gln−L(e) and EX−o2(e) in SR
Oxygen uptake (fmol/cell/hr)
Glucose uptake (fmol/cell/hr)

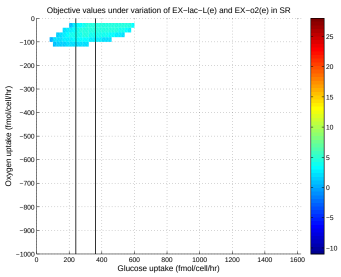
Objective values under variation of EX−lac−L(e) and EX−o2(e) in SR
Oxygen uptake (fmol/cell/hr)
Glucose uptake (fmol/cell/hr)

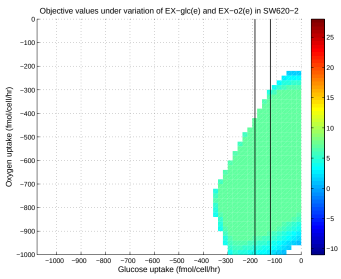
Objective values under variation of EX−glc(e) and EX−o2(e) in SW620−2
Oxygen uptake (fmol/cell/hr)
Glucose uptake (fmol/cell/hr)

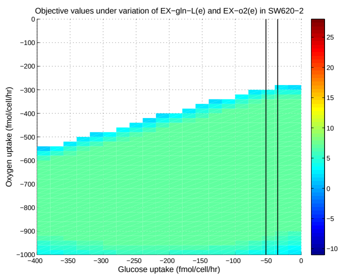
Objective values under variation of EX−gln−L(e) and EX−o2(e) in SW620−2
Oxygen uptake (fmol/cell/hr)
Glucose uptake (fmol/cell/hr)

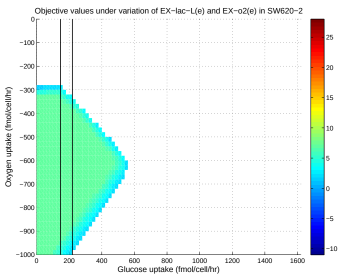
Objective values under variation of EX−lac−L(e) and EX−o2(e) in SW620−2
Oxygen uptake (fmol/cell/hr)
Glucose uptake (fmol/cell/hr)

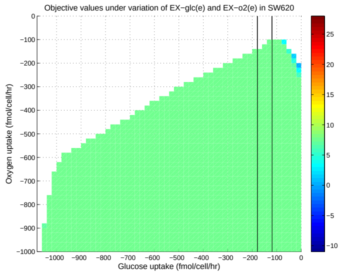
Objective values under variation of EX−glc(e) and EX−o2(e) in SW620
Oxygen uptake (fmol/cell/hr)
Glucose uptake (fmol/cell/hr)

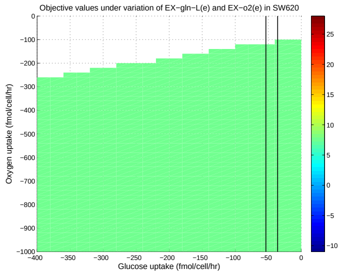
Objective values under variation of EX−gln−L(e) and EX−o2(e) in SW620
Oxygen uptake (fmol/cell/hr)
Glucose uptake (fmol/cell/hr)

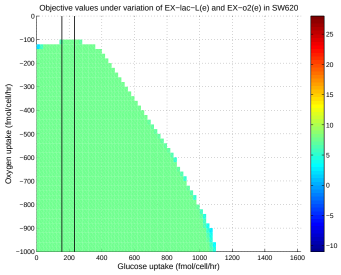
Objective values under variation of EX−lac−L(e) and EX−o2(e) in SW620
Oxygen uptake (fmol/cell/hr)
Glucose uptake (fmol/cell/hr)

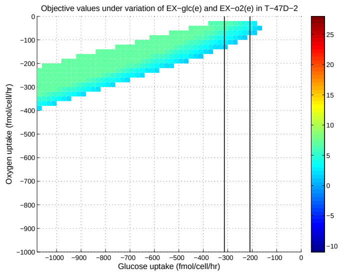
Objective values under variation of EX−glc(e) and EX−o2(e) in T−47D−2
Oxygen uptake (fmol/cell/hr)
Glucose uptake (fmol/cell/hr)

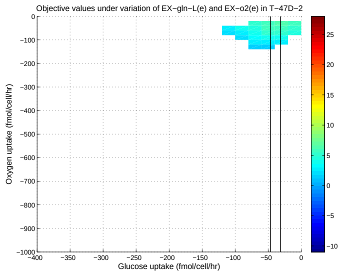
Objective values under variation of EX−gln−L(e) and EX−o2(e) in T−47D−2
Oxygen uptake (fmol/cell/hr)
Glucose uptake (fmol/cell/hr)

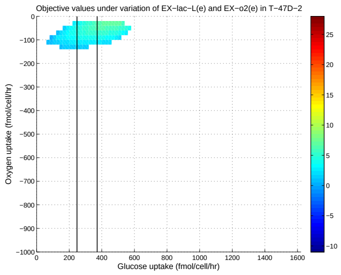
Objective values under variation of EX−lac−L(e) and EX−o2(e) in T−47D−2
Oxygen uptake (fmol/cell/hr)
Glucose uptake (fmol/cell/hr)

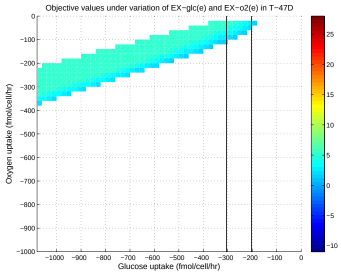
Objective values under variation of EX−glc(e) and EX−o2(e) in T−47D
Oxygen uptake (fmol/cell/hr)
Glucose uptake (fmol/cell/hr)

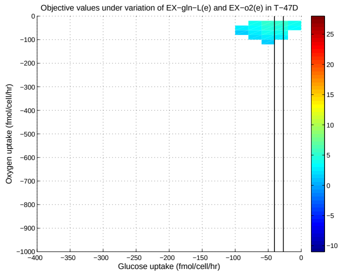
Objective values under variation of EX−gln−L(e) and EX−o2(e) in T−47D
Oxygen uptake (fmol/cell/hr)
Glucose uptake (fmol/cell/hr)

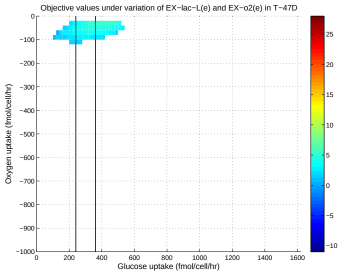
Objective values under variation of EX−lac−L(e) and EX−o2(e) in T−47D
Oxygen uptake (fmol/cell/hr)
Glucose uptake (fmol/cell/hr)

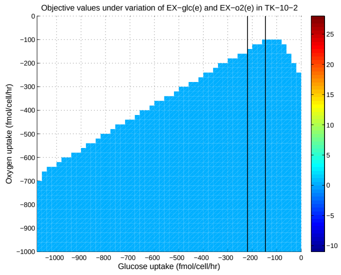
Objective values under variation of EX−glc(e) and EX−o2(e) in TK−10−2
Oxygen uptake (fmol/cell/hr)
Glucose uptake (fmol/cell/hr)

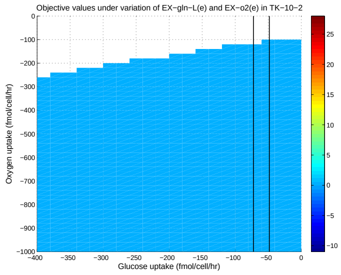
Objective values under variation of EX−gln−L(e) and EX−o2(e) in TK−10−2
Oxygen uptake (fmol/cell/hr)
Glucose uptake (fmol/cell/hr)

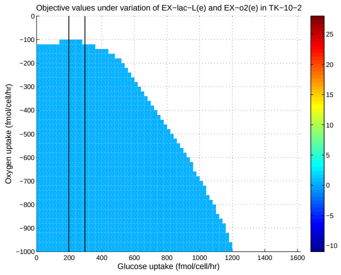
Objective values under variation of EX−lac−L(e) and EX−o2(e) in TK−10−2
Oxygen uptake (fmol/cell/hr)
Glucose uptake (fmol/cell/hr)

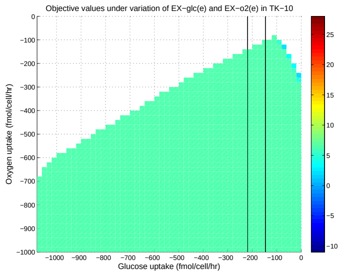
Objective values under variation of EX−glc(e) and EX−o2(e) in TK−10
Oxygen uptake (fmol/cell/hr)
Glucose uptake (fmol/cell/hr)

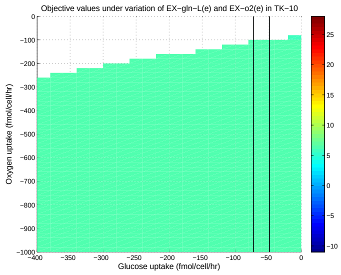
Objective values under variation of EX−gln−L(e) and EX−o2(e) in TK−10
Oxygen uptake (fmol/cell/hr)
Glucose uptake (fmol/cell/hr)

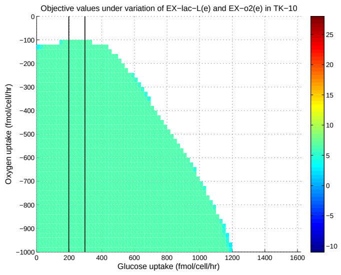
Objective values under variation of EX−lac−L(e) and EX−o2(e) in TK−10
Oxygen uptake (fmol/cell/hr)
Glucose uptake (fmol/cell/hr)

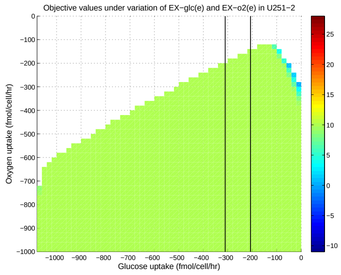
Objective values under variation of EX−glc(e) and EX−o2(e) in U251−2
Oxygen uptake (fmol/cell/hr)
Glucose uptake (fmol/cell/hr)

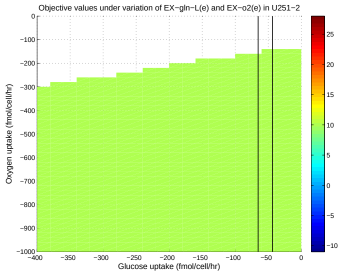
Objective values under variation of EX−gln−L(e) and EX−o2(e) in U251−2
Oxygen uptake (fmol/cell/hr)
Glucose uptake (fmol/cell/hr)

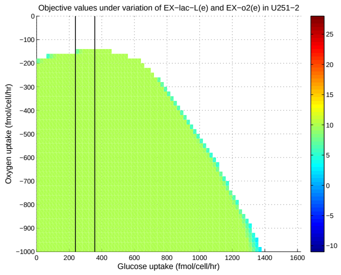
Objective values under variation of EX−lac−L(e) and EX−o2(e) in U251−2
Oxygen uptake (fmol/cell/hr)
Glucose uptake (fmol/cell/hr)

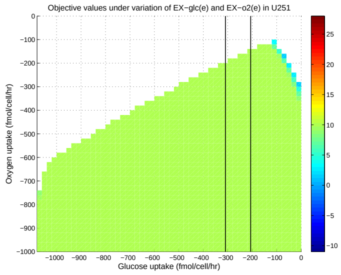
Objective values under variation of EX−glc(e) and EX−o2(e) in U251
Oxygen uptake (fmol/cell/hr)
Glucose uptake (fmol/cell/hr)

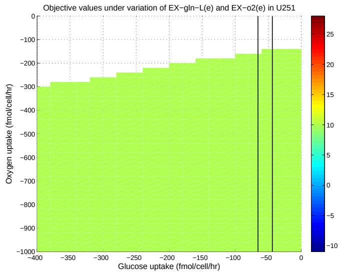
Objective values under variation of EX−gln−L(e) and EX−o2(e) in U251
Oxygen uptake (fmol/cell/hr)
Glucose uptake (fmol/cell/hr)

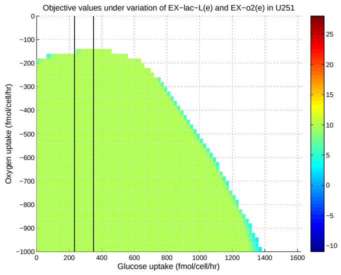
Objective values under variation of EX−lac−L(e) and EX−o2(e) in U251
Oxygen uptake (fmol/cell/hr)
Glucose uptake (fmol/cell/hr)

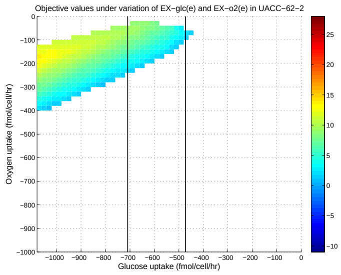
Objective values under variation of EX−glc(e) and EX−o2(e) in UACC−62−2
Oxygen uptake (fmol/cell/hr)
Glucose uptake (fmol/cell/hr)

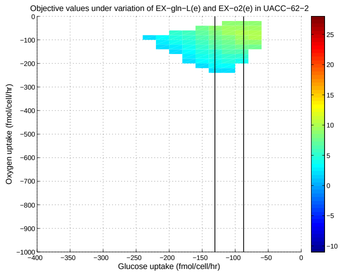
Objective values under variation of EX−gln−L(e) and EX−o2(e) in UACC−62−2
Oxygen uptake (fmol/cell/hr)
Glucose uptake (fmol/cell/hr)

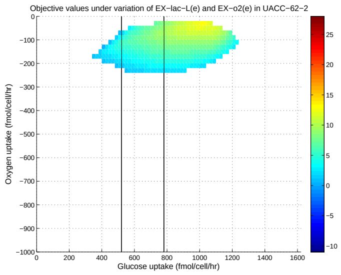
Objective values under variation of EX−lac−L(e) and EX−o2(e) in UACC−62−2
Oxygen uptake (fmol/cell/hr)
Glucose uptake (fmol/cell/hr)

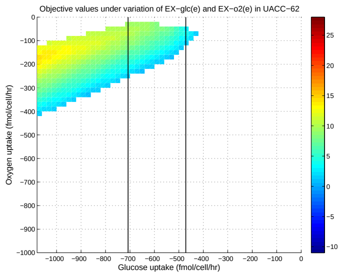
Objective values under variation of EX−glc(e) and EX−o2(e) in UACC−62
Oxygen uptake (fmol/cell/hr)
Glucose uptake (fmol/cell/hr)

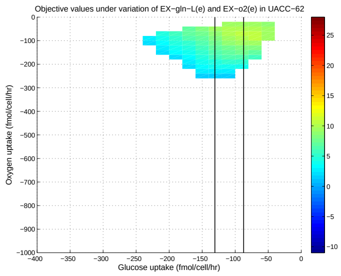
Objective values under variation of EX−gln−L(e) and EX−o2(e) in UACC−62
Oxygen uptake (fmol/cell/hr)
Glucose uptake (fmol/cell/hr)

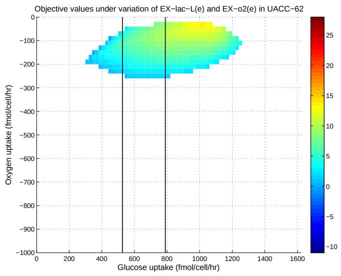
Objective values under variation of EX−lac−L(e) and EX−o2(e) in UACC−62
Oxygen uptake (fmol/cell/hr)
Glucose uptake (fmol/cell/hr)

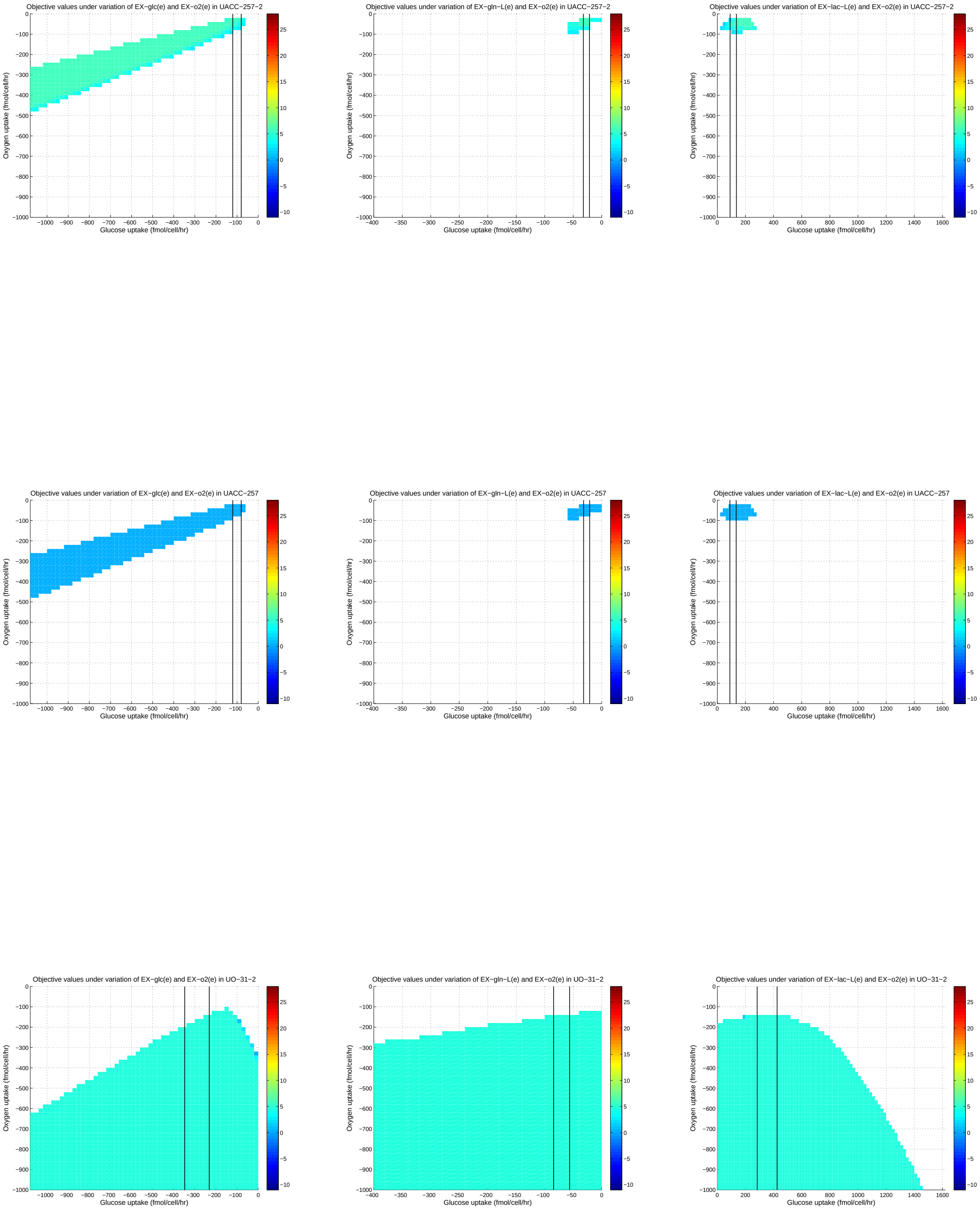
Objective values under variation of EX−glc(e) and EX−o2(e) in UACC−257−2
Objective values under variation of EX−gln−L(e) and EX−o2(e) in UACC−257−2
Objective values under variation of EX−lac−L(e) and EX−o2(e) in UACC−257−2
Objective values under variation of EX−glc(e) and EX−o2(e) in UACC−257
Objective values under variation of EX−gln−L(e) and EX−o2(e) in UACC−257
Objective values under variation of EX−lac−L(e) and EX−o2(e) in UACC−257
Objective values under variation of EX−glc(e) and EX−o2(e) in UO−31−2
Objective values under variation of EX−gln−L(e) and EX−o2(e) in UO−31−2
Objective values under variation of EX−lac−L(e) and EX−o2(e) in UO−31−2
Oxygen uptake (fmol/cell/hr)
Glucose uptake (fmol/cell/hr)

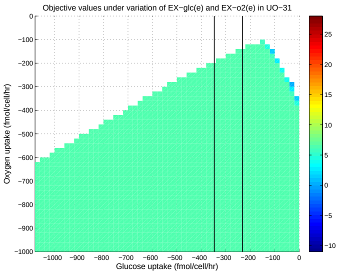
Objective values under variation of EX−glc(e) and EX−o2(e) in UO−31
Oxygen uptake (fmol/cell/hr)
Glucose uptake (fmol/cell/hr)

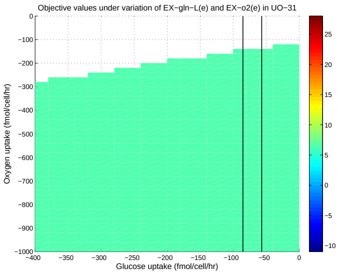
Objective values under variation of EX−gln−L(e) and EX−o2(e) in UO−31
Oxygen uptake (fmol/cell/hr)
Glucose uptake (fmol/cell/hr)

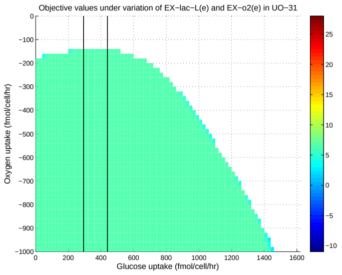
Objective values under variation of EX−lac−L(e) and EX−o2(e) in UO−31
Oxygen uptake (fmol/cell/hr)
Glucose uptake (fmol/cell/hr)

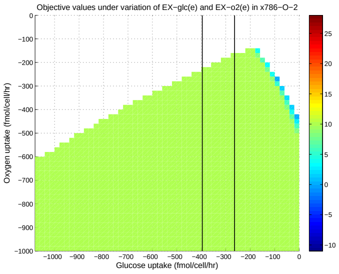
Objective values under variation of EX−glc(e) and EX−o2(e) in x786−O−2
Oxygen uptake (fmol/cell/hr)
Glucose uptake (fmol/cell/hr)

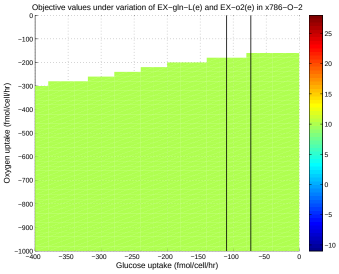
Objective values under variation of EX−gln−L(e) and EX−o2(e) in x786−O−2
Oxygen uptake (fmol/cell/hr)
Glucose uptake (fmol/cell/hr)

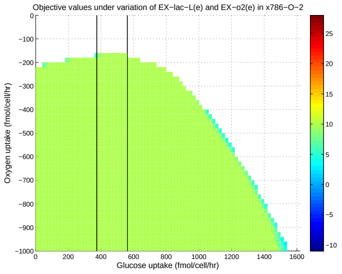
Objective values under variation of EX−lac−L(e) and EX−o2(e) in x786−O−2
Oxygen uptake (fmol/cell/hr)
Glucose uptake (fmol/cell/hr)

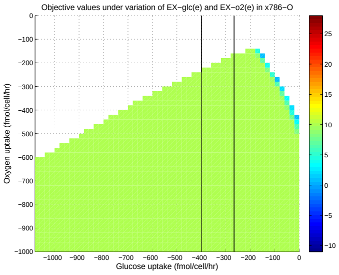
Objective values under variation of EX−glc(e) and EX−o2(e) in x786−O
Oxygen uptake (fmol/cell/hr)
Glucose uptake (fmol/cell/hr)

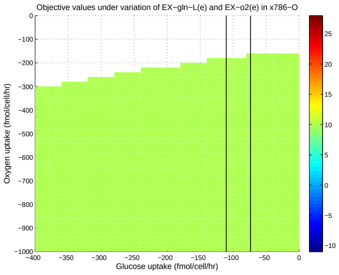
Objective values under variation of EX−gln−L(e) and EX−o2(e) in x786−O
Oxygen uptake (fmol/cell/hr)
Glucose uptake (fmol/cell/hr)

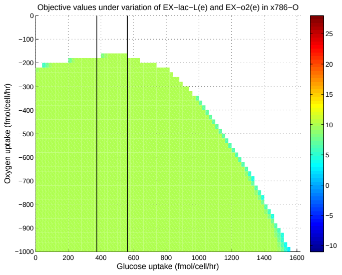
Objective values under variation of EX−lac−L(e) and EX−o2(e) in x786−O
Oxygen uptake (fmol/cell/hr)
Glucose uptake (fmol/cell/hr)

References

1. Nishiumi S, Kobayashi T, Ikeda A, Yoshie T, Kibi M, et al. (2012) A Novel Serum Metabolomics-Based Diagnostic Approach for Colorectal Cancer. PLoS ONE 7: e40459.

2. Lloyd MD, Darley DJ, Wierzbicki AS, Threadgill MD (2008) α-Methylacyl-CoA racemase–an $'obscure'$ metabolic enzyme takes centre stage. FEBS journal 275: 1089–1102.

3. Hellgren LI (2010) Phytanic acid - an overlooked bioactive fatty acid in dairy fat? Annals of the New York Academy of Sciences 1190: 42–49.

4. Ollberding NJ, Aschebrook-Kilfoy B, Caces DBD, Wright ME, Weisenburger DD, et al. (2013) Phytanic acid and the risk of non-Hodgkin lymphoma. Carcinogenesis 34: 170-175.

5. Price AJ, Allen NE, Appleby PN, Crowe FL, Jenab M, et al. (2010) Plasma phytanic acid concentration and risk of prostate cancer: results from the European Prospective Investigation into Cancer and Nutrition. The American journal of clinical nutrition 91: 1769–1776.

6. Lloyd MD, Yevglevskis M, Lee GL, Wood PJ, Threadgill MD, et al. (2013) α-Methylacyl-CoA racemase (AMACR): Metabolic enzyme, drug metabolizer and cancer marker {P504S}. Progress in Lipid Research 52: 220 - 230.

7. Mubiru JN, Valente AJ, Troyer DA (2005) A Variant of the alpha-methyl-acyl-CoA racemase gene created by a deletion in exon 5 and its expression in prostate cancer. The Prostate 65: 117–123.

8. Ouyang B, Leung YK, Wang V, Chung E, Levin L, et al. (2011) α-Methylacyl-CoA Racemase Spliced Variants and Their Expression in Normal and Malignant Prostate Tissues. Urology 77: 249.e1 - 249.e7.

9. Aurich MK, Paglia G, Rolfsson Ó, Hrafnsdóttir S, Magnúsdóttir M, et al. (2015) Prediction of intracellular metabolic states from extracellular metabolomic data. Metabolomics 11: 603–619.

10. Aurich MK, Fleming RM, Thiele I Metabotools: Intra-model analysis of extracellular metabolomic data and downstream analysis of functional phenotypes. under review .

11. Reinhold WC, Sunshine M, Liu H, Varma S, Kohn KW, et al. (2012) CellMiner: a web-based suite of genomic and pharmacologic tools to explore transcript and drug patterns in the NCI-60 cell line set. Cancer Research 72: 3499–3511.

12. Griguer CE, Oliva CR, Gillespie GY (2005) Glucose Metabolism Heterogeneity in Human and Mouse Malignant Glioma Cell lines. Journal of Neuro-Oncology 74: 123–133.

13. Hu J, Locasale JW, Bielas JH, O'Sullivan J, Sheahan K, et al. (2013) Heterogeneity of tumor-induced gene expression changes in the human metabolic network. Nature biotechnology 31: 522–529.

14. Suganuma K, Miwa H, Imai N, Shikami M, Gotou M, et al. (2010) Energy metabolism of leukemia cells: glycolysis versus oxidative phosphorylation. Leukemia & lymphoma 51: 2112–2119.

15. ZHENG J (2012) Energy metabolism of cancer: Glycolysis versus oxidative phosphorylation (Review). Oncology Letters 4: 1151.

16. O'Connor PM, Jackman J, Bae I, Myers TG, Fan S, et al. (1997) Characterization of the p53 tumor suppressor pathway in cell lines of the National Cancer Institute anticancer drug screen and correlations with the growth-inhibitory potency of 123 anticancer agents. Cancer Research 57: 4285–4300.

17. Jain M, Nilsson R, Sharma S, Madhusudhan N, Kitami T, et al. (2012) Metabolite Profiling Identifies a Key Role for Glycine in Rapid Cancer Cell Proliferation. Science 336: 1040–1044.

18. Reed JL (2012) Shrinking the metabolic solution space using experimental datasets. PLoS Computational Biology 8: e1002662.

19. Cheng T, Sudderth J, Yang C, Mullen AR, Jin ES, et al. (2011) Pyruvate carboxylase is required for glutamine-independent growth of tumor cells. Proceedings of the National Academy of Sciences 108: 8674-8679.
